# Tissue-Specific and Time-Dependent Expressions of *PC4s* in Bay Scallop (*Argopecten irradians irradians*) Reveal Function Allocation in Thermal Response

**DOI:** 10.3390/genes13061057

**Published:** 2022-06-13

**Authors:** Ancheng Liu, Xiujiang Hou, Junhao Zhang, Wen Wang, Xuecheng Dong, Jianshu Li, Xinghai Zhu, Qiang Xing, Xiaoting Huang, Jingjie Hu, Zhenmin Bao

**Affiliations:** 1 MOE Key Laboratory of Marine Genetics and Breeding, College of Marine Life Sciences, Yushan Campus, Ocean University of China, 5 Yushan Road, Qingdao 266003, China; liuancheng@stu.ouc.edu.cn (A.L.); houxiujiang@stu.ouc.edu.cn (X.H.); zjh6667@stu.ouc.edu.cn (J.Z.); ww18071001076@stu.ouc.edu.cn (W.W.); dxc18288533122@163.com (X.D.); lijianshu@stu.ouc.edu.cn (J.L.); zxh930901@126.com (X.Z.); xthuang@ouc.edu.cn (X.H.); hujingjie@ouc.edu.cn (J.H.); zmbao@ouc.edu.cn (Z.B.); 2 Laboratory for Marine Fisheries Science and Food Production Processes, Qingdao National Laboratory for Marine Science and Technology, Qingdao 266237, China; 3 Laboratory of Tropical Marine Germplasm Resources and Breeding Engineering, Sanya Oceanographic Institution, Ocean University of China (SOI-OUC), Sanya 572000, China

**Keywords:** *Argopecten irradians irradians*, *transcriptional coactivator p15*, thermal tolerance, expression regulation, function allocation

## Abstract

*Transcriptional coactivator p15* (*PC4*) encodes a structurally conserved but functionally diverse protein that plays crucial roles in RNAP-II-mediated transcription, DNA replication and damage repair. Although structures and functions of *PC4* have been reported in most vertebrates and some invertebrates, the *PC4* genes were less systematically identified and characterized in the bay scallop *Argopecten irradians irradians*. In this study, five *PC4* genes (*AiPC4s*) were successfully identified in bay scallops via whole-genome scanning through in silico analysis. Protein structure and phylogenetic analyses of *AiPC4s* were conducted to determine the identities and evolutionary relationships of these genes. Expression levels of *AiPC4s* were assessed in embryos/larvae at all developmental stages, in healthy adult tissues and in different tissues (mantles, gills, hemocytes and hearts) being processed under 32 °C stress with different time durations (0 h, 6 h, 12 h, 24 h, 3 d, 6 d and 10 d). Spatiotemporal expression profiles of *AiPC4s* suggested the functional roles of the genes in embryos/larvae at all developmental stages and in healthy adult tissues in bay scallop. Expression regulations (up- and down-) of *AiPC4s* under high-temperature stress displayed both tissue-specific and time-dependent patterns with function allocations, revealing that *AiPC4s* performed differentiated functions in response to thermal stress. This work provides clues of molecular function allocation of *PC4* in scallops in response to thermal stress and helps in illustrating how marine bivalves resist elevated seawater temperature.

## 1. Introduction

Human activities have led to massive emissions of greenhouse gases, causing global temperatures to rise. The global surface temperature in the first 20 years of the 21st century has exceeded 0.99 °C compared with that between 1850 and 1900, in accordance with the ocean temperature increasing by 0.88 °C [1]. The continued warming of the oceans drove large numbers of species out of their original habitats to extinction [2]. Moreover, warming of the seawater resulted in an increase in the abundance of cold-water phytoplankton and a decrease in the abundance of warm-water phytoplankton, which further affected entire ecosystems with implications for fish and mammal populations [3]. Specifically, coral reefs were the ecosystems most vulnerable to environmental impacts, removing substantial amounts of living coral with a warming climate [4,5]. Marine ecosystems have been significantly affected by elevated seawater temperature [6], and climate warming has also caused incalculable losses to fisheries for many years [7].

Bivalve aquaculture has developed rapidly, and global bivalve production reached 17.7 million tons, which accounted for 21.6% of world aquaculture fish production in 2018 [8], which has become an indispensable food for people globally [9]. However, the warming of seawater has threatened sustainable development of bivalve aquaculture by affecting their biological activities including growth [10,11], development [12,13], reproduction [14,15] and energy metabolism [16]. Scallops, a representative species of bivalves, have become an important aquaculture product [17]. Bay scallops (*Argopecten irradians irradians*) process the highest yield (approximate 0.8 million tons annually) of scallop production in China due to the rapid expansion of aquaculture scale since it was introduced to China for aquaculture in the 1980s [18,19]. One important reason is that bay scallops can be harvested within a year with high economic value [20]. As a tropical species, bay scallops can survive from −1–31 °C and have an optimal growth temperature of 18–28 °C [21,22]. However, cases of high seawater temperature (beyond 28 °C) in summer have been reported in recent years in scallop-rearing areas of China [23,24,25], and the elevating seawater temperature definitely impacts scallop aquaculture, resulting in mass mortality and individual miniaturization. The reason behind the phenomenon has been widely investigated at different levels. At the metabolic level, lower serum protein concentrations [22] and higher individual oxygen consumption [26,27] of bay scallops were significantly affected when the temperature was 28 °C. In genetics, scallops’ responses to environmental temperature change were driven by regulations of key/pivotal genes’ expression [28,29]. Consequently, the molecular mechanisms underlying thermal tolerance in bay scallops warrant investigation by identifying thermal-tolerance-related genetic loci/genes.

Through a genome-wide association study, we previously identified *transcriptional coactivator p15* (*PC4*) as a positive regulator of thermal tolerance in *A. irradians* and provided a candidate gene for the breeding of thermal-tolerant scallops [30]. The protein encoded by *PC4*, a general coactivator, was first discovered in human nuclear extracts in 1994 and named according to the order in which it was found [31]. PC4 has a highly conserved structure and functions extensively in eukaryotic cells and tissues, and it is also called a suppressor of TFIIB (Sub1) in yeast [32,33]. Human PC4 contains 127 amino acids, the molecular weight of which is about 15 kDa and 20 kDa in unphosphorylated and phosphorylated state, respectively [34]. Human PC4 contains two typical conserved domains, an N-terminal regulatory domain with 61 amino acids which consists of two serine-rich regions separated by lysine-rich regions, and the other C-terminal domain involves single-stranded DNA-binding and dimerization regions [34,35]. PC4 plays significant roles in RNA polymerase II (RNAP II)-mediated transcription, DNA replication and damage repair [36]. In detail, PC4 interacts simultaneously with DNA and components of transcription factor IID (TFIID) to promote the formation of preinitiation complex (PIC) [37]. In the presence of transcription factor IIH (TFIIH) and TFIID, PC4 initiates RNAP-II-mediated transcription in a stepwise series of events through promoting the formation of actively functional PIC [38,39]. Beyond that, PC4 is also critical for transcription initiation to elongation by collaborating with TFIIE-β in humans [40]. More interestingly, it has been reported that Sub1 (in yeast) is involved in the whole process from PIC formation to transcription termination [41]. PC4 is a single-stranded DNA binding protein that forms a complex with replication protein A (RPA or HSSB) on single-stranded DNA and then significantly affects the activity of HSSB-dependent enzymatic synthesis during DNA replication [42]. In addition, the ability of PC4 binding to single-stranded DNA lays a foundation for promoting DNA repair, which is necessary for the early stage of DNA damage [43,44,45]. Other studies also showed that PC4 can combine DNA ends depending on its C-terminal domain in vitro [46]. Furthermore, PC4 may be a therapeutic target for cancer [47], which plays a crucial role in activating cell proliferation mediated by the mTOR/P70S6K signaling pathway [48].

Although structural and functional investigations have been widely reported in yeast and mammals [32,49], systematic analysis of *PC4* has not been carried out on bay scallops. *PC4* provides a novel model for investigating the molecular mechanism of bay scallops’ thermotolerance via determining how the gene functions in the high-temperature seawater challenge. In the present study, we identified the number and sequences of *PC4(s)* of *A. irradians* (*AiPC4s*) in a genome-wide scale and analyzed their structures. After that, multiple sequence alignments and phylogenetic trees were constructed. Moreover, spatiotemporal expression analysis was identified in embryos/larvae at different developmental stages, in healthy adult tissues and in different tissues (mantles, gills, hemocytes and hearts) being processed under 32 °C stress at different time durations (0 h, 6 h, 12 h, 24 h, 3 d, 6 d and 10 d). Our work will contribute to further research on the functions of *PC4* and help in illustrating how marine bivalves resist elevated seawater temperature.

## 2. Materials and Methods

### 2.1. Database Mining, Gene Identification and Sequence Analysis

PC4 protein sequences of vertebrates and invertebrates (*Homo sapiens*, *Mus musculus*, *Gallus gallus*, *Oryzias latipes*, *Xenopus laevis*, *Danio rerio*, *Ciona intestinalis*, *Saccoglossus kowalevskii*, *Strongylocentrotus purpuratus*, *Apostichopus japonicas*, *Drosophila serrata*, *Bombus impatiens*, *Pomacea canaliculata*, *Crassostrea virginica*, *Brachionus plicatilis*, *Strongyloides ratti*, *Echinococcus granulosus*, *Stylophora pistillata*, *Trichoplax* sp. H2 and *Amphimedon queenslandica*) (Table S1) were derived from NCBI (http://www.ncbi.nlm.nih.gov (accessed on 10 September 2021)), Ensembl (http://useast.ensembl.org (accessed on 10 September 2021)) and OysterBase (http://www.oysterdb.com/ (accessed on 10 September 2021)) databases to determine PC4(s) in bay scallops. Next, all these sequences were used to search the transcriptome (unpublished data) and the whole-genome sequence databases (unpublished data) of bay scallops. The initial candidate sequences of AiPC4s were obtained by TBLASTN, and transcriptome and genome’s comparisons were performed through BLASTN to verify the cDNA sequences and genetic structure. We predicted amino acid sequences of AiPC4s through ORF (open reading frame) finder (https://www.ncbi.nlm.nih.gov/orffinder/ (accessed on 10 September 2021)) and DNAstar (version 4.05). Then, we blasted (BLASTP) the acquired sequences in NCBI non-redundant protein sequence database to identify the protein sequences. Finally, we acquired the coding sequence of AiPC4s after above repeated confirmation. In addition, we gained the total length of the AiPC4s, the number of exons and introns, the length of the untranslated region (UTR) and the length of ORF. Furthermore, the simple modular architecture research tool (SMART) (http://smart.embl.de/ (accessed on 15 September 2021)) was applied to detect the presence of conserved domains of proteins encoded by AiPC4s. Compute pI/Mw tool (http://web.expasy.org/compute_pi/ (accessed on 15 September 2021)) was used to predict molecular weight and theoretical putative isoelectric points (PI). The secondary of AiPC4s were obtained with Geneious 4.8.3 (http://www.geneious.com/ (accessed on 15 September 2021)), and tertiary structures of AiPC4s were predicted through Protein Homology/analogY Recognition Engine V 2.0 (http://www.sbg.bio.ic.ac.uk/phyre2/html/page.cgi?id=index (accessed on 15 September 2021)). Specially, the AiPC4 tertiary structures were visualized in PyMOL-2.5.2 (https://pymol.org/2/ (accessed on 15 September 2021)).

### 2.2. Multiple Sequence Alignment and Phylogenetic Analysis

PC4 sequences of bay scallops and orthologs from vertebrates and invertebrates, including *H. sapiens*, *M. musculus*, *G. gallus*, *O. latipes*, *X. laevis*, *D. rerio*, *C. intestinalis*, *S. kowalevskii*, *S. purpuratus*, *A. japonicas*, *B. impatiens*, *P. canaliculata*, *C. virginica*, *Chlamys farreri*, *Patinopecten yessoensis*, *B. plicatilis*, *S. ratti*, *E. granulosus*, *S. pistillata*, *T.* sp. H2 and *A. queenslandica* were aligned with the Clustal Omega program (https://www.ebi.ac.uk/Tools/msa/clustalo/ (accessed on 15 September 2021)) and color was added when identity or similarity of PC4 protein sequences reached 70% with Color Align Conservation (http://www.bioinformatics.org/sms2/color_align_cons.html (accessed on 15 September 2021)). MEGA 11.0 (https://www.megasoftware.net/ (accessed on 15 September 2021)) was used to construct phylogenetic tree, which was evaluated by 1500 replications of bootstrapping with the neighbor-joining (NJ) method.

### 2.3. Sample Collection and Heat Stress Experiment

Nine-month-old healthy bay scallops (N > 500) were derived from Laizhou (Yantai, China) and brought to our laboratory (Ocean University of China, Qingdao, China) in accordance with standard procedures [50] in 2020. In the laboratory, bay scallops were acclimated for a week prior to the experiments. Filtered and aerated seawater was maintained at approximately 22 °C, which was within the optimum temperature range for their growth [51].

In the thermal stress experiment, 160 scallops were randomly divided into groups T and C. Group T was cultured in 32 °C filtered and aerated seawater at different times (0 h, 6 h, 12 h, 24 h, 3 d, 6 d and 10 d) to assess the response of bay scallops to heat stress. Group C was used as the control group and kept in 22 °C filtered and aerated seawater. Our previous investigation showed that Arrhenius break temperature (ABT, which was regarded as an organism’s upper limit temperature) was 32.20 ± 0.25 °C [52,53], which was in accordance with reported thermal limit of bay scallops [54]. Thus, in the present study, different tissues (mantles, gills, hemocytes and hearts) of bay scallops were processed under 32 °C stress. At each high-temperature time point, three bay scallops were randomly selected from both groups and tissues of mantles, gills, hemocytes and hearts were separated, sampled and stored in liquid nitrogen for subsequent RNA-seq analysis. During the whole experiment period, the salinity and pH of the filtered and aerated seawater were 31.7 ± 0.13 and 8.09 ± 0.05, respectively.

In addition, scallop samples (N > 500) were obtained in 2019 to analyze the spatiotemporal expression pattern of *AiPC4s* with the corresponding transcriptome data that could be obtained (unpublished data). In brief, zygotes, 2–8 cells, blastula, gastrula, trochophores, D-shaped larvae, umbo larvae and juvenile scallops (N > 1000, respectively) were separately sampled after artificial hybridization of bay scallops (N > 500) in February 2016 and stored in RNAlater (Sigma-Aldrich, St. Louis, MO, USA) at −80 °C for RNA-seq analysis. Moreover, the hepatopancreas, foot, mantles, gills, gonads, kidney, smooth muscle and striated muscle of bay scallops were dissected (*n* = 4, respectively), immediately frozen in liquid nitrogen and stored at −80 °C before processing. All of the experiments were conducted according to the guidelines and regulations of the Ocean University of China and the local government.

### 2.4. RNA Isolation and RNA-Seq Analysis

Total RNA was obtained following protocols from Hu et al. [55] and then digested with DNase I (TaKaRa, Shiga, Japan). Next, the RNA-seq library construction of all samples was operated using VAHTS mRNA-seq v2 Library Prep kit (Vazyme, Nanjing, China) before Illumina sequencing. Finally, RNA-seq data (unpublished data) were extracted from embryos/larvae at eight developmental stages and eight kinds of adult tissues to determine spatiotemporal expression levels of *AiPC4s*. Similarly, RNA-seq data (unpublished data) of transcriptomes of selected tissues (mantles, gills, hemocytes and hearts) were used to detect the regulation of *AiPC4* expression under 32 °C stress [21] at different times (0 h, 6 h, 12 h, 24 h, 3 d, 6 d and 10 d).

### 2.5. Expression Analysis of AiPC4s

RNA-seq data from embryos/larvae at eight developmental stages and eight kinds of adult tissues of bay scallops were used to determine the expression profiles of *AiPC4s*. The constructed heat maps with above data had been laterally homogenized for the embryos/larvae and healthy tissues. The zygotes were used as the control for fold change in embryos/larvae at different developmental stages. Significant differences of *AiPC4* expressions were detected in certain tissues relative to the expression in smooth muscle. In the thermal stress experiment, RNA-seq data (unpublished) of transcriptomes of selected tissues (mantles, gills, hemocytes and hearts) were used to determine the expression profiles of *AiPC4s* under 32 °C stress at different times (0 h, 6 h, 12 h, 24 h, 3 d, 6 d and 10 d). Moreover, the gene expression was formalized and presented in the form of TPM (transcripts per kilobase of exon model per million mapped reads) and the fold changes were calculated as TPM_test_/TPM_control_. The control samples (Group C) were used for normalization and fold change. The presentation of the final data in the heat map was log_2_^(TPM values)^.

We constructed the heat maps with online software Lianchuan Biological Cloud Platform (https://www.omicstudio.cn/login (accessed on 20 September 2021)). Significant differences between the test and control group (smooth muscle) were determined with counts-based method DESeq2 [56] (*p* < 0.05 or |log_2_ fold change| > 1, *n* = 4) to analyze gene expression in healthy adult scallop tissues. In the heat stress experiment, the significant differences between the test and control group (group C) were determined with SPSS (version 21.0) using independent-sample *t*-Tests (*p* < 0.05, *n* = 3).

## 3. Results

### 3.1. Sequence Identification and Analysis

**Figure 1:**
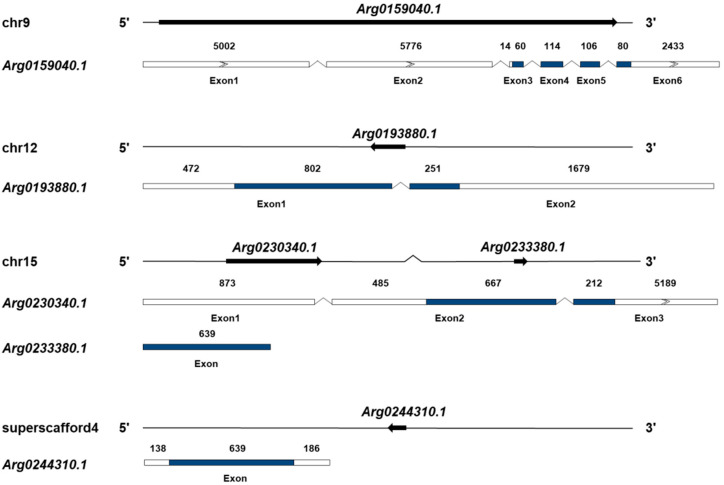
Genomic structure of *AiPC4* genes. The genomic structures of *AiPC4s* were determined by mapping cDNA sequences to genomic DNA sequences. The five *AiPC4s* are distributed into three chromosomes and one scaffold. The arrows on the lines indicate the genes located in the scaffolds. Exons in the ORF are displayed as colored boxes, 5′ and 3′ UTRs are represented by uncolored boxes, and introns are represented by broken lines. The lengths of the 5′ and 3′ UTRs and exons are relative to the cDNA sequence’s length. The number above each exon presents the exon length (bp).

Five *AiPC4s* (*Arg0159040.1*, *Arg0193880.1*, *Arg0230340.1*, *Arg0233380.1* and *Arg0244310.1*) were identified from the genome database of bay scallops (Figure 1 and Data S1), and their presence was further confirmed in the transcriptome databases. The sequences of the five genes have been submitted to GenBank (accession number ON121988-ON121992). The basic information of these *AiPC4s* is summarized in Table 1. At the genome level, the five *AiPC4s* were successfully mapped to three chromosomes (9, 12 and 15) and a superscaffold (4) (Figure 1). In detail, two genes (*Arg0230340.1* and *Arg0233380.1*) were located in chromosome 15, and another two genes (*Arg0159040.1* and *Arg0193880.1*) were located in chromosome 9 and 12, respectively. The remaining gene, *Arg0244310.1*, was only mapped on the superscaffold. The total lengths of *Arg0159040.1*, *Arg0193880.1*, *Arg0230340.1*, *Arg0233380.1* and *Arg0244310.1* were 50,697, 3,547, 10,151, 639 and 963 bp, respectively, with six, two and three exons in three *AiPC4s* (*Arg0159040.1*, *Arg0193880.1* and *Arg0230340.1*) and only one exon in the other two genes (*Arg0233380.1* and *Arg0244310.1*). Analysis of genome sequences showed that all the exon–intron boundaries in the *AiPC4s* were consistent with the GT/AG rule for splicing. In addition, the ORF of the five *AiPC4s* ranged from 360 to 1053 bp, and encoding PC4 proteins varied from 119 to 350 amino acids. The predicted molecular weights of these AiPC4s ranged from 13.566 to 39.729 kDa with PI that varied from 6.60 to 9.20 (Table 1).

**Figure 2:**
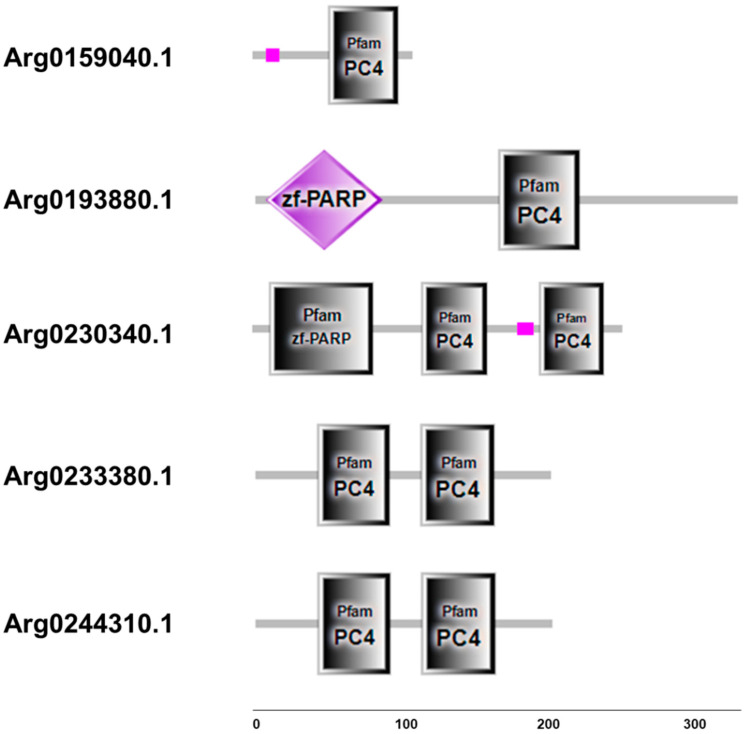
Structure and corresponding predicted protein domains of *AiPC4s*. Protein domain structure of *AiPC4s* was predicted by SMART analysis. Low-complexity region is presented in pink. PC4 domains are shown in grey boxes. zf-PARP domains are shown in grey boxes and purple diamond. Protein domains are shown relative to the length of the position in the amino acid sequences.

**Figure 3:**
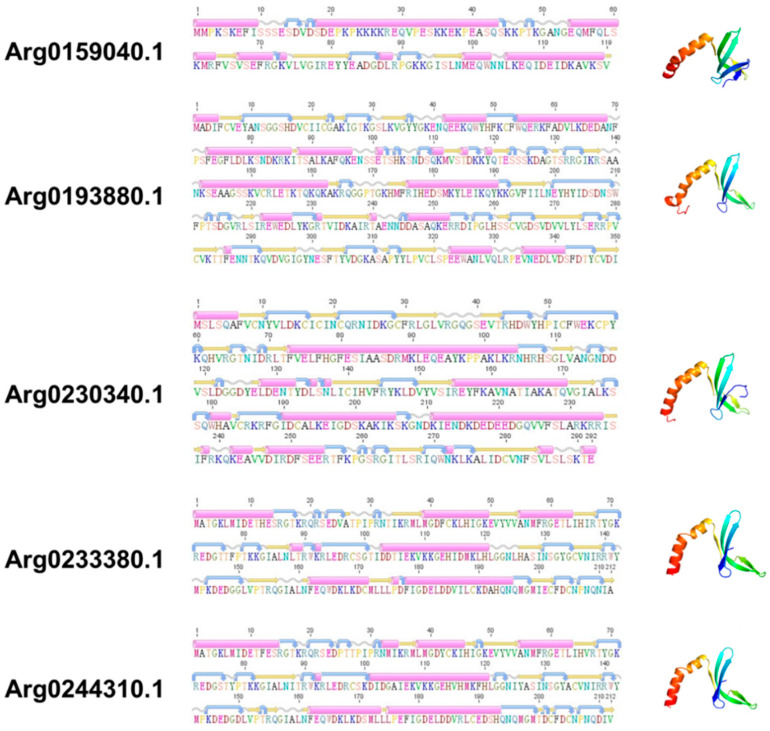
The secondary and tertiary structures of AiPC4s. The pink cylinders denote α helixes, the orange straight arrows indicate β strands, the wavy lines indicate coils, and the curved arrows indicate turns.

**Figure 4:**
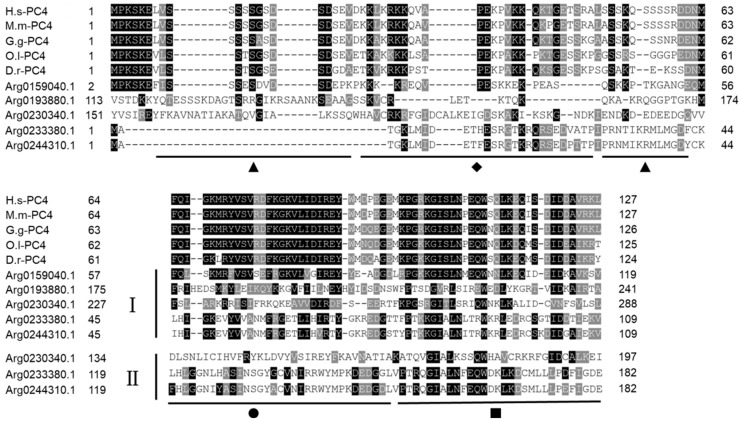
Multiple sequence alignment of *AiPC4s* from *H. sapiens* (H.s-PC4), *M. musculus* (M.m-PC4), *G. gallus* (G.g-PC4), *O. latipes* (O.l-PC4) and *D. rerio* (D.r-PC4) downloaded from NCBI. Amino acid residues that are conserved in at least 70% sequences can be stained. Conserved amino acid residues are shaded in black. The gray-shaded regions represent similar amino acid residues. Gaps are represented by dashes to improve the alignment. The ▲ represents the serine-rich region, ◆ represents the lysine-rich region, ● represents the ssDNA-binding region and ■ represents the dimerization region. I and II represent the first and the second PC4 domain in AiPC4s, respectively. Accession numbers of other species’ PC4s are listed in Table S1.

Although the sequences structure of *AiPC4s* showed some differences, several evolutionarily conserved domains were found in *AiPC4* proteins by the SMART analysis. Apparently, all five *AiPC4s* shared a functionally conserved PC4 domain, with only one in *Arg0159040.1* and *Arg0193880.1*, whereas two in *Arg0230340.1*, *Arg0233380.1* and *Arg0244310.1* (Figure 2). Besides, a low complexity region (Lcr) existed in Arg0159040.1 and *Arg0230340.1*, and another zf-PARP domain was found in *Arg0193880.1* and *Arg0230340.1*. What is more, the deduced secondary structures analysis of *AiPC4s* showed that these proteins consisted of 8, 18 15, 9 and 8 α helixes; 4, 23, 17, 12 and 14 β strands; 8, 25, 13, 12 and 11 coils; 7, 28, 20, 15 and 18 turns, respectively (Table 1 and Figure 3). The tertiary structures of *AiPC4s* were regular foldings of the polypeptide chain and are shown in Figure 3, displaying that they shared similar protein conformational structures. Specifically, according to our analysis of the multiple sequence alignment, the *AiPC4s* were highly conserved at the key sites of the ssDNA-binding region (black circle) and the dimerization region (black square), while the serine-rich domain (black triangle) and lysine-rich domain (black diamond) were relatively less conserved (Figure 4).

### 3.2. Phylogenetic Analysis

**Figure 5:**
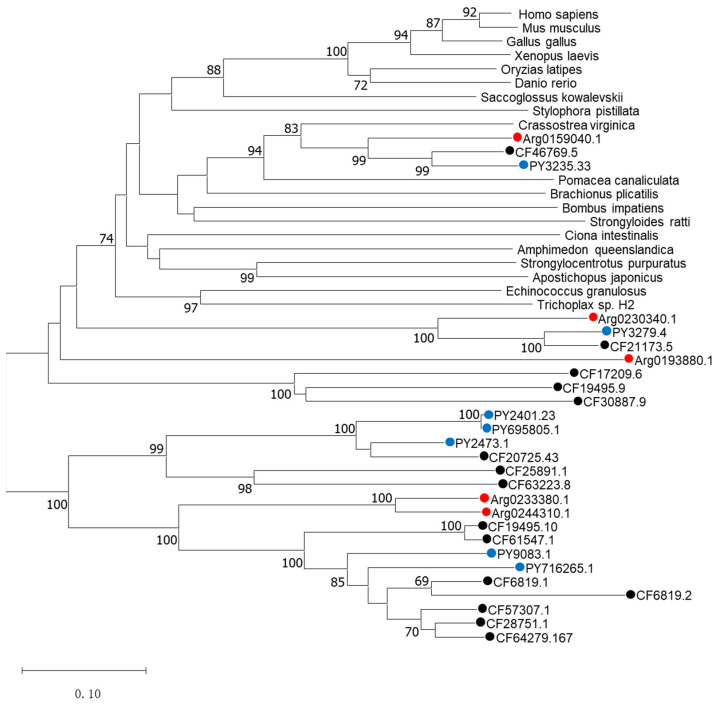
The phylogenetic tree was constructed based on the protein sequences of AiPC4s and PC4s from other species. Numbers at the tree nodes indicate the bootstrap values from 1500 replicates. AiPC4s and PC4s from *C. farreri* and *P. yessoensis* are represented by red, black and blue circles, respectively. Accession numbers of other species’ *PC4s* are listed in the Table S1.

The phylogenetic tree was constructed to determine the identities of the five *AiPC4s* using known *PC4* proteins from mammals, birds, fish, batrachian and other invertebrates by the neighbor-joining (NJ) method and shows phylogenetic relationships of *PC4s* in Figure 5. Different *PC4* proteins members generally cluster into their corresponding clades according to the phylogenetic relationships. Two distinct clades can be observed in the NJ phylogenetic tree: one is a normal clade composed of *PC4* homologs from vertebrates and invertebrates of the corresponding species, and the other is a scallop-specific clade. In the first clade, *PC4s* from vertebrates (*H. sapiens*, *M. musculus*, *G. gallus*, *O. latipes*, *X. laevis* and *D. rerio*) cluster together with a bootstrap value of 100 and the rest of the *PC4s* of the invertebrates are also clustered in a phylogenetic relationship. It was unequivocal that *PC4s* from the species including *Yesso scallops* (*PY3235.33*), *Zhikong scallops* (*CF46769.5*) and bay scallops (*Arg0159040.1*) clustered with *C. virginica* and *P. canaliculata*, forming the mollusks clade with a solid bootstrap value of 94, and then grouped with other invertebrates and vertebrates. The second scallop-specific cluster contained PC4 homologs from bay scallops, *Yesso scallops* and *Zhikong scallops*. Specifically, *Arg0233380.1* and *Arg0244310.1* clustered together, forming the bay scallop clade with a solid bootstrap value of 100 and then grouped with *Yesso scallops* and *Zhikong scallops*. All the above analyses confirmed the genetic relationship of *AiPC4s*.

### 3.3. Spatiotemporal Expression of AiPC4s

**Figure 6:**
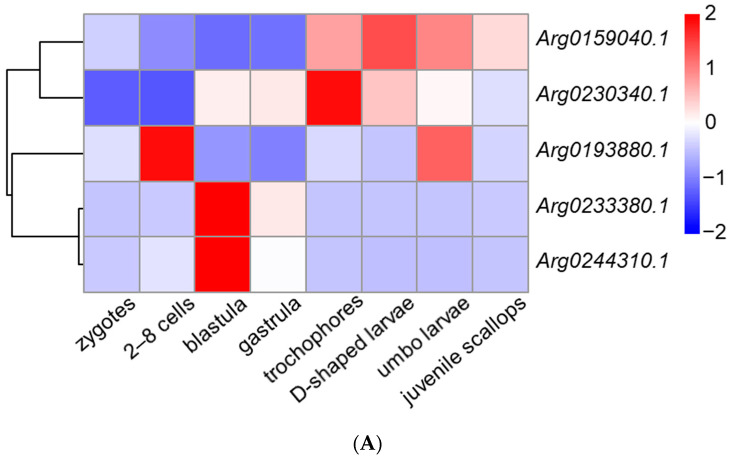

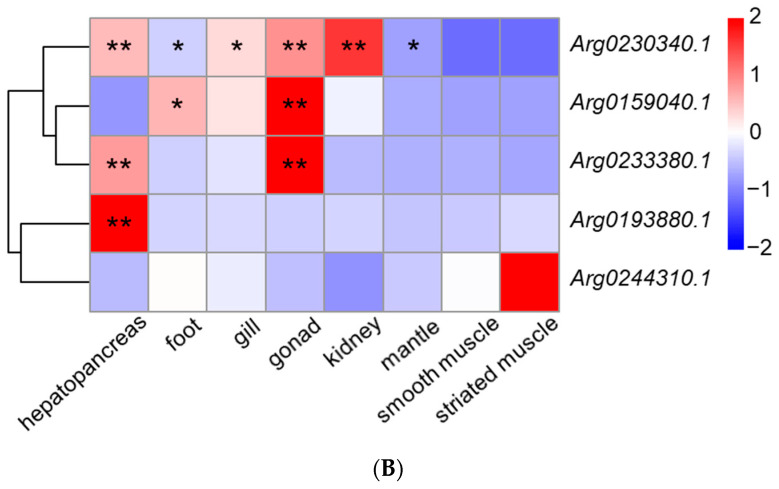
The expression profiles of *AiPC4s* from embryos/larvae at eight developmental stages and eight kinds of adult tissues have been laterally homogenized. (**A**) Relative expression levels of *AiPC4s* at different embryonic and larval stages. (**B**) Relative expression levels of *AiPC4s* in healthy adult tissues. Differently colored boxes highlight different expression patterns. Significant (*p* < 0.05) and extremely significant (*p* < 0.01) difference are indicated through “*” and “**”, respectively.

We obtained the spatiotemporal expression profiles of *AiPC4s* through RNA-seq data generated from embryos/larvae at eight developmental stages (zygotes, 2–8 cells, blastula, gastrula, trochophores, D-shaped larvae, umbo larvae and juvenile scallops) and eight kinds of adult tissues (hepatopancreas, foot, mantles, gills, gonads, kidney, smooth muscle and striated muscle). As shown in Figure 6A, the five *AiPC4s* presented different expression patterns from zygotes to juvenile scallops. The expression level of *Arg0159040.1* was the highest in embryos/larvae at nearly all detected developmental stages (the average expressions of *Arg0159040.1* were 2.14 to 68.89-fold compared with those of the other *AiPC4s* in embryos/larvae at developmental stages). In detail, *Arg0159040.1* expressed relatively low levels in embryos/larvae at early developmental stages, but initially rose from trochophores (2.17-fold) and reached the highest in D-shaped larvae (2.80-fold), then gradually decreased from umbo larvae (2.37-fold) to juvenile scallops (1.71-fold). As for *Arg0230340.1*, the expression level showed an increasing trend from zygotes (1.00-fold) to gastrula (10.16-fold) and reached the highest in trochophores (20.39-fold) but decreased from D-shaped larvae (11.96-fold) to juvenile scallops (7.46-fold). *Arg0193880.1* mainly expressed in 2–8 cells (3.31-fold) and umbo larvae (2.66-fold), and the expression of which could be observed in embryos/larvae at other developmental stages with lower levels in zygotes, blastula (0.41-fold), gastrula (0.21-fold), trochophores (0.98-fold), D-shaped larvae (0.79-fold) and juvenile scallops (0.95-fold), respectively. *Arg0233380.1* and *Arg0244310.1* displayed a similar expression pattern during all the developmental stages, with maximum expressions in blastula (106.37-fold and 36.56-fold, respectively), and followed by a moderate expression trend in the other developmental stages (0.95~24.33-fold and 0.00~5.89-fold, respectively).

The spatiotemporal expression profiles of *AiPC4s* in eight kinds of adult tissues (the hepatopancreas, foot, mantles, gills, gonads, kidney, smooth muscle and striated muscle) of healthy bay scallops were determined and shown in Figure 6B. Generally, different expression profiles of *AiPC4s* were presented in eight kinds of adult tissues. Wherein, *Arg0159040.1* presented the highest expression level in each healthy adult tissue (the expressions of *Arg0159040.1* were 6.59 to 119.57-fold compared with the total expressions of the other *AiPC4s* in each sampled tissue), which was similar to its expression profiles in embryos/larvae at eight developmental stages. The expression level of *Arg0159040.1* ranged from 0.92 to 2.97-fold in tissues compared with that in smooth muscle, with the highest expression level in gonads (2.97-fold, *p* < 0.01) and the lowest expression level in hepatopancreas (0.92-fold). Significant (*p* < 0.05) or extremely significant (*p* < 0.01) higher levels of *Arg0230340.1* were observed in hepatopancreas (122.56-fold), foot (56.38-fold), gill (103.09-fold), gonad (144.48-fold), kidney (193.54-fold) and mantle (30.47-fold) compared with that in smooth muscle. As for *Arg0233380.1*, the expression level reached its peak in gonads (44.06-fold, *p* < 0.01), followed by hepatopancreas (23.07-fold, *p* < 0.01) and other tissues (0.28 to 7.35-fold). In addition, *Arg0193880.1* showed a tissue-specific pattern, with an observing elevated level in hepatopancreas (53.76-fold, *p* < 0.01) in comparison with those in other tissues (0.00 to 2.81-fold). A similar tissue-specific higher expression of *Arg0244310.1* was also displayed in striated muscle (3.73-fold), and slight/no expression (0.00 to 1.05-fold) of which could be detected in all kinds of adult tissues. The five *AiPC4s* showed different expression patterns but were principally higher expressed in the tissues of hepatopancreas, foot, gonad and kidney. The spatiotemporal expression profiles of *AiPC4s* suggested the functions of these genes in embryos/larvae at developmental stages and healthy adult tissues in bay scallops.

### 3.4. Expression Regulations of AiPC4s with Heat Stress

**Figure 7:**
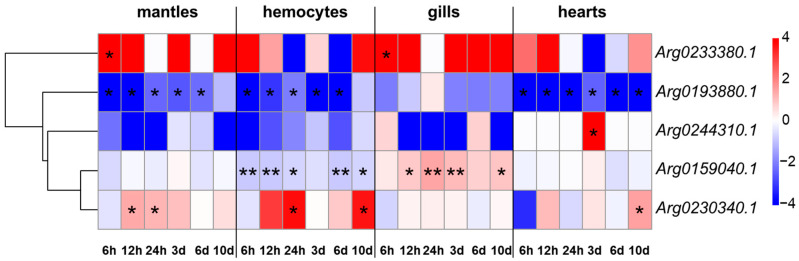
Heat map of *AiPC4* expression patterns of bay scallop tissues (mantles, hemocytes, gills and hearts) in response to high-temperature stimulation (32 °C) along different time points based on log_2_^(TPM values)^. Differently colored boxes highlight different expression patterns. Significant (*p* < 0.05) and extremely significant (*p* < 0.01) difference are indicated through “*” and “**”, respectively.

Expression profiles of *AiPC4s* were determined in different tissues (mantles, gills, hemocytes and hearts) being processed under 32 °C at different times (0 h, 6 h, 12 h, 24 h, 3 d, 6 d and 10 d) for investigating their expression regulations with heat stress (Figure 7). Generally, all *AiPC4s* displayed diverse expression regulation patterns in different tissues in 32 °C, including up- and down-regulations at different time points, showing obvious tissue-specific and time-dependent patterns. In mantles and hemocytes, the expression pattern of each *AiPC4* was similar at different challenging time points. Specifically, no expression of *Arg0233380.1* could be detected at group C, but it was obviously (*p* < 0.05) up-regulated at 6 h in mantles (1808.10-fold). *Arg0230340.1* showed obviously (*p* < 0.05) time-dependent up-regulations in mantles and hemocytes after high-temperature stimulation, including 12 (2.52-fold) and 24 h (2.35-fold) in mantles and 24 h (13.18-fold) and 10 d (12.26-fold) in hemocytes. Conversely, *Arg0193880.1* was obviously (*p* < 0.05) down-regulated at most time points in mantles and hemocytes (0.00-, 0.00-, 0.19-, 0.17- and 0.21-fold and 0.00-, 0.12-, 0.24-, 0.00- and 0.00-fold at 6 h, 12 h, 24 h, 3 d and 6 d in mantles and hemocytes, respectively). *Arg0244310.1* was generally down-regulated at most challenging time points in mantles and hemocytes (0.22-, 0.00-, 0.00-, 0.76-, 0.60- and 0.00-fold and 0.00-, 0.15-, 0.28-, 0.53-, 0.16- and 0.67-fold at 6 h, 12 h, 24 h, 3 d, 6 d and 10 d in mantles and hemocytes, respectively). Significant (*p* < 0.05) or extremely significant (*p* < 0.01) expression levels of *Arg0159040.1* were down-regulated at multiple time points with high temperature stimulation in hemocytes (0.56-, 0.58-, 0.62-, 0.58- and 0.65-fold at 6 h, 12 h, 24 h, 6 d and 10 d, respectively), but this trend was not obvious in the mantles. In gills, an up-regulation of *Arg0233380.1* could be observed at acute 6 h (3,311.56-fold, *p* < 0.05) among the up-regulated expression profile. In addition, the expression regulation pattern of *Arg0159040.1* in gills was extremely special compared with that in other tissues: (1) *Arg0159040.1* showed an up-regulation-dominated expression pattern in gills, which was distinct with those in other tissues (down-regulation-dominated expression patterns); (2) the up-regulated expression profile of *Arg0159040.1* was time-dependent, with significant (*p* < 0.05) or extremely significant (*p* < 0.01) up-regulations in acute (1.82- and 2.69-fold for 12 and 24 h) and chronic (2.21- and 1.98-fold for 3 and 10 d) periods. Diverse expression regulation patterns of *Arg0193880.1* and *Arg0230340.1* in gills could be observed compared with those in mantles and hemocytes, without significant up-regulation or down-regulation reactions. In hearts, *Arg0244310.1* and *Arg0230340.1* both displayed a single time-point-dependent up-regulation, with significantly *(p* < 0.05) up-regulated levels in 3 d (316.16-fold) and 10 d (2.82-fold), respectively. Damagingly down-regulated expressions of *Arg0193880.1* were observed at all high-temperature stimulation time points with a consistent trend of that in mantles and hemocytes. *Arg0233380.1* and *Arg0159040.1* presented up-regulation and down-regulation reactions at different time points with high-temperature stimulation (0.87-, 0.92- 1.01-, 1.22-, 0.72- and 0.91-fold and 0.10-, 2.23-, 0.69-, 1.43- and 0.91-fold at 6 h, 12 h, 24 h, 3 d, 6 d and 10d). On the whole, down-regulations mainly occurred in *Arg0193880.1* and *Arg0244310.1*, while the other three *AiPC4s* showed mixed (up- or down-) regulations with tissue-specific and/or time-dependent expressions. Overall, all *AiPC4s* were differentially expressed at one or several time points post-challenge and showed tissue-specific and/or time-dependent expression patterns with function allocation with thermal stress.

## 4. Discussion

PC4 is a functionally diverse protein with a highly conserved structure in most organisms and plays essential roles in cellular processes including RNAP-II-mediated transcription [57,58], DNA replication [42], damage repair [44,59] and cancer control [60] in vertebrates. In invertebrates, molecular functions of PC4 were only reported in the transcription activation of *Caenorhabditis elegans* [40] and in the recognition and activation of immune-related signaling pathways in the oyster *Crassostrea gigas* [61]. In our previous investigations, *PC4* genes have been proven to be involved in responses to low pH in Yesso scallops [62] and with thermal stress in Zhikong scallops [63], revealing that *PC4s* played versatile roles in scallops suffering from negative impacts. In the present study, five *AiPC4s* were successfully identified in the marine bivalve *A. irradians* from its genome [64] and transcriptome [65] databases. Moreover, protein structure and phylogenetic analyses of AiPC4s were conducted. After that, expression levels of *AiPC4s* were assessed in embryos/larvae at different developmental stages, healthy adult tissues and different tissues (mantles, gills, hemocytes and hearts) being processed under 32 °C stress at different times (0 h, 6 h, 12 h, 24 h, 3 d, 6 d and 10 d). Our work will contribute to further research on the functions of *PC4* and help in illustrating how marine bivalves resist elevated seawater temperature.

Five *AiPC4s* were identified from the genome database of bay scallops, and their presence was further confirmed in the transcriptome databases. Similarly, different numbers of *PC4* genes were detected in other bivalves, including two in *Sinonovacula constricta* [66], three in *Crassostrea hongkongensis* [67], five in *C. gigas* [68], six in *C. virginica* [69], seven in *Mercenaria mercenaria* [70], 15 in *C. farreri* [71] and up to 18 in *Mytilus galloprovincialis* [72]. Obviously, the number of *PC4s* in nearly all the above bivalves is more than those in vertebrates, for example, *H. sapiens* and *M. musculus*, which all have only one *PC4* gene. It is certain that gene expansion occurred in the *PC4* family in most bivalves. Similarly, gene expansion of *toll-like receptors* [73], *cholinesterase* [74] in Yesso scallops and *nicotinic acetylcholine receptor* [75], *superoxide dismutase* [76] in *Zhikong scallops* also occurred, indicating that functional differentiation of resistance-related genes via number expansion may be responsible for response to variable environmental factors in the ocean. The tertiary structure of *AiPC4* family members and multiple sequence alignment showed *PC4* proteins with a high identity in structure and function comparing with those from other species [32], which indicated that they might play similar functional roles in bivalves. Moreover, orthologs and paralogs of *PC4* in the NJ phylogenetic tree indicated the same origin in vertebrates and invertebrates. *PC4* members in bivalves presented a mass of species distribution caused by gene expansion, revealing the functional classification of bivalves facing various marine environment stresses.

The *AiPC4s* expressed in embryos/larvae and in adult tissues of healthy bay scallops indicate that *AiPC4s* had specific functions in all stages of development and growth of the scallop. Five *AiPC4s* had various expression patterns in embryos/larvae at different developmental stages, suggesting that *AiPC4s* performed continuous functions during scallop development. A developmental stage specific expression pattern of *Arg0193880.1* was obvious at the 2–8 cells stage, illustrating its participation in DNA replication in the early cleavage period [54]. Early cell differentiation of embryos usually occurs at the blastula stage, during which period higher expression levels of *Arg0233380.1* and *Arg0244310.1* may promote certain genes’ expression to ensure the achievement of differentiation functions of cells [77,78]. In addition, the higher expression levels of *Arg0159040.1* from trochophores to umbo larvae and developmental stage-dependent elevations of *Arg0230340.1* in trochophores might indicate the involvements of *AiPC4s* in RNAP-II-mediated transcriptional initiation of a mass of genes and the formation of organs [79]. Such various expression patterns indicated that *AiPC4s* were involved in scallop larval organogenesis and had functional differentiations during all developmental stages of scallops.

The general and tissue-specific functions of these genes could be observed in healthy adult tissues. In the present study, the ubiquitous expression of all *AiPC4s* was observed in most healthy tissues, and the relatively higher expression levels were detected in the hepatopancreas, foot, gills, gonads and kidney compared with that in smooth muscle. Hepatopancreas participated in various significant physiological activities including digestion, absorption, storage and metabolism and played an important role in initiating the immune response [80,81]. The high expression levels of three *AiPC4s* (*Arg0230340.1*, *Arg0233380.1* and *Arg0193880.1*) might promote synthesis of certain enzymes to enhance immune function in hepatopancreas [82,83,84]. The foot of adult bay scallops performed sense-related functions which might be dependent on significantly higher expressions of general coactivator *AiPC4*(*s*) for sense-related genes’ transcription [54]. Gills were regarded as the main tissue of bivalves for hematopoiesis and immune response [85,86], thus the higher level of *Arg0230340.1* in gills implied the possibility of *PC4* involved in DNA synthesis and expression of certain immune-related genes [86,87]. In addition, DNA damage usually occurred during anoxic stress and aerobic recovery in gills [88], and the high level of *Arg0230340.1* might be contributing to DNA damage repair in bivalves [89]. Furthermore, significant high expression levels of three *AiPC4s* (*Arg0230340.1*, *Arg0159040.1* and *Arg0233380.1*) were observed in the gonad that related to gametogenesis involved in reproduction [90], a process which contained DNA replication to a great extent. Besides, a significantly high level of *Arg0230340.1* in kidney ensured the realization of their immune-related functions in scallops potentially through the abundant expression of immune-related genes [91]. Consequently, the ubiquitous expression of *AiPC4s* in multiple tissues revealed an essential position in bay scallops, and diverse expression levels of *AiPC4s* ensured the realization of tissue-specific functions in scallops.

Elevated seawater temperature has severely affected the biological activity of marine organisms including scallops, resulting in massive losses to fisheries in recent years [6,7]. In order to investigate the functions of *AiPC4s* in bay scallops under high-temperature stress, expression profiles of *AiPC4s* were detected in four tissues (mantles, gills, hearts, and hemocytes), which are all regarded as the major tissues in response to thermal stress in mollusks [92,93,94]. Obviously, up-regulations mainly occurred in *Arg0233380.1* and *Arg0230340.1*, while another two *AiPC4s* (*Arg0193880.1* and *Arg0244310.1*) were down-regulated in four tissues. Similar phenomena have also been observed in the research of *heat shock proteins* in bay scallops (*AiHSP70* and *AiHSP90*) [28,95] in response to thermal stress, suggesting that the functional diversity of the above gene members coincides with thermal stress. The mantles were considered the first barrier against the adverse external environment [96], and hemocytes were the main cell mediator in molluscan internal defenses [97]. The two functional, closely related tissues interact with each other and might be involved in immune regulation through DNA repair for cell homeostasis maintenance in heat stress, which is reflected by the display of similar expression regulations of each *AiPC4* at different challenging time points between mantles and hemocytes. Another case of oyster *PC4s* with higher expression levels in hemocytes and mantles for immune regulation through DNA repair supports our hypothesis [61]. Interestingly, the expression level of *Arg0159040.1* was up-regulated in gills and down-regulated in hemocytes, respectively. The up-regulated expression might be involved in DNA repair in gills caused by heat stress, whereas the down-regulated expression might be a transcriptional function inhibition in hemocytes which was consistent with the findings in the mussels [98], corals [99] and oysters [100] that the phenomenon might be a protection mechanism against heat stress [101]. Specifically, in response to high temperature, two *AiPC4s* (*Arg0233380.1* and *Arg0159040.1*) expressed the time-dependently up-regulated levels which might promote the function of RNAP-II-mediated transcription to express enzymes associated with breathing and/or conducting DNA damage repair caused by anoxic stress and aerobic recovery resulting from strengthened respiration in the gills under high temperature [87,102,103], according to the reports in vertebrates that the elevating levels of endogenous *PC4s* mainly induced a response to DNA damage through the accumulation of *PC4* at sites of DNA damage [43]. Scallops possess a typical bivalve heart composed of two auricles and one ventricle, and the contraction of the heart drives its circulatory system, which is regarded to be critical to scallops’ normal and stressed conditions [54]. We hypothesized that the single time-point-dependent up-regulation of *AiPC4s* in hearts might improve cardiac hemodynamics as reported in rats [104] and protected heart-derived muscle as reported in humans [105] with thermal stress. As expected, the cardiac performance investigations in scallops reported that, with the increase of ambient seawater temperature, the heart rate and heart amplitude of scallops increased gradually before Arrhenius break temperature, and the results of which generally supported our hypothesis [53], whereas obvious down-regulations occurred in *Arg0193880.1* at nearly all time points during high-temperature stimulation, indicating that transcriptional function inhibition was blocked by some protein synthesis [101]. A similar phenomenon of gene family member tissue-specific and time-dependent expression was also observed in *Mitogen-activated protein kinase* (*MAPK*) [106] and *HSP90* genes in Zhikong scallops [107], providing implications for flexible regulation of bivalve gene families in response to heat stress. Collectively, our results showed a significant and flexible regulation profile (up-/down-regulated, tissue-specific, time-dependent) of these *AiPC4s* and possibly suggested a strong adaptation model under temperature stress.

## 5. Conclusions

This study represented comprehensive analyses of *PC4* family genes in bay scallops. We identified a full set of five *PC4* members from the *A. irradians* genome. After that, multiple sequence alignment and a phylogenetic tree verified their identities and evolutionary relationships. In addition, spatiotemporal expression profiles of these genes indicated the various functions of *AiPC4s* in embryos/larvae at different developmental stages and in healthy adult tissues. Expression profiles confirmed that scallop *AiPC4s* displayed tissue-specific, time-dependent and up-/down-regulations via molecular function allocation with heat stress. Future exploration on the functions and activities of mollusk *PC4s* through RNA interference, gene knock down and Western blot analysis will contribute to a better understanding the environmental adaptation of mollusks.

## Figures and Tables

**Table 1:** Sequence features of *AiPC4s* from bay scallop *A. irradians*.

	*Arg0159040.1*	*Arg0193880.1*	*Arg0230340.1*	*Arg0233380.1*	*Arg0244310.1*
Chromosome	9	12	15	15	/
Total length (bp)	50,697	3547	10,151	639	963
5′UTR length (bp)	10,792	472	1385	/	138
3′UTR length (bp)	2433	1679	5189	/	186
ORF length (bp)	360	1053	879	639	639
Number of amino acids	119	350	292	212	212
Molecular weight (kDa)	13.566	39.729	33.512	24.182	24.453
Theoretical pI	9.20	7.03	8.86	7.04	6.60
Number of exons	6	2	3	1	1
Number of introns	5	1	2	0	0
Number of α helices	8	18	15	9	8
Number of β strands	4	23	17	12	14
Numbers of coils	8	25	13	12	11
Number of turns	7	28	20	15	18

## Data Availability

The sequences of the five *AiPC4s* have been submitted to GenBank (accession number ON121988-ON121992). The original contributions presented in the study are included in the article/supplementary material. Further inquiries can be directed to the corresponding author.

## Supplementary Materials

The following supporting information can be downloaded at: www.mdpi.com/article/10.3390/genes13061057/s1, Table S1: The species and related accession numbers for homologous sequence; Data S1: Gene sequences, coding sequences and amino acid sequences of *AiPC4s*.

## References

[1] Masson-Delmotte V., Zhai P., Chen Y., Goldfarb L., Gomis M.I., Matthews J.B.R., Berger S., Huang M., Yelekçi O., Yu R. (2021). Summary for Policymakers. Climate Change 2021: The Physical Science Basis. Contribution of Working Group I to the Sixth Assessment Report of the Intergovernmental Panel on Climate Change.

[2] Wernberg T., Russell B.D., Thomsen M.S., Gurgel C.F.D., Bradshaw C.J.A., Poloczanska E.S., Connell S.D. (2011). Seaweed communities in retreat from ocean warming. Curr. Biol..

[3] Richardson A.J., Schoeman D.S. (2004). Climate impact on plankton ecosystems in the Northeast Atlantic. Science.

[4] Hoegh-Guldberg O., Mumby P.J., Hooten A.J., Steneck R.S., Greenfield P., Gomez E., Harvell C.D., Sale P.F., Edwards A.J., Caldeira K. (2007). Coral reefs under rapid climate change and ocean acidification. Science.

[5] Graham N.A.J., McClanahan T.R., MacNeil M.A., Wilson S.K., Polunin N.V.C., Jennings S., Chabanet P., Clark S., Spalding M.D., Letourneur Y. (2008). Climate warming, marine protected areas and the ocean-scale integrity of coral reef ecosystems. PLoS ONE.

[6] Domínguez R., Olabarria C., Woodin S.A., Wethey D.S., Peteiro L.G., Macho G., Vázquez E. (2021). Contrasting responsiveness of four ecologically and economically important bivalves to simulated heat waves. Mar. Environ. Res..

[7] McClenachan L., Grabowski J.H., Marra M., McKeon C.S., Neal B.P., Record N.R., Scyphers S.B. (2019). Shifting perceptions of rapid temperature changes’ effects on marine fisheries, 1945–2017. Fish Fish..

[8] FAO (2020). The State of World Fisheries and Aquaculture 2020.

[9] Gephart J.A., Henriksson P.J.G., Parker R.W.R., Shepon A., Gorospe K.D., Bergman K., Eshel G., Golden C.D., Halpern B.S., Hornborg S. (2021). Environmental performance of blue foods. Nature.

[10] Heilmayer O., Brey T., Pörtner H.O. (2004). Growth efficiency and temperature in scallops: A comparative analysis of species adapted to different temperatures. Funct. Ecol..

[11] Coleman S., Cleaver C., Morse D., Brady D.C., Kiffney T. (2021). The coupled effects of stocking density and temperature on Sea Scallop (*Placopecten*
*magellanicus*) growth in suspended culture. Aquac. Rep..

[12] García De Severeyn Y., Severeyn H., Grant W., Reverol Y. (2000). Effect of water temperature on larval development of the bivalve mollusk *Tivela mactroides*: Evaluation in the laboratory and via simulation. Ecol. Model..

[13] Enricuso O.B., Conaco C., Sayco S.L.G., Neo M.L., Cabaitan P.C. (2019). Elevated seawater temperatures affect embryonic and larval development in the giant clam *Tridacna*
*gigas* (Cardiidae: Tridacninae). J. Molluscan Stud..

[14] Martínez G., Pérez H. (2003). Effect of different temperature regimes on reproductive conditioning in the scallop *Argopecten*
*purpuratus*. Aquaculture.

[15] Maynou F., Galimany E., Ramón M., Solé M. (2020). Impact of temperature increase and acidification on growth and the reproductive potential of the clam *Ruditapes*
*philippinarum* Using DEB. Estuar. Coast. Shelf Sci..

[16] Wang X., Wang L., Zhang H., Ji Q., Song L., Qiu L., Zhou Z., Wang M., Wang L. (2012). Immune response and energy metabolism of *Chlamys*
*farreri* under *Vibrio*
*anguillarum* challenge and high temperature exposure. Fish Shellfish Immunol..

[17] Li C., Song A., Hu W. (2011). Status analyzing and developing counter-measure of cultured scallop industry in Shandong province. Mar. Sci..

[18] Wang L., Zhang H., Song L., Guo X. (2007). Loss of allele diversity in introduced populations of the hermaphroditic bay scallop *Argopecten*
*irradians*. Aquaculture.

[19] Guo X. (2009). Use and exchange of genetic resources in molluscan aquaculture. Rev. Aquac..

[20] Sun H., Yan J., Fang J. (2000). The present status, some problems and developing countermeasures in the cultivation of bay scallop in China. Pro. Fish. Sci..

[21] Zhang F. (1992). Development of bay scallops aquaculture in China. Mar. Sci..

[22] Liu S., Jiang X., Hu X., Gong J., Hwang H., Mai K. (2004). Effects of temperature on non-specific immune parameters in two scallop species: *Argopecten*
*irradians* (Lamarck 1819) and *Chlamys*
*farreri* (Jones & Preston 1904). Aquac. Res..

[23] Liu C., Sun Q., Xing Q., Liang Z., Deng Y., Zhu L. (2017). Spatio-temporal variability in sea surface temperatures for the Yellow Sea based on MODIS dataset. Ocean. Sci. J..

[24] Yan Y., Chai F., Xue H., Wang G. (2020). Record-breaking sea surface temperatures in the Yellow and East China Seas. J. Geophys. Res. Ocean..

[25] Xing Q., Wang J., Hu L., Sun Y., Huang X., Zhang L., Lu W., Wang S., Hu J., Bao Z. (2021). Seasonal variation of the thermal tolerance indicator ABT and the development of a rapid detection method in scallop *Chlamys*
*farreri*. Aquaculture.

[26] Hao Y., Yang X., Mao X. (1993). A study on the respiration of *Argopecren irradians*. Adv. Mar. Sci..

[27] Du X., Li L., Zhang S., Meng F., Zhang G. (2014). SNP identification by transcriptome sequencing and candidate gene-based association analysis for heat tolerance in the bay scallop *Argopecten*
*irradians*. PLoS ONE.

[28] Yang C., Wang L., Liu C., Zhou Z., Zhao X., Song L. (2015). The polymorphisms in the promoter of *HSP90* gene and their association with heat tolerance of bay scallop. Cell Stress Chaperones.

[29] Jiang W., Lin F., Fang J., Gao Y., Du M., Fang J., Li W., Jiang Z. (2018). Transcriptome analysis of the Yesso scallop, *Patinopecten*
*yessoensis* gills in response to water temperature fluctuations. Fish. Shellfish Immunol..

[30] Zhu X., Liu P., Hou X., Zhang J., Lv J., Lu W., Zeng Q., Huang X., Xing Q., Bao Z. (2021). Genome-wide association study reveals *PC4* as the candidate gene for thermal tolerance in bay scallop (*Argopecten*
*irradians*
*irradians*). Front. Genet..

[31] Ge H., Roeder R.G. (1994). Purification, cloning, and characterization of a human coactivator, PC4, that mediates transcriptional activation of class II genes. Cell.

[32] Lynn Henry N., Bushnell D.A., Kornberg R.D. (1996). A yeast transcriptional stimulatory protein similar to human PC4. J. Biol. Inorg. Chem..

[33] Conesa C., Acker J. (2010). Sub1/PC4 a chromatin associated protein with multiple functions in transcription. RNA Biol..

[34] Kretzschmar M., Kaiser K., Lottspeich F., Meisterernst M. (1994). A novel mediator of class II gene transcription with homology to viral immediate-early transcriptional regulators. Cell.

[35] Werten S., Stelzer G., Goppelt A., Langen F.M., Gros P., Timmers H.T.M., Van Der Vliet P.C., Meisterernst M., Werten S., Stelzer G. (1998). Interaction of PC4 with melted DNA inhibits transcription. EMBO J..

[36] Zhong L., Wang Y., Kannan P., Tainsky M.A. (2003). Functional characterization of the interacting domains of the positive coactivator PC4 with the transcription factor AP-2α. Gene.

[37] Kaiser K., Stelzer G., Meisterernst M. (1995). The coactivator p15 (PC4) initiates transcriptional activation during TFIIA-TFIID-promoter complex formation. EMBO J..

[38] Malik S., Guermah M., Roeder R.G. (1998). A dynamic model for PC4 coactivator function in RNA polymerase II transcription. Proc. Natl. Acad. Sci. USA.

[39] Wu S., Chiang C. (1998). Properties of PC4 and an RNA polymerase II complex in directing activated and basal transcription in vitro. J. Biol. Chem..

[40] Akimoto Y., Yamamoto S., Iida S., Hirose Y., Tanaka A., Hanaoka F., Ohkuma Y. (2014). Transcription cofactor PC4 plays essential roles in collaboration with the small subunit of general transcription factor TFIIE. Genes Cells.

[41] Calvo O. (2018). Sub1 and RNAPII, until termination does them part. Transcription.

[42] Pan Z., Ge H., Amin A.A., Hurwitz J. (1996). Transcription-positive cofactor 4 forms complexes with HSSB (RPA) on single-stranded DNA and influences HSSB-dependent enzymatic synthesis of simian virus 40 DNA. J. Biol. Chem..

[43] Mortusewicz O., Roth W., Li N., Cardoso M.C., Meisterernst M., Leonhardt H. (2008). Recruitment of RNA polymerase II cofactor PC4 to DNA damage sites. J. Cell Biol..

[44] Mortusewicz O., Evers B., Helleday T. (2016). PC4 promotes genome stability and DNA repair through binding of ssDNA at DNA damage sites. Oncogene.

[45] Caldwell R.B., Braselmann H., Schoetz U., Heuer S., Scherthan H., Zitzelsberger H. (2016). Positive cofactor 4 (PC4) is critical for DNA repair pathway re-routing in DT40 cells. Sci. Rep..

[46] Batta K., Yokokawa M., Takeyasu K., Kundu T.K. (2009). Human transcriptional coactivator PC4 stimulates DNA end joining and activates DSB repair activity. J. Mol. Biol..

[47] Zhang Y., Pavlov A., Malik S., Chen H., Kim N., Li Z., Zhang X., DePamphilis M.L., Roeder R.G., Ge H. (2020). Efficacy of a small molecule inhibitor of the transcriptional cofactor PC4 in prevention and treatment of non-small cell lung cancer. PLoS ONE.

[48] Su X., Yang Y., Ma L., Luo P., Shen K., Dai H., Jiang Y., Shuai L., Liu Z., You J. (2020). Human positive coactivator 4 affects the progression and prognosis of pancreatic ductal adenocarcinoma via the mTOR/p70s6k signaling pathway. OncoTargets Ther..

[49] Garavís M., Calvo O. (2017). Sub1/PC4, a multifaceted factor: From transcription to genome stability. Curr. Genet..

[50] Maeda-Martínez A.N., Sicard M.T., Reynoso-Granados T. (2000). A shipment method for scallop seed. J. Shellfish Res..

[51] Wang C., Liu B., Li J., Liu S., Li J., Hu L., Fan X., Du H., Fang H. (2011). Introduction of the Peruvian scallop and its hybridization with the bay scallop in China. Aquaculture.

[52] Nickerson D.M., Facey D.E., Grossman G.D. (1989). Estimating physiological thresholds with continuous two-phase regression. Physiol. Zool..

[53] Xing Q., Li Y., Guo H., Yu Q., Huang X., Wang S., Hu X., Zhang L., Bao Z. (2016). Cardiac performance: A thermal tolerance indicator in scallops. Mar. Biol..

[54] Shumway S.E., Parsons G.J. (2011). Scallops: Biology, Ecology and Aquaculture.

[55] Hu X., Bao Z., Hu J., Shao M., Zhang L., Bi K., Zhan A., Huang X. (2006). Cloning and characterization of tryptophan 2,3-dioxygenase gene of Zhikong scallop *Chlamys*
*farreri* (Jones and Preston 1904). Aquac. Res..

[56] Love M., Huber W., Anders S. (2014). Moderated estimation of fold change and dispersion for RNA-seq data with DESeq2. Genome Biol..

[57] Rosonina E., Willis I.M., Manley J.L. (2009). Sub1 functions in osmoregulation and in transcription by both RNA polymerases II and III. Mol. Cell. Biol..

[58] Sikorski T.W., Ficarro S.B., Holik J., Kim T.S., Rando O.J., Marto J.A., Buratowski S. (2011). Sub1 and RPA associate with RNA polymerase II at different stages of transcription. Mol. Cell.

[59] Wang J., Sarker A.H., Cooper P.K., Volkert M.R. (2004). The single-strand DNA binding activity of human PC4 prevents mutagenesis and killing by oxidative DNA damage. Mol. Cell. Biol..

[60] Peng Y., Yang J., Zhang E., Sun H., Wang Q., Wang T., Su Y., Shi C. (2012). Human positive coactivator 4 is a potential novel therapeutic target in non-small cell lung cancer. Cancer Gene Ther..

[61] Wang F., Yu Z., Wang W., Li Y., Lu G., Qu C., Wang H., Lu M., Wang L., Song L. (2018). A novel caspase-associated recruitment domain (CARD) containing protein (*Cg*CARDCP-1) involved in LPS recognition and NF-κB activation in oyster (*Crassostrea gigas*). Fish. Shellfish Immunol..

[62] Zhang J., Liao H., Xun X., Hou X., Zhu X., Xing Q., Huang X., Hu J., Bao Z. (2022). Identification, characterization and expression analyses of *PC4* genes in Yesso scallop (*Patinopecten yessoensis*) reveal functional differentiations in response to ocean acidification. Aquat. Toxicol..

[63] Hou X., Zhang J., Peng C., Yu H., Cui C., Liu A., Li J., Zhu X., Xing Q., Huang X. (2022). Molecular allocation of *PC4s* provides implications for deciphering thermal response in Zhikong scallop (*Chlamys farreri*). Gene.

[64] Wang S., Zhang J., Jiao W., Li J., Xun X., Sun Y., Guo X., Huan P., Dong B., Zhang L. (2017). Scallop genome provides insights into evolution of bilaterian karyotype and development. Nat. Ecol. Evol..

[65] Hou R., Bao Z., Wang S., Su H., Li Y., Du H., Hu J., Wang S., Hu X. (2011). Transcriptome sequencing and *de novo* analysis for Yesso scallop (*Patinopecten yessoensis*) using 454 GS FLX. PLoS ONE.

[66] Ran Z., Li Z., Yan X., Liao K., Kong F., Zhang L., Cao J., Zhou C., Zhu P., He S. (2019). Chromosome-level genome assembly of the razor clam *Sinonovacula constricta* (Lamarck, 1818). Mol. Ecol. Resour..

[67] Peng J., Li Q., Xu L., Wei P., He P., Zhang X., Zhang L., Guan J., Zhang X., Lin Y. (2020). Chromosome-level analysis of the *Crassostrea hongkongensis* genome reveals extensive duplication of immune-related genes in bivalves. Mol. Ecol. Resour..

[68] Wang J., Zhang G., Fang X., Guo X., Li L., Luo R., Xu F., Yang P., Zhang L., Wang X. (2012). The oyster genome reveals stress adaptation and complexity of shell formation. Nature.

[69] Gómez-Chiarri M., Warren W.C., Guo X., Proestou D. (2015). Developing tools for the study of molluscan immunity: The sequencing of the genome of the eastern oyster, *Crassostrea virginica*. Fish. Shellfish Immunol..

[70] Song H., Guo X., Sun L., Wang Q., Han F., Wang H., Wray G.A., Davidson P., Wang Q., Hu Z. (2021). The hard clam genome reveals massive expansion and diversification of inhibitors of apoptosis in Bivalvia. BMC Biol..

[71] Li Y., Sun X., Hu X., Xun X., Zhang J., Guo X., Jiao W., Zhang L., Liu W., Wang J. (2017). Scallop genome reveals molecular adaptations to semi-sessile life and neurotoxins. Nat. Commun..

[72] Gerdol M., Moreira R., Cruz F., Gómez-Garrido J., Vlasova A., Rosani U., Venier P., Naranjo-Ortiz M.A., Murgarella M., Greco S. (2020). Massive gene presence-absence variation shapes an open pan-genome in the *Mediterranean mussel*. Genome Biol..

[73] Xing Q., Liao H., Xun X., Wang J., Zhang Z., Yang Z., Huang X., Bao Z. (2017). Genome-wide identification, characterization and expression analyses of *TLRs* in Yesso scallop *(Patinopecten yessoensis*) provide insight into the disparity of responses to acidifying exposure in bivalves. Fish Shellfish Immunol..

[74] Xing Q., Liao H., Peng C., Zheng G., Yang Z., Wang J., Lu W., Huang X., Bao Z. (2021). Identification, characterization and expression analyses of *cholinesterases* genes in Yesso scallop (*Patinopecten yessoensis*) reveal molecular function allocation in responses to ocean acidification. Aquat. Toxicol..

[75] Shi X., Zhou Z., Wang L., Wang M., Shi S., Wang Z., Song L. (2015). The immunomodulation of nicotinic acetylcholine receptor subunits in Zhikong scallop *Chlamys farreri*. Fish. Shellfish Immunol..

[76] Lian S., Zhao L., Xun X., Lou J., Li M., Li X., Wang S., Zhang L., Hu X., Bao Z. (2019). Genome-wide identification and characterization of *SODs* in Zhikong scallop reveals gene expansion and regulation divergence after toxic dinoflagellate exposure. Mar. Drugs.

[77] Fritzenwanker J.H., Saina M., Technau U. (2004). Analysis of *forkhead* and *snail* expression reveals epithelial-mesenchymal transitions during embryonic and larval development of *Nematostella vectensis*. Dev. Biol..

[78] Lee P.Y., Davidson E.H. (2004). Expression of *Spgatae*, the *Strongylocentrotus purpuratus* ortholog of vertebrate GATA4/5/6 factors. Gene Expr. Patterns.

[79] Sastry A.N. (1965). The development and external morphology of pelagic larval and post-larval stages of the bay scallop, *Aequipecten irmdians concentricus* Say, reared in the laboratory. Bull. Mar. Sci..

[80] Stewart J.M., Brass M.E., Carlin R.C., Black H. (1992). Maximal enzyme activities of energy production pathways in the heart, hepatopancreas, and white muscle of the giant scallop (*Placopecten magellanicus*) and, lobster (*Homarus amencanus*). Can. J. Zool..

[81] Sun Z., Yang C., Wang L., Wang X., Wang J., Yue F., Liu R., Zhang H., Song L. (2014). The protein expression profile in hepatopancreas of scallop *Chlamys farreri* under heat stress and *Vibrio anguillarum* challenge. Fish Shellfish Immunol..

[82] Yue X., Liu B., Xue Q. (2011). An i-type lysozyme from the Asiatic hard clam *Meretrix meretrix* potentially functioning in host immunity. Fish Shellfish Immunol..

[83] Ren Q., Qi Y., Hui K., Zhang Z., Zhang C., Wang W. (2012). Four invertebrate-type lysozyme genes from triangle-shell pearl mussel (*Hyriopsis cumingii*). Fish Shellfish Immunol..

[84] Wu D., Hu B., Wen C., Lin G., Tao Z., Hu X., Xie Y. (2013). Gene identification and recombinant protein of a lysozyme from freshwater mussel *Cristaria plicata*. Fish Shellfish Immunol..

[85] Jemaa M., Morin N., Cavelier P., Cau J., Strub J.M., Delsert C. (2014). Adult somatic progenitor cells and hematopoiesis in oysters. J. Exp. Biol..

[86] Li Y., Song X., Wang W., Wang L., Yi Q., Jiang S., Jia Z., Du X., Qiu L., Song L. (2017). The hematopoiesis in gill and its role in the immune response of Pacific oyster *Crassostrea gigas* against secondary challenge with *Vibrio splendidus*. Dev. Comp. Immunol..

[87] Zhang D., Wang H., Yao C. (2014). Molecular and acute temperature stress response characterizations of *caspase-8* gene in two mussels, *Mytilus coruscus* and *Mytilus galloprovincialis*. Comp. Biochem. Physiol. Part B Biochem. Mol. Biol..

[88] Slobodskova V.V., Zhukovskaya A.F., Chelomin V.P. (2012). DNA damage in the gill cells of the marine scallop *Mizuhopecten yessoensis* during anoxic stress and aerobic recovery. Ocean Sci. J..

[89] Michel C., Vincent-Hubert F. (2015). DNA oxidation and DNA repair in gills of zebra mussels exposed to cadmium and benzo(a)pyrene. Ecotoxicology.

[90] Jasim M., Park K., Kang D.H., Park Y.J., Choi K.S. (2007). Comparative reproductive biology of Yesso scallop, *Patinopecten yessoensis*, under two different culture systems on the east coast of Korea. Aquaculture.

[91] Liu W., He C., Li W., Zhou Z., Gao X., Fu L. (2010). Discovery of host defence genes in the Japanese scallop *Mizuhopecten yessoensis* Jay by expressed sequence tag analysis of kidney tissue. Aquac. Res..

[92] Zhang Z., Zhang Q. (2012). Molecular cloning, characterization and expression of *heat shock protein 70* gene from the oyster *Crassostrea hongkongensis* responding to thermal stress and exposure of Cu^2+^ and malachite green. Gene.

[93] Li S., Liu Y., Liu C., Huang J., Zheng G., Xie L., Zhang R. (2015). Morphology and classification of hemocytes in *Pinctada fucata* and their responses to ocean acidification and warming. Fish Shellfish Immunol..

[94] Zhang W., Storey K.B., Dong Y. (2021). Synchronization of seasonal acclimatization and short-term heat hardening improves physiological resilience in a changing climate. Funct. Ecol..

[95] Yang C., Wang L., Wang J., Jiang Q., Qiu L., Zhang H., Song L. (2014). The polymorphism in the promoter of *HSP70* gene is associated with heat tolerance of two congener endemic bay scallops (*Argopecten irradians irradians* and *A. i. concentricus*). PLoS ONE.

[96] Shi M., Lin Y., Xu G., Xie L., Hu X., Bao Z., Zhang R. (2013). Characterization of the Zhikong scallop (*Chlamys farreri*) mantle transcriptome and identification of biomineralization-related genes. Mar. Biotechnol..

[97] Xu B., Chen M., Yang H., Zhao S. (2008). Starvation-induced changes of hemocyte parameters in the Zhikong scallop *Chlamys farreri*. J. Shellfish Res..

[98] Negri A., Oliveri C., Sforzini S., Mignione F., Viarengo A., Banni M. (2013). Transcriptional response of the mussel *Mytilus galloprovincialis* (Lam.) following exposure to heat stress and copper. PLoS ONE.

[99] Voolstra C.R., Schnetzer J., Peshkin L., Randall C.J., Szmant A.M., Medina M. (2009). Effects of temperature on gene expression in embryos of the coral *Montastraea faveolata*. BMC Genom..

[100] Yang C., Gao Q., Liu C., Wang L., Zhou Z., Gong C., Zhang A., Zhang H., Qiu L., Song L. (2017). The transcriptional response of the Pacific oyster *Crassostrea gigas* against acute heat stress. Fish Shellfish Immunol..

[101] Fu X., Sun Y., Wang J., Xing Q., Zou J., Li R., Wang Z., Wang S., Hu X., Zhang L. (2014). Sequencing-based gene network analysis provides a core set of gene resource for understanding thermal adaptation in Zhikong scallop *Chlamys farreri*. Mol. Ecol. Resour..

[102] Jiang W., Li J., Gao Y., Mao Y., Jiang Z., Du M., Zhang Y., Fang J. (2016). Effects of temperature change on physiological and biochemical responses of Yesso scallop, *Patinopecten yessoensis*. Aquaculture.

[103] Jones H.R., Johnson K.M., Kelly M.W. (2019). Synergistic effects of temperature and salinity on the gene expression and physiology of *Crassostrea virginica*. Integr. Comp. Biol..

[104] Pantos C., Mourouzis I., Dimopoulos A., Markakis K., Panagiotou M., Xinaris C., Tzeis S., Kokkinos A.D., Cokkinos D.V. (2007). Enhanced tolerance of the rat myocardium to ischemia and reperfusion injury early after acute myocardial infarction. Basic Res. Cardiol..

[105] Heads R.J., Latchman D.S., Yellon D.M. (1994). Stable high level expression of a transfected human *HSP70* gene protects a heart-derived muscle cell line against thermal stress. J. Mol. Cell. Cardiol..

[106] Liu Z., Huang X., Yang Z., Peng C., Yu H., Cui C., Hu Y., Wang X., Xing Q., Hu J. (2021). Identification, characterization, and expression analysis reveal diverse regulated roles of three *MAPK* genes in *Chlamys farreri* under heat stress. Front. Physiol..

[107] Yu H., Yang Z., Sui M., Cui C., Hu Y., Hou X., Xing Q., Huang X., Bao Z. (2021). Identification and characterization of *HSP90* gene family reveals involvement of *HSP90*, *GRP94*, and not *TRAP1* in heat stress response in *Chlamys farreri*. Genes.

