# Supplementary material for: Tissue-Specific and Time-Dependent Expressions of PC4s in Bay Scallop (Argopecten irradians irradians) Reveal Function Allocation in Thermal Response
Source: Genes (Basel). 2022 Jun 13;13(6):1057. doi: 10.3390/genes13061057 (PMC9223095; doi:10.3390/genes13061057)
Supplement: Supplementary file 1 [file genes-13-01057-s001.zip › Supplementary Data.pdf]

>Arg0159040.1

Gene sequence:

AAAATTTCAACGTTTACAGTAAGACTGAATTGAGAATCTGATCAACTTCGACATATAAA
AGACAAAAAAAAGGTATTTTTGCAAAAGCATGTATGTATTTATTTAGTGAAGATTTCCAT
TAAACCAAACCTGACATGGCAGTCTAATTTTCCACATACCCTTCCTTTATGTATTTGCAT
TAAGACAATGAATCGATGAAAATTCTCTCCAAAAAAGATAATTTTACAACATATGAACT
GTACAAAAATTGGTAGATCACTATACATCTAACTATCACTAGACTTGATCTTATACAAAC
CTTATAGGCAGCAGATAGATTTACTGAGCAGGAAATGAACCTAGCCTCTAGTATAACTA
AAAAATATTGTCATAAGTATTGATGCATTTTATACAACCGTACCTCTTCGTTAAAATCAA
AACATATCACATTTTTCATTGAAAATTCTTCATGTATAGTGTTTTTATTTTCTACAAAATT
TGACATGAATATTACATTTTTCAATTACTAGATCAAAATTAGAATTTGCATATGCATGATA
GAAATGGCAAAACAGCTGTATAACTATTTTGTGACTAAATTTCTTTTCCTCCTCAAATAG
ATCTAGAGGCCATTATGAAATCTTTAATACTGAATAACTATATAAGAAAACAACTTATCG
TAACTTATAAGTGTACCAGTTGATTCTAAACCACTCAGCTATCTGTACAACAGGGGCTT
GCTGACAATTCAGGTCCTACTGTGTCTATGTATAAATTAAAAGTCACCAGAATTAGTACA
TAGCCCCTGTGATCTGTATGTCATGACACAGAACAAGGGAGATAACTCTTAATTTTCAT
AAACTGAAAATAAAATTAATAATGATTACTAATTAATATAGTTTTATGTATACAAAAATAA
TCACATTCAAACATAAAATAATATTGTTTTACTCTTTCTTAAAAGCTTGAAAGCATTCAT
TAAAAATTTGACTAATCATTTGAAAGAAAATCCAAATCACCAAAATATACTGGTGAAAA
GAGAATCTGAAATTTCCAAATCAGATCTGACCTGTTGATAGTAAATGATCTCTAAAACC
TTTCGTAAAATATAAATCTATCCATATCTTATTTTTATCAGATTTTTATCAAATCCAATTAA
GAATTCGGTAATATCAAATTTAAAGGAACTATATATGAATATTTCTTCAATTCGACACAAT
GGAAATTACACGATGCGATTTAAAGTCCCAAAACATCTGGTATTCAAGAGGAAGTGAT
AAAAAATCTGAAATCAATGTTAAATTCTTGCCTTTGTGAAGTGTACCATGCAATGAAAC
CAATGTCAAATTCTTGTCTGTAAAGTGTATCTGTAAATAGCAAGAGCTGCTCAATACTAT
TCCAGTAACATACTGTACATCTGTACTATTACAGGCCTTCACTGAATATATTTTAATCATA
ACTATAGACCAATGGCACAGAAAACGTTCATATCTATCAGCATGAGTATTATACTATTGA
AGGGCATGCATCTGTATCATGAAGTACATGTACAAAACAACACTAATTTTCTATTGACTT
TTTTTGAACCTCTTGTTAAACTGTTATTCAATACTAAGACTTCATCTTTTACTTCAAATTA
TACAAACAGTTCATTATGTTCCGTCCGTGAATGAATATGTGAAGGATGGAACATGATCA
TTCCAGACTTTACGTTTACCCAGGTACACCGTTGGGGTCGAAAGTCCTCTTGATCTTGA
AGTTTAACCAGGTTAGTCATTTTCTCATAGTTTAGGATTAACCAGGTTAATCAAGGTCCA
CGGAACTCTGTTCCAGAATAACAATGTTCCCTCTTCACTGCATAAAATATCTACAGATTG
AAGTTTTAAAAGTCGATGATGATACGTAGAATATTTATAAGTGATTTTCATTGTTTTTTCT
TTTCATTTTCCTCCACAAGCACCACCCGTAGCAGCAGCAGCATCAGCTTGCTTCTTTAA
TTGTTTTTGTCGCATTATTTCAGCATCTCTGAAAAAGAACAACACAGATAAAGAACATG
GCACCATGGCATGATTTCATGTTGAGGAAAGAAAATCTGAAGCCCTGACTTGTATTTTC
CTAAAATAAAAATTAAAACTTTGTCATTTGGTCATGATCACTTTTTATCAAACGCTTCAA
AAGCAAGAAAAAATATTTTGCACTCTGTATTGCATTTGTATTTAAATTAGTCTTAAACTA
TATTTGGGGCTGATCGTGGACCCAATTAAGGAGTCAAGCTAGAACGAATTGCTCTGAC
AACTATGATCAATATTCAATTCACAAACCTTTCGATTATAATGTTTCATCAACTATTATTG
GTAAAATCAAAGGCAAACACAACACATGTATATAATTCTGTTCAAATACGCCGCCATAT

TTGATTTACCGGAAACACTCAACAGGTAAAATATGTTTAAAAGTTTTAAAAAGCCAAC
AAACTAAGAATAAACATGGTGAAACTGAACAAATGTAAGTACCGTATTTTCCCTATTAA
GAGCGCTAGCGCCTCCTCTAATAAGGGCGCCCCCACGTTTTTGCTGAAAAAATCAACT
AATTTCATACCATTTTTGTGCCAGATTGTGACGACTTGTCTTTGCAGACGCTAATAAAAT
ACTGTTTCACATCAATCTGATGCTTAACAGATATCCGATCAGCTATAAATTAAATTGATT
GCATTGGACAACTTAGGACATCTTTGTCAAGTGTTTACACATAGAAACAGCGAGAAGG
CCATGTTCAAAACAAAACGTGTCACTTGAATACCCCACACACGTGTCTGGATGAAGAC
TAACTGGCAACAATATTTTAGCGATGTCTATACAGGTTTTATGAATATTTACTGTATATATA
TGATAAAGGTTCCTAAATAACAGGAATGTTATCATTTCTAAGCATTTTATTGTGTGCTCT
TGTCGGTAAAAAGCAATCGAAAGCACCGTCCACCATTTTGTGTATGCATGGTAAACAA
AGAGGCTAGCATGAAATGCTAAATTCTGTGAAACAGACATATTCATACATTTCTTCATAA
TAATGTAATTTCAATACTTACTTGACTTCAAAACGTAAGTTGGTTATAGAAGTTTCAGAA
AAATACAAAACTAAAAGCAAGATGACTATATATAGTCTGTTTCAGAAAAAAACATCTAA
AAATGGTCTCAAATAACGGCCCCCTCCTAAGACATTTACCAGCCCCCGGCGTCCTCATT
AGGGAAAATATGAAAGAATTAAGCAAAAAATAATTTTTAAAATTCATGTGAAAAACTTT
GACATTATATTTCTTTCGTAGTGAACATTTGTAGGTCATTCCATATATGGTACAAGGTGCC
ATACATATATTTGGAGCGCGAGACTTTCTAACGAGAAGCATCATGGCGGCCAAGTGAGC
CGTGTCGGAGATGAGAGCCATTCAAAAGTGCTTCAAAACATAATTTAGACATGGAGGT
GGGTAAATAAATAATTATTTTATGTTTAAAATGTTTGATATTGTTGATTTTAATTACAAAAT
TACATGACACACATGAAATGCAACATTTTCACTACGGGCAATGGGAAGAAATCGTAGAT
CTGACGACGACCATCTGTCAGAAATCTTTCGTCCTTCGTTCAGAGCTACATCAACACAG
CTAGCAAGCATGCTTCCAGTATAGGGCTGAGTCGATTATTCGAATAATCAGAGTCGATT
AGATTAATGAAACTAATCGCTGATAATCGATTTAAGATTTTATTAATCGTTAATTGTAAAA
TGCATAGCAAGTGCATCTCTTTATGTACACGTTTTGAACTCAACAAAGCATATTATATGA
TGTGAGAAATACTTGTTAAATTGCGTCTATGTTGAAGTAAAAATTCATTAATCAATGAGA
AAGTGGATGTCATGAGAAAGTTTGCAAGTTGCAACACGCGGTACGCTGCAGGGGTCG
ACATTAACTTTTAGATGTACTTGTCCCTTTGGACAATGCAAGTGAAAGGTTAATTCACT
TGTCCGAATGCAATTATTACTTGTCCGACATAACTTATTTGCTGCTTTAAGTTCCATTAA
CTTAAGGTATGACATGTTATCGATTATGATACAATAACAAATGTGCAAGTGGAGTTCTTG
ATTGAAATAAAGTTTAAGTATGTAATCATGTGTCTGTTTTCTTCTGGTACCGGACAAGCG
TTAATGTCGACCCCTGCGCTGTTCGCCAACTTTCACTAGCGAAACGGCTCCATGTTGTT
ACGTCAGGGAGGCCTAATCGCCACCGGGATGCTCGGAAGCAGAAGGAGCAGTAGTCT
TAAACATTTTAATTTTACTATTGCATGTGTTTATCATAAAAAAAGTATGTTTTTTCATATTCT
TATTGAAGATAAGAGCTGTTGATTGATTTTTTTTTTTTTATCATGGAAATAAAAAAGTTC
ATTGACGAAAATTTGGTCATCCAGATTTAGTTGATTAGTCGTAGGTGTGAAGGTCATGT
CAAAACAGCGGGTTAGCAGACTGGATTTAAACGATGTTACAGAAGATGATACAGGGAC
GGAATTCAGAGCTTATTTGGGGTAAAAATGTTTGCTTTGTCAATAAATCTATTCTTATTTA
TTGGTAGGCTTATATTACGAAGGCATGTGCTCTGTCATATCTTACAGTAGGTGTTATTATT
AGTTGATATCAAAACATGGCGGACGTTTTCAGTAGTCAGTATTATCGATTACATGTGACC
GAATAATCGAATACAAAAATTACTAATCATTTCAGCCCTATTCCGGTATATACTTATTAAG
CATTAGTCTATATACAAGAGGCCCATGGGATTTAGCAGTCACCATCGGATACAGAATGA
AACAAAGACATATAAACACAACATATATATTGGCCGTTCAGGCCCTTGAATTTAGTCAA
GCGATTTTTATAAATTTGACTTTTTAATATATATGAAGAGTATTATTTGGTTCCATCAAGT
CTCTGAATTCAAAAGAAGACCTTTTTTTTTTTTTTTTTTTAAATTTTTGTCATTTTGACC

GCATTTGGCCCCGACCCACTGGTCATGGGGGGTCTGTCAGTGAGGACCGATATAGATAT
GTTAAAATGATATATTTCAGGGTAATATTTCTAACAAAGTTTGACTTAAGCTTATTTCCTA
CGAAAATTGAGTAAAAAATGCTCATAAAAGTGTTTTTCCTATATACGGTAAACCTTCGG
TTAGTTCGAACAAAATTAAATAAATATTGATGGACCAGTTTGAATTATCTGAAATTAAGC
TTCAGGAAAAAATCTTGAGTTCGAACTATCCGAGTTGAAATCAGTAAAAACCAGTTTT
GTACCGATGTACCTGGCATAAGCAATGTAAGGCAGAAAATCTATAGGGAAAATATTTAG
TTTCCTGAAGATATTATATTCAACCATTTTCTCGTAATCATGAGCATTTAATTGGTGAATT
TATAATTAATTGCGTAGCAGAAGCCGCATGCATATGTGCACGGGGACAAGGCCCCAAAC
AGCACCGTTCACGATCGCAGCCCATAATTTACATCGACGTCCGTTAACTGAGATAGAAA
CTTTATTTAAACCACAGAAAATCTAGTTAGATCTTTGTTATATTTCGTAACAAACGTAAA
AATTTTTTTTTAATTTTTTTAGTTAAAGAAAATCTGGTTGTATTTACGAAACTGCTGTTG
AGAATATTTGTTTACATTCATTTCTAAAATTAGGTCACAAGCACACTGTGACTGTCAGAT
TGTCCAAAATCCGATTGCATATTGTCTGCTTCTAAAATGATGTAACTAGTTTTAACCTGA
TGAAAAGCTACGTATCTATTGTTCAATTACCCAACACGTGGATTGTTTTGGCTGGAGCT
GAATATGTAATCACACTCACTTGCTGTGACCCAAGGCTGGCTTTTGATGTTCGCTGTGA
CACCTCGTAACTGGCCTGGCCAAGCCAGCATGAGGCACGCACATGTTTACAGGTAATT
TAACACCTTGATTGGCTGATTAAGGATAGATTGAATGCATATCTTAATGTCAACTTAAAC
TGATATCCGTATCTAGATTGGACTTCACAAACATTGGCGATTTCTTCCACGTGTTTCCAT
CAGGTACAGCGAATTTAAATATACATTGTCGGGGCCTAAGATTAAACATTTGGCCTTGC
TGCTTTGTGTACGATATATTGGCATAAATAATTTTTATTTATGGCTTAAAATGTTCATTACT
TATCTGTCGTATGGTCATAATAATGATCATTTATACAATCTTTATTCCAAACATCAAAACT
GTTGCAATATGCAATAGGGACCCCATTGCGCCGGTTTTCCGCCAAAAATCTTACTTTTA
CATTCATAATTTGACGTGTTCGAACTATCCGAAATAGGATTGATTATAAAAAGTCAGTGT
TCGAACAATCACTAGCTAAAAAATTATCGTTTTTCTGGAAAAAATTGTGTGTTCGAACT
ATCCAGCGGTTCGAATTAACCGGAGGTTTACTGTATAGTAAATTTGATCCTCTGGCCAG
GGGAAAATCCGAGATCCCAGGTCTATGAAATTCACTCCGAGATCCCAGGTCTATGAAAT
TCACAAATTTTGTAAAAAACCTTAAGACCTATGAAGATTTAAATTGTTTTAGTTATTTTG
ACTCCTTTTAGCCCAAGTCAGGTCCCCTCTGCTCCTGGGGGTCAGTCAGGACCAATATG
GCCCCCTCTCCAAGAGGTCCCAAGCTAAAGGGACCCCAGGGTCATATAATTCACAATTT
TTGTAAAGGACCTTAAGATATTTCCATCTATACAGAGTATTTGATTCTACCATTTCAGGG
GAAGATTTTTGAAGTTTTAGCCTATTTGACCCTTTTTGGCCCCACCCCTGGGGGGCTGG
GACCATTTAATTCACAATTTTGGTTGACTGTTTGGCCATGGAAGTTTTCTGCAAACTTTC
AACAAATTTGGTTCAGTAGTTTTTATTAATTATCACAAAGACTCTGACAATTAGAAAAC
ATAGCAAAATTGACTCCAAAAGTTTAATTTTGAATCACAACCATACAATGATGCTATTGA
TACAATACAAATATGCTATCCAATACATAGGTTCAGAGAGAAAGTAATTTATATGAAAAT
AGTAGCCTAATTGACCTTTTTGACCCCCGTCTATTGCCGTCTAAGGCTCCAGGGGTCAG
CCCTATCATTTGTACAATTTCAAATCCCAATAAGGATGCTACCATTGCATTATGAGCGTA
ATCCCATGTTTATTTGCAGAGGAGAAGTCATTTATATGGAAATAGCCAAATTGACTCCTT
ATGACCCTGCCCTTAAGGCCCCCGGGGGGTCAGCCCCATCATTTGTACAATTTTGGATC
CCCACCCTATAAGGATGCTACCATTGCATTATGGGTGCTATCCCATGCTTGGTTTCAGAG
AAGAAGTTGTTTATATGGAAATAGCCAAATTGACCCCTTTTGGCCCCGCTCCTCAGGCC
CCCAGGGGGTCAGCCCTATCATTTGCACAATTTTGAATCCCGCACTATAACAATGCTAC
CATTGCATTATGAGTGCTATATCTTGCTCAGTTTCAGAGCATAAGTCCTTTATATGGAAAT
AGCCAAATTGACCCCTCTTGACCCCGCCCCTCAGGCCCTGGGGAGTTAGCCCCATCATT

TGTACAATTTTGAATCCCCACCCTATAAGGATGCTACCATTGCATTATGGGTGCTATCCC
ATGCTTGGTTTCAGAGAAGAAGTCGTTTATATGGAAATAGCCAAATTGACCCCTTTTGG
CCCCGCCCCTCAGGCCCCCATCATTTGTACAATTTTCAATTAGTAGCCCATAAGGATGCT
ACCAGTCAAATTTTGTTGAAATCCGACCAGCGGTTATGGAGAAGAAGTTGATTGTTGA
CAGACGCCGGACGCTGCGGTATCCCGTAAGCTCACCTCGGTTCTTTGGACCAGGTGAG
CTAATAATGAAATGATAGCAACTTTTTAATCAGAAGCCACTTTCCGCCATTCTTCCACC
ATCGCATCATTGTAACATGGTACGTCTGCATATCTTAGACTAAAAACTGAGGTTGTATTA
GAATTTACTTTGCCATTCTGTACCTTTTATGTCTGAATAAGTTTGTTACTTTACTAAAATA
ATAAGATGAAAATTAAAAAAAAAAAATGTGCAATATCATGAGCTGCGCAAGACAATGTT
CCTTTCAAAATAATGTGTAATAATAATAATAATAATAAGACGAAGTGCAAAGCACTTCTA
AGGTAACACATACACGAAAATATAAGAAAAAACACAATCTTCAAAATTCAATCTTACAC
ACTGACAGCTTCAGTTTGCGGTCGCCATTTTTTTTATACATATGGGGAGGTTGTGCATT
GCTCCGGTACTGCTCTCCCCAGTAAATATATAATTAACTAATGCAAATGCATAATAGGAG
TAATTAAACATTTTTTTTATTTTTAAATAATAATAATAACATTATGAAAATTAAAAGCTATC
GAATGCAGTCAAAATATCCACCAAGGCGAATGAAGATGAGCACAAGGAATCATGACGT
CACATCCGTCAACAAATGGCGCGAAATCAAAGGCTTAACATACGCAGCACTCTAGGCC
TTGAATGGACACAGAGAAATCTGAATCTTTGAGTTTATTTCTTTGGGTTTATGCTAGAC
AATTATTACATCATATACTTCTATAGTTAAAGTTTATTACATCATATACTTCTATATTTAAAG
TATTTACTTTTATTTCTAAATTATAAGAGCGCAGCTCTCTGCGCGGCCGAAGGCCGCGTA
GAGCGAAGCTCTACTAACCATAACACGTGTGTGAGCATATAGAATCCAATACATTCCAG
TACCCCTTGGACTTTGAAATTGTGTCCCGCGGGAAAATTCTCGCGCCTCCATGTAGGCA
GCCATGTTTTAATTAATTCTCGTTATCAGCACGGCGAGATTTGAAATTTCTGTACTAGAC
TAAATATGTTTTCAGTATATGTTTTAAAATTACTGTAAGTAATTTTGATTTACACATATACA
AGAACAACGCAATAAATGTACTGGTCCAATATATGACGTCAGTGATGTCATAAAACCTG
ACGCCAAAGATGACGGCACAGATTCGCGCGTATTTTGATAGCATGGCATGTTGCCAAGT
TTTCTCTGCATCTGATAAAAACAGGATTGAAGTATATTGAACTATTTTGAATTCTTTCTA
ACTATCGATAGTATGTGCATGTCAGTGAAATATTTTATTATCTCGTAGAAAAATATAGACA
ATATAAAGTTATGATGTTAAAATCTTAATCTTTTACATGTAGCTGCGCTCTTCTGAGGCTT
TGCCTTAAATTCATATTATGCCTTCTACATGAAATAGAAAATAAAATAAACAATTAGAGC
TTTGCTCTTCCTATGCCGTGCATGATTCATATCAAAACATCTGGCTGCGTCCTTTAACTG
TTTAAGGCCTTGCCTTCTGTCATATTGATATCTACCTCTCTTTCCTTAGTTCCAGACTGGT
GGCCTTATTTTTATCGTCTTTTTTACCAGGACCTTTTTGTTGCTTTTTCATGTTCTTTTCT
CTGGCCTTCTCACGCTGGTTCCCACCTGAATTATGAATAAAATGTACTGGTAATATATTTA
TCTTTGAGAGTGTCTTGATGTAAATATTGCATGATGGTCAGTAAATTTGGGTAAAAAAA
AAAGGCCTACAAATTGAGATGGAGAGAATACGATATTTTTACAGAACAATAGTAAAAA
AAACTGAGATAAAATCAACGCTATGTGTAAAACAGTGATACACTTACGTGTCATTTTGA
TAGAAGGTTTGGATATAAATGATCTCAAGAGACCACAAGTTTAAAGAAACCCCTTTCA
AAACAACGTCCTGCCTCTGCCAACTAATACGTCAGATATTGCGCATGAGCATATGTTTTT
GACCAATCATTTTCTGTGTAAGTGCTGAAGCCCGAACATTTTTTCAGAAGCTAGTATCT
CTTTCTTATTTTTAGTTACGTATTTTTATGAAGCGAAAGTAGAGTTCAATATATATATATTC
AAATATATATTATATTAAAACAACAATATTAAATTGTAAGCATATATCATATTCTGTTGAGG
ATTAATAAAACCAACTAATTCAAACAAGGCAAACACCGAGAAACGGTTATTGTTAAAA
TATATCACAAGCCAAGACAGGCTTCCCTTTGATGCAGCTTTCAGCCAAAGTTCAAAGTA
TATCAAAAAAATACCATATACTATAACATCAGTAGCCGTGCTACCCCAATACTATACGTAT

TTTAAAAATATTTCTTTAAACAAAAAATAGTCTTAAAGAAGATAATTCCATGGTACTAGA
TGCGAAACCATGGCCTCATATATTTTTAACGAAAGACGTGCGAAAGTTGAAGACGAC
GGAATTCACCCTTCCAGGCCACGCCTCCAAGTGGCAACCGAAATCACGTGACCACATA
CTCCGACAACGAATGTAAACAAACACCGCGCGGCAACTTAAACGATGGCGGCCGCG
GTTTCAAACACTGTTTCTTTTACGGAAATTCCCGATGATTTTTGTATGGAATACTTTCCA
AATGTTTCATTTTCTGAAAAATGTCTGACCAAGGGCACTATTTATTTTAAAGAAGGATAT
ATTCACAATATGAAAGTGTCAAAAAATGCATCTGAAATCACCGTTGGGGCTCGTTGTTA
CCGCAGTATGAGGAAAGGAGAAAAACCACATCTTCTCAATTTGTGTATTTGTTTGGCCA
AAAAGCGCATTGAGGACTCATACTGTTCTTGCACTGCTGGGTAAATATCTAGCATGTTC
ATTTGTTGTTGTTTCTGTTTCAATAAAAACAGTGTTTCATTAGTTTATCTTGTTGTCATCA
CGTACGAACATTCATTACTTTTTTCTTCCTCTTCGTTTGAGACTTTGTTTGGTTTGGTTTA
GACAAAAATACTTCAGCACTTTGCTACAAAACATAACATTATTGACAGAAACATATTCA
ACTTGAAGAAAAATTACTCTTATTTACTGTAGAGGCTTTCTTCACATGCCTTGTTTATAA
CTTACTAGTACAGATGATCAAGAAGACACCTATACATGCTGTACTGTACAGTAGGCCTA
ATATATATTCCCCTACTGTATATCATGAACATTGTTAACAAGTACTGTACAGAAGGCTAC
AGCTAGTACCATGAGAGTTTGAAGAATCCTCATGTCATGTTTATCTGACCTTCCATCTCT
ATAATCATGATCACGAGTCACTGGATATTTCATTGGTTGCAGATGTCCACACACAGAATT
TAAAAGTTTCCAACCTAGTTAAAACTGTTTTGTTTTCTTCCGTCTCTTCATATTTATACAT
TTCACACATTAACACATGTGTATACCCTATCGACTTAAAATTATATGTACTTTTTATGTGT
AAAATGATGACAACAAATCATACAGATAAAGGACTTGACACTTTATTAGTGAACAGGT
GCTCTAAAATAGCACCTATTGCCATGAATAAACATGTACATGCCTGCTATTCTAAAGTGC
AACCGTTCAATTTACATATTTATTCAACAGTTTGTTCACAGTTTCAATTTGTTTTTTTTA
CTAAAACTAAAGCTTATATTCATTATTACCAAGGCAACACTGCAATATATCAACCATACT
GTATTATAAATGATCTTATCAGGTAAACCTACATTAACTATGTAATAAAGTGTGATCTTGT
TATCAGATAAATTGATGATGATAAATTGATATTTAAATGTGTACAGTAGTTTGTAAAATTT
CTGAACAAATTATTTTAAAGTTTTAATACCAAATGTCTTCTAGTAAATATGTATTAAGATC
AAAGTCTGGATCTTATATTCTAACTAAGTAACAATTAACAATGTTTATAAGAATGTGACA
TAACAATACATTATAGATCTGGAGGGAGATGTTCCCATATAGTGGGGCTTGTCAAGAGTA
TTCAACAACTGAAACTTCTCGGAATTACTGAAGTTCCCGCTGAACAAGCATGCACAAG
CATACCACAGACATGGCATATTCCTCGGGGAAATAAAATCAAGCCTGTGTCAGTCAACC
AGGTAGTGGTTGTAAAAGCCAAAGAAACTCATAAGAAATCGCCTATCATCCCACAAGT
TGTCAAATCTGACAGGTACAAGACAATAATTCCCATCTAACCTAAATTAATTTTAAATAT
AGTATTATTTAAGATCTTTAAGTGACATTAAATATCAGTTAACCACTATTTTGAATTAGCT
ACACATAGCATTATGAACAAAATCATAATAAATTTACAATGTAAACTTCTCTCTATTATGA
TTTTATGAAATAAGCAAAATTGTTGGCACTTCATTGCAATAGATGTGATCTTCAAAAATA
CAATGTTGTAACCATTAACACAGGTTTTTGAAACTTACTGAACAACACAAACAAATCCT
GAAAAGTGTAAAGGGTGCGCCAATATCAAATCTGAATTTAGAAGCAACACCAGTTGTA
TCCTGCAACCCTGTCCCTGTGTTACTTGGATCAGTACTGTCTTACCAGGTATGTACATAG
TATGTTAATTTACTTCTTATGACTTTTAAAGCAATTTAAGTTATGCATACATGTACTACTT
AATATAAGTTTTGAATAACAGTAAAAGACAGAGTTATTCAAAGTATTACATTAAAACAA
CATCTGTAGTTATATTTCTACTTATACTTCAAAACTGGGTTTTGCCACAGTGTGAGTAAA
CTACAAACAGTCCATACTTTATTGATGGATCCAAATATACTTGTTGGAATCAAGTCAGAG
ATTATATTAAAGGTCCTTATTCTGCGGATGGCTCTCTCAACATGAATTCGGTGTTGTGCA
ATAGTTTGAGTGATGTTGACATTAGATGAAGTTAACTGACATCCTGATGAAACAAATGG

AGGAATGTTAAGGTTTAAACCAAGATTATTAAGTTCCTCAGTAATAGTAAATCCCTTATC
TGCCATAATGGCATCACCCTTTTCAATAAAGTTTGCATCAAGAAGTTGTTGCAGTAGGG
CATAAAATCCACTCTTTGCAGTTATTTCCTTGTCCGACATGGATCCAGTGAAAAGTTCA
CTTACAAAAATCAAGTTGCCCATTGGGTCACATGCTACAAGAGCTTTCAGTGTAGTAGA
AGATTTATAATCCGAATACATTTGTGACTGTAGTTTTAAAGAAGATGGTTTCTGAGTTTT
CAACTCTGTACAATCAATAATTGCAATAGACCTTGGAAATTCTTCCTTGTATTTAGACGG
CATGTTCGTAACAATGTCATTCCGATGAGGCCAGATAGGAATAGAGCCAAGGCTAACAT
ACATATGATCAATCCATGTTAGAAATAGTTCACTCACAGCCTGCACTGAGATGGAAAAT
CTGTATGCAAGGTCTTTTAGACCAAAATTGTGACGAAGTCTCATCAATGTTAACAGAAG
TTGATTATCAAGTCCCATATTCACTATCTCTTTTCTATTATTTTGATGTTGTGGACCTTGG
GTAGTTAAAAACTGAAGCAGATTTTGAAATGTAGAATGTTTAAAGCCTGTATAGTATTC
GAGTAAACCTTCAACACGAGACTCCTCATTAATAATACTGTTCACAGTTAGTGGTGTCC
TACAAGAAGTTTCACAGCTATCAGGTATAGAACACGCAGTTGCAGTAGTAAAACTCTG
TTGCTGTTCCACGATGGAGCGGTGTTGGCACAGGACCTCATACTGGTGTAGCTGTTCTT
TAAGTCTTTTAATTTCAGCTGCGGCAGACAGAAGCTGCAAAGACGTATCTGAAATTGA
GAGAAAACAAAATTAAAACAGAATTTGTAGTTATTGATTAAGACTTTTCTATGTTTGTTT
TCAGTCTCAGAAGAATGTCCAATCTTTAAATACCAGTCAGTCAATGTCTTGTGGAGATC
CTGTTTTCCCTAAAGCTTCCAAAGTCCCATTACCACCTTGGTATCATGATTTAAAAGAG
CCATCAAGGGTAGATAACCCTCCCTACATCGAGAAAGAAACCCGTCCCCAGAGTGCCA
GCTTCCTTTGGAACCGTCTTAGAGAAAAAAGAATAACAGCATCTCAAATGCACAAAAT
CACGCAAAGGAAAAAAGGTCCCACTGAAGCTATGCTAAAAGACTTGTTTCAGACCAA
ATCATTTAGTTCTGTGGCAACTGATTATGGTAGTAGTAGGGAACAAATGGCAAGGGAAA
GGTATGTAAGCATGGGGTCAGCCAATAACCTGCATGTTCATGAATGTGGATTAGTCATC
AACAATGAATTTCCTTATCTTGCAGCCACACCTGATGGTAAGGTTTGTGATAAAGGGGA
GAGTGGCATACTGGAAATTAAATGTCCCTTTGTTGCAAGACAGATGACAATCATTGAAG
CATGCAACAAAGTGAAAAAATTTGTACTTGTTCATGATAACAATGTTATAAAAATGAAC
AGGGAACATCAGTATTATGTACAAATACAGGGACAACTTCTTGTAACAGGTGCTCCATG
GTGTGATCTTGTAGTGTATACCACCAAAGATTTGTATATTGAGAGAATTCCAGCTGATGT
TCCATTCATGACATCACTCTTACTTCAACTTTCATTGTTCTACAAATATCATGCTTTACCA
TATCTTAGAAAATAAAGCTCACCATATTTAGACACTAGCTATATGAAGCGTTACATATAC
ATGCAAGTTCATAGTTCAAATTTATAGGTAAATCTAATCAGTAGATCCTGTCTATATATGG
ATATACAGATTTATTTGTGAGATGAGTTGATTTTCTTGCTACACATCATTTCAAATAGTAT
TTTAGTAATTTGTAATGTGTCTTACTGTGGTATTATACAATGGACCTGTACATACCTGAAC
CTATATCTTGTTCATGAGGTTTTTGCAATGGTGATGGTAATGGAACATCCTGATCAGCAG
CCATATGCATGTCGACCTCTTCATCCTTATGATCAGGGTTGTCAGGGTTGTTTCTAAAAA
AGATAGGTATCATTAGCTACCAGGTAAATCATCAACCGTTTTTTTTTTTATTTAAATCAGA
CAAACTATGTATTTAATCCAAAAGAGTATAACTGAATCACATAGATAAATAAATGATATG
ATTTGTTATAATAGGTTGTAATATTAAGAACTGATAAAGCTTTTTCACCTCAGTTAAGAC
TTAAATACTTCACCTCGCACCAGTAATTAGAATAACATTATTAACTTGCAGTCTTGCAAG
GTGGTGCAAATAGAAATATGTACGTATTTTTACTCAACGACTAATCATTCATAATTTTGAT
GTGTATGTCAAACTATATAATTTATAAAATCAAAAATCACTGAGTAATAATATAATAATTG
TGTCAATGCATGTATAATTTATTGCTTCATTATGTAAAAATGTACATGATTCACAGCTATA
AATTGGTGGTAAACAATGAAGTTTATTTTGCAACCATGTTCTAAGTATCTGTTTATATGT
GTAACAGTCTCTCAAAGTTCAATTAAGAATGGTCTGGGTAATTTAAAGAAATGCTGAAA

TAATTATGAGAGAGATACAGAATAAATTTCTTGAAAAACCATGTTACTCCTGACTCTGT
CTATCTATAATGGTGTAAAAGGAATGGGATAGTGTTAATCAAAATAATATTGGCACAATC
TACCATGGCTTACATGTACTGGCGACCCTGGTTCAATTCCTAGTCTGGTCACTTCTACAT
ATTACAGCTCTGTAAGTTAGGCTTACATTAACATTAACATTCAGTGCAAATTACTAACCT
ACTCTGCTGACGTTGTCGCCAATGTGATCTGCTTCTTTGTACAGAGCTTCCATCTGCAG
ACAGAGGATCAGGAAAATCCAGTGATGGACCATCACCACCTTCAAAATGCTATAATAA
AAAAGACAGATTAACATTAGAGTGAAACTGGAAAATAGGTTTACATTACAGAACTCTAT
CTACTGTAAAGTAGCTATTTGAGTATTAAGATAGAAGCAAATTCTAGATATACTTGATATA
ATGTACCCGTATGTGCATATATATATGTGTGTGTGTATGATGTTCACACATACATGACATA
CACAGTCAGACGTTTTACACAGCTTGTAATTACATATCAGGGTACAGCAATGTAAATCT
TGTACATTTCTTGATGGTGTGTGTGCACATATCGTATGTGTGTGTGTGTGTGTGTGTGTG
TGTGTGTGGGGGGGGGGGGGGGGGGCATATTCAGTAATTCATGTGTTTCAAATGGGTA
CAGTATATCCCGAATATGTAGTGCACAATATAGGCCCGGCTTATCGGTATTACAGCAGTA
TTAATTAACCTGTTAGCATTAATATTGGGTGTAGCCTAGAACGTTTTGTCGTTCTGTCTA
CATTAAATTGTAAAATTGCTTTGCTGAATAGTGATCTTTTTATTACCCCCATCCACCCCTT
TATCGTTTTATACACAGGGCCTAATAGACGCCTAAGACGTGACCTATCTTATATATCTCTA
GTCCGCTAAACCTGCCTATATTATTAGGCTTTTTTAAAAAAAATTTTGCCTATATAGACCTA
TTTATTAGGCAAAATAAATTAAAAAAAAAAAAAAAAAAAAAAAGACTAGAGATCTAATA
ATATAGGCAGGTTTAGCGGACTAATATATCTCATCATTAGTAACGAGGCCCTGTGGTTCT
ACAGGGACAGTCACAACAAATGAGTATAACGTTACTGAAATACCCATTCAGCATTCACT
GGAAGTACTGTACATAACGGTATTTCCGTAGACTGACATTGAAATATAACTATTAAACA
ACGTTACGTGAACTGAAAACTTTCATCTTAAATAAACCATTATTGTTCTTACCTTTGAAC
ATACAAACTTGTGACGATCAATTTTCGACACGTTTAGTTGGTTATGGGGCCGTCCACAA
GCCTTAATCCATCGCAAACACTTCGCTGAATTTGTCTTTGGTTTGGGAAATGGGATAAA
ACGGACATTCTCCACCCTCTCCGGATACCTGGTATCGTTGTTACATGTCCCCCATGCACA
CGTCTTCACCATTCTTACCAATAGTCAAGATATTTCGTTATTGTTTACGTTATACAGCGTG
AAGCAGGAAGTACAGTGTACGACAAGTTGTCGGAGGTAATGGCGGACAATCGAACAC
GCGCTCTGATTGGCTAGATTTTCATAACAACCGCGTTGCTAGCAACGACCTGGAAGGG
TGAATCGAAGACGAGTAACGTAAGTTGGCTGATTGAATCAGTTTACTGTAACATAGGTC
TATCACACATTTCTGCTCTTGAAAACGATAGGCTAACATCGAATTTTAAAAGATAACTTG
AGCAAGATATGACTGCCAATAATATTTTGTCGTGCGTTAACAGGAGATGAGAATATTGT
AACCTGTTCCCGCGGATTCAGCATTCAATATTATGTTGACTGTAGGCCTATTCGTTGATC
CTTTTATTTTCACCTGCGCTGTTATATGACTTTTTATAATTGGATTAGGAATTGAAATTGA
TCTAAGAAACCTTATATTTTATGCGTCCACCACGCACTCCATGTGATTTTCTTTGTCAAG
AAAGTCTGGAGATATATGAATAAAGAAGAACGGTATTCACCGATTCAAATAGGCCTAAC
GTTACATTCGTCATGGAAATAAGAGTGATGTTGTGCATGGATTCTATTCTTCATATAAGT
GTAGGGCGTTTTACAGCCGTGCTACCCCAACTGAGAACGACACGTGTATATAAACCTCT
ACCGCTCTACCAGGTCGGGGAAAACCACCAATTTAAATTTGTGTATTATACGCAAACTT
GAGTATTATACCTCAATTGGAGTATGGATTTGTTACTAAAAATAATCTAAATTACTGAATT
TTCAAATTTTGCTATACTTTATAAAAAATAGCATTGGTATATGCCAAGATAAAGTCCAATT
TACTGAAATTTGTGCAGTAGAAGTGTTAATTGTTACTTAGGAACTTGATGCAACTTTTCT
CAAAGTATTGCATCAATTTTCAATACTTTCCGACATGGAAATAAATCTATATTTTGCATTA
GTGTTGTGTTTCATAGTTATATAAATGCGATTTGTGGAAATAGTTTTGAAAAGTTAATTTT
AAAGCAATTTCCTCTATTTTTACTTATAGTAACAAATCTTATATATACGTATATACTCCAAT

TTAGGTATAATACTCTAGTTTGCGTAAAATATGCAAGTTGGTGGTTTCCCCGACCTGTCT
ACTGCTAGTAGACTGACTAGTATATCTTTATTTTAGATAATTTTAACAAATCTTCATGTAT
TTCTATTAAAATTGCGTATGAAACTCCATATAGATCTAGCGATGCTCATAATGTAGGGAA
CCGCCATACCATGTCTCAGCTGCTGGCCTTGTGCAAAAAGTAAAAACACACATGGGAT
TTATAAGATGAGTCCTAAACTGTCCTGTATTTAGGATTTTCATTAAGTGTTTTGTAATATT
ATTACAGCAAAACTTACAAGCTACAAGGTTAGTGGCATTTTTAACCATACTGTTATATAT
AATTAGCCATCAGCTTGGATCTGGTCGCAGGGGTGTAAACATTTATTTCAAAGCACTAG
CCCAGGGCTAGTAGACACCAAAATTTCACTAGCCCGGCCTAAATATCTACTAGCCCAGC
AAAAAAAAAATTGGAAAATTTATAATACCAGTATTTTTTATACTGTATAAATTCAAATATT
TTTAATTGAGTGAGTTTTTTTTAATTATTTGCATCTAGTTTTTTTTTAAATTCAAATAAAC
ATTAAGAAAATCTTTAATCTGTGTTATTTCTAGAACACAATTTTTAGATTTTTTTAAATTC
TGAAACCGGATTCTGGATTCCCGACCTTTGACCCGGATATTAGAATAACCATAACAAAT
ATGGCTGCCTGCTGCAGGCATGGCTTTGCTGCCGACTTTTGGAGGAATAGATTCCAGTT
CTTTAACATCGTTTTTGTAACTATTAAAATTTACTGATCTGATAGCTGAGATGACTTATTT
GATGGTTTGTATATTGTTTTTCTAGAACTTGTGTTGCAAATTACGTAACTTTCACATGAA
ATGCTATGCATCACTATTCACTGTGGTGATCGGGAGGTCTACTGTACCTGTCTACAGTCG
TAAATAAATTACACAACATGACTCCAGATTAAGATTTAAAGTTAGATACGAATATTACAT
AGAATTGCAATGTTTTAATTTTAAAGTGTTATTATGACTTAATATTGATGTTTTTCGTTTT
GAAATATCTTTGAAAAAAGTCACTAGCCCCGGGCTAGTATGGCTAAAATTTCCACTAGC
CCAAGCTGAAAATTTGGTAGCCCCGGGCGTCGGGCTAGTGGATTTTTACACCCCTGCT
GGTGCCGTACAGGTTGTGAGTTTACTCACAATATGATTATTGCAAGCAAAATATACCGAT
ATTAGATTTTTTTCAGCATTTTCAGCCCTAAGAAAAAATACATTTGCAAAGTACACTGG
TGTGTACACAAGTTTTTACGGTATTTGTAACAATTTACCTGTGGATTGGTGACAGTACTC
GAGTTAAGGATGTCACGGTTCACGTTTTTTCAGCTCGGTTCGGTTTCAATATTTTTTTAT
CGATTTTCGGTTTTTTCGGTTCGGTTCTGACTAATCTTACTTAAACACTATTTAAGTCTAT
TTACTATCAAAAACAAGTTTTACTTTAGATTTAAAAAGATGGTACATCACTCAAACAAA
TGTTTTTAGCTCACCTGGCCCAAAGGGCCGGTGAGCTATTGCCATGGCGCGGCGTCCG
TCGTCCGTGCGTCGTCCGTCCGTCGTCCGTCCGTCAACATTTCCTTTAAATCGCTACTT
GTCCTAGAGTTCTGCATGGATTTTAACCAAATTTGGTCAGAAACTCTCTTAGGGGAAGG
GGATCAGATTTTGCATAAATGGTGACCCTGACCCCCCTGGGGCAGGAGGGGCGGGGCC
CAATAGGGGTAATAGAGGTAAATCCTATAAAACACTACTTGTCGTAGAGTTCTGCATGG
ATTGCAACCAAATTTGGCCAGAAACTTCCTTAGGGGAAGGGGATCAGATTTGCATAAA
TGGTGACCCTGACCCCCTGGGGCAGGAGGGGCGGGGCCCAATAGGGGTAATAGAGGT
AAATCCTATAAAACGCTACTTGTCCTAGAGTTCTGCATGGATTGCAAACAAATTTGGCC
AGAAACTTCCATAGGGGAAGGGGATTAGATTTTGCATAAATGGTGACCCTGACCCCCC
CTGGGGCAGGAGGGGCGGGGCCCAATAGGGGTAATAGAGGTAAATCCTATAAATCGCT
ACTTGTCCTAGAGTTCTGCATGGATTGCAACCAAATTTGGCCAGAAACTTCCTTAGGGG
AAGGGGATCAGATTTTGCATAAATGGTGACCCTGACCCCCCTGGGGCAGGAGGGGCGG
GGCCCAATAGGGGTAACAGAGGTAAATCCTATAAATCGCTACTTGTCCTAGAGTTCTGC
ATGGATTGCAACCAGATAAGGCCAGAAACTTCCTTAGGGGAAGGGGATCAGATTTTGC
ATAAATGGTGACCCTGACACCCCGGGGCAGGAGGGGTGGGGCCCAATAGGGGTAATA
GAGGTAAATCCTATAAATCGCTAATTGTCCTAGAGTTCTGCATGGATTGCAACCAAATTT
GGCCAGAAACTTCCTTAGGGGAAGGGGATAAGATTTTGCATAAATGGTGACCCTGACC
CCCCTGAGGCAGGAAGGGCGGGGCCCAATAGGGGTAATAGAGGTAAATCCTATAAAAC

GCTACTTGTCCTAGAGTTCTGGATGGATTGTAACCAAATTTGGCCAGAAACATCCTTGG
GGGAAGGGGAACAGAACTTGTATAAATTTTGGCTCTGACCCCCCGGGGGCAGGAGGG
GCGAGGCCCAATAAGGGAAATAGAGGTAAATCCTATAAATCGCTACTTATCCTAGCGTT
CTGGATGGATTGTAACCAAATTTGGCCAGAAACATCCTTGGGGGAAGGGGAACAGAA
CTTTTATAAATTTTGGCTCTGACCCCCCCGGGGGTAGGAGGGGCGGGCCCAATAGGGG
AAATAGAGGTAAATCCTATAAATCGCTACTTGTCCTAGAGTTCTGCATTGATTGTAATCA
AATTTGGCCACAAACATCCTTTGGGGAAGGGAAACAAAACTTGTATAAATTTTGGCTC
TGACCCCCCTGGGAGCAGGAGGGGTGGGGCCCAATAGGGGAAATAGAGGTTAATCCTA
TAAATCGCTACTTGTCCTAGAGTTCTGCATGGATTGCAACCAAATTTGGCCAGAAACAT
CCTTGAGGTTAACAGAATTCGTATAAATTTTGGCGTTGACCCCCTGGAGCAGAAAGAG
TGGGGCTCAATAGGGGAATTAGATGAAAATATTCAAATTCCTTTAGAAAAGAAACAATG
AACTTATATTCAGAACATTATATAGCATTACAAACCAGGTGAGCGATACAGGCCCTCTG
GGCCTCTTGTTCCTCTATCAAAATTTTATTTCGATTAAAAAACATTAAAAATAATTAATTG
AGTCCTGAAAAATATCAACGGCGCAATATCCTATATATGGAATAAAGTACTGATTGTGCA
TGCAACAAAAAAAAAAAAAAAATACATCATTTTATAGACATATAACACGATTTAATACCA
ATTTTTGTTCAAATGTTGAGTATCATTTATGATCTGTCGGCAGTGGAACACCTTTAAACA
GACATAATTAAACATTAACTGATCTTTTTAACTCTTTCAGTATTCTTGACGCATTTAAAG
TCGCAGAGACAAGTCCTCGTATCATTTATGATACATTATTCCATCACTAAAACATTTCCC
CGAACTGTATCCTTCAAGACGCATTATTACCTCATGTACCGCAAATCAATACATCGGTAT
TATCTCGAAAAAGTGTTTAAAAGAAGTAATATACATTGGAATTATAATAAAGATCAACAT
TATTATGTACCAGGTGCATGTTCAATCCTAAAATGGTTTAATTCACGGATTATTGGCTGA
CGGAAACGGGATTGTTTACAATCGCAACACAATGGCGGCCTGATTTAGCTGCCGATATT
CTGCGGGATTGTTATAAAAAAAAATATAATAAAAACATCAACAATTATAATATATCAAAC
AATAAGTGAGATATATCATTTAGATATTATGTATGTGCAAAAAAATGTCCGCAGACAGCA
AAAAACGATCTTCTGAATGACTGAACTTGCACACATGTTACACATATATGTCAGTCAAC
TGGGGTATTTTTCGGGATTCTGACAAAAATCTCGCCGTATTTTCAATGAAATAATCAGCA
ATACAGTTTATACAACACGTACATTACAAGTGAAATAGGAGATTTAATTTTCTAATGAAC
CAAACCGAAACTAAGAATCATCGTACCGAACTGAACCATACCGTTGAATATTTCGGTTA
ACCGTATTTTCGGCTAACCGTGACACTCCTTACTCGAGTCTATAAATAACAAGCTACAC
TTATTCAATACATCGATACAAGTCAGCGGCAGAGCGGTATGGGTTTATATACACACTTCG
TTCTCAGTTGGGGTAGCACTGCTGTAATTAAACCACTCCAAGTGAAGGCTATAGTGAGA
TTGAGATCAGACTTCCTCGGAGAGAAAACCTGGCCATGGTCGATGTTTAAGGGGGAAT
ATGCAGTTACACATTTGAAAGTGGGAGTAGTCTAGCAGGTTTTATTTGTAAATAGTTGG
CACTACAGGCACTGGTAACCCAAAATGTGTTAATCTTGCCGAAACTTGTTAGAATAACG
GCAGAATACTTAATTTTTAACAAGTTTCGGCAGGATTAGCATATGTGCTTTTTTATTCAG
TATTAACCCTTTATATTAAGTCTTTTCCTCAGAATTCCAATTCGATTAATAAAATTAAGTA
TTTCTGCCGTAAAATTAAGTATTTTTCTGTCCGCCGTGGGTCGTGACGGCTGACGGTCG
AATAACAGAGGGTATCAGTCACAATCACGGCTATTTATTGCTACATTGTCGGAGGTTGG
GAATCGCCAAACTTTTCCGATGCGACGGTGAAATTAACAGATTCGGCGATAGAATTAAG
AGTTTCTGCGTTTATATTCAGTGTTTCTGCGTTTATATTCAATGTTTCTGCGCTAATATTA
ACACATTTCGTCGCTTACATCGAGCGGGCGCGCCGAGCCGTGACGGCCGAGCACACG
GCTTACACAGGTAAACTTTACCTTGTGAAACGAGACTTACAGTACGTACAGCCGCCAG
CCGATTGTACATGTAAAATATTTATGTTTACAATTCTCTTTTCCCCTTTAAAAATACATATA
TCCTCATTATCCTATTCTCCTATCTTTTGTTGTTTACTTTCCCGCGTCTCGTCATTTGAGCT

ATTCATTTTGCACCTTTTTTCGGAAGAGTTCCGATACGAGCGGTAACAAATGAATAACT
GTCAAGCGGTAACAAATGAATAACTGTCTAGGTTATGTGTAAAATATGTGTATATACACG
GGGATTAACTATGACAAACAAATTATGTGTTTGATTATGTGCAATTACATGTATGTATATT
CTTCTCACAAATTTGAATTGTTGACGATATTAAAATAAGATCGTTTTCCATAAAATAAAT
AAATATTTGTTATTTACAGACGTTTTGAAAGATATTTTGCTGTTGCCTTTAATTGTAAAAT
GCTGATTAAGTGTTATACATAATGATCTGCTTTTCAGGTTTGCTGAATTGATTTGATATAC
ATAAAACGACTGATATCATATTCTGGTAATATTTGATTACTTTGTTGCATGTAATTCCAGT
ATTTATTTATTTCTGCGTTTATATTCAATGTTTCTCCGTTTATATTCAATGTTTCTGCGTTTA
TATTAACACATTTCGTCGCTTACATCGAGCGGGCGCGCCGAGCCGTGACGCAAGGTAA
GCCTAGTACTATACATGTATACAGCATATATACGTATACGTTAGATCTACAATTTGAGTTT
TTCGAGTAAATGGTTATTCTAGCTTTATGATGTAGATATTTTCAGTTTCAAAACATTCCAT
TTACACCACTTCAAAAATTCAAACAAGCATATATTTAACTTTAGATTAGATAATCAGTCC
AATTTGATAGCGATATCAAAATGATGTTCGGATCAGTCTGGACTGAGATTTAGAAAGCA
TATGATGTAAATTTCAGTGGAATAAAAAAAAAGTATAATGTAACACTATCTTTTTTACGA
GTAAAATCCCAAAATTTAGCATGAATATATCTAGAATCCGTGTTTTTATTTCATTCAAACT
TCTGCTATAATGGTATGATGAAAACACGGCACGGTTTTTGAGTAAAAATAAAATGTGGA
CGGTTATCAGTTATGTGATATTGGAGTTACAGTACCGAACTTATACCGAAAATAATATGT
ATATCTATATTGTTGCCTTTGAAACTTTATCCATAACGTTTACTAAAAAGCAGAAATACAT
CAATGGTATTTCTGATATATGTTCCTATATTTAAGGTGTAGATTTAAATTCATTATTTCAAA
ATTATACTAAATCCTTAATAGTATATTCATTGTCGACCGTTTTGTATATCATACCGAGATTT
AATAGTATGATATATGAACATTTATCGTATAATCCAAAAAGTAACCACATACAGTGTTCA
CAGGCATTAAAAATAGATAGATATAATATCTTGTATCTTTTTTATATTTAGTGTGATAATGT
TGTAACCCTTCTTGTATGACTATCCATGTACATTAAGATTCGAAACGGTGAAAATACATG
CTTAGAATTTTTACTAATACCTTAGAAATTACTGGAGATTGCTATATGCCACGATTCCGTA
ATTACAGTCTAGACTGGAATAATTTTTAAGGTTAAACCTTTAGTTGAACTAGTTCTTAGA
ACCATTTTTGTTTCTGAATAAGACACCTTCCATTTTTGTTTTACCTGCATACGACATATAT
ATATATGTGCATGTAATATGTATTCCTAGGTATGATCAGGTATATTTTATTTCCATTTGCTTT
TCCATCTTCATTCTAAAAATGGAGGTGACAATAAAAAAAAACCTAGAGCATAGATGTACG
GGGTTTCCATAATTCAAATCACAATCCAATTAACGGATGTCCAAACCTGATTGACAGTA
AGACAATAGCAAATACCCGATATAGTCCACCGAATAGCAAATTGCCCGCGGCCAGGTAA
TTACAGGTGAGTTCGATGAATGGCGTTGTGTACAGGTAAATAACATCCTGGTCAAGGTA
TTACATAACCATCAAACCAAAGGCATGGCTAATTATATACATGTATCTATAATGTCGGTGT
ATCTACTAGTATATCGATTAAAAATTGAAAGCGACCGCACGGTCTAAAAAAATAGTTTG
TTTTGTTTAATATCTCAATATTTAGATCTTTTATAATATCGAAAGTTAATTCTTTCTACCAC
ATGTTGAAACGTATTACGGTATTGTAATAAATGTATCTCGATTTATTCGGTTAGCAACAC
ACAACACAATGTTTGTAATGATAAATCATCGGTGTGTACGTATGTCAAGCATCATATCCT
TGATGCTTTAATCAAGAATTTCTTCAATTGTTACGTAAAATTTCATTCTACAGTCACAAA
CTTTGCAATTCACAATGTATCAAAGATACAGACTACAGGAAAATATTATTTAATTAAAGC
GTATTCGTTGCTGAACTCCTCGACAAAAAATCGGAAACTTTTATTTCTGTGTGGATTTT
CTTTCATAATTACACGAAGATACCTGCTATTAGAGCACAAATTAGAGTATTTGAAACATC
GAATTTCACTCTTTTAGTTATTTATATTCGAGACAAAGTTATTGTACGATTAACTTCACTC
AAAAGTAATCATAAAAAAAAATCTTTTATCGGCTTTTCCTGTGACCGTCGATGTAAGTGAT
AGCACATCAGTTAAAGTACAGCAATAAGCCACGGACTATTGTGACGTCACACGGTTTA
GGGCTAGAAAATTTGACCTCGATATCGGCGGTTTACAGGTTATTCCGGTCGCGCGCGGC

ACGGAGAGGTTTCTACACGGTCTAATAAAGCCATTTCCAGCATAAACTGAAATTATCAT
TTTTGACTGCACAAATCCTTTAAATATATCATGGATGGATTTATAAATGCTTATAAATCCT
TGTATATATGTAATCGTGTTCCGTATTTTATTAACACTTTTTGAACTTGATTTAGGCCTAAT
AGTTATCGGACTAACGCACCATTTTATCATGATAAAAATGTAAATGTGTACAGATTACCG
AGTACCCGAATGTACTGTTACGTGAATCTCCTAAAATTGCTGACTTGCAATATGGCGTG
TGTGTCAATGATTATATATATAAAGTCAAGTGAATGTATTATATATGCCAATTCATAAATAC
TTGGTACTGAAACCACCCCCATTTGTATCCCAACATCGTCAAAAGTATCATCAAGGAT
TTATAAGTGAGAACTGTCTCTTTACCTTACTAAACACCACGCGTAACGCTAGACGAACC
GGGAAATCGAGTCCAACGAAACACCAATGTAAAGTTAATAAAATTTCACAACGTTTAG
TACTTGCTTTAAGGACGGAAGATTTAGAAGATATGTCTTAAATTTTTGTTAAAAAATACG
ACAACTCATGAAATGACGCTTCAGAAAGTAAGACGGTAACCCTCGCACCCACAAGCTA
CATCAAAGGCTGCGCCGGCGGATATCAACGGAAAATCAGTCGTCGCCCAACGTCACGG
TCAGTACACAAACGTAAAAGCTCCTACGTCGTACTTGCCCAAAACCCAGCGTGACTTT
TTCACCTCACTTAAGTCCTTGATATCATCAAGGATGCATTTTAGACAAAAATCCTTAGTA
TCATATTATCATCTTATTATAAGCGACCGCGGTGTGAAAAAGTGCTCACAGAAATTAGA
CATACACTACATATGAAACTATATATACTATGGTCTCATTAAATTATTAGGTAGCTGTATG
ACTGTGTTTCTGGTGTTAAAAATCATACACATATGATCGCATTGAAAAAGTGTATACAAC
CATCGTCGGCCATTTTTCTCACTTCGAGCTCCCCAAAATGCATACGCATCCCCTTCGTAA
CCCCGCAGGGGTACCGCTAGCTAGCTCCTACCCATGGTGTCAGGATGGCCCCAATAGG
GCAAGCAATATCCACGGCGTCCGTTATCTGTGGAGTTAATTAGTCGGATGACACGTGTG
CGGTACACAGTACACCTGAGAGGTGTATCTATGGCGACAACATATAAGTAAATGTACAG
GTAATATAATTCAACTGACAGAGAGAGAAGAACATATACTGGCAATAAGTTTTTTTTTT
TTTTTTTTTTTTTGTAAAGTTCATTCTTTGTAAACCCCCATTAAAGTGTACTGGGTTAAA
AAGGGTCTTCCCGAACTTCACTGACAATCTAGTCTCTGTCTTTCGTTGATTAGCATTTTG
AGTATATAATTTTTAACAAATGTATAGCACAGTGATCGTTCATATGATATTTTATGTTTAAC
ATAGTGACGTTGTGTATCGATTACGTCACGCACTAAATATAGCATTGAAAAAATTGCTAG
CCGTTGGTCGGTTTGTGTTGACACAATATTCCATGTTAATGTGTATTTTATGGTGTAATAA
ATAACATGCCAGCTATGATAACTCCCAGTAACTGGGTTTCGAAAGGTGTGCTCTTAAAC
TGAAAATTGTATGAAAATTCTGTTATTATGTGACCTTCTTATTAGTGCTTTATTCGATTCG
TGCCATGACGGTTCATCAGGATATTTATTCAAACCAATATTATTTTCACTTTTGTGTCTCT
TTTTATAAAAATAAGATATGATGATTAACACAGACACCGGTATATCCCGATGTCATTTCCT
ATAACGAGTTGTACATAGATCTAACAAATATCCCCAGCTAACCAGTTGCTTTTAAAGAC
GTTTTTAATATTGTCCAGTGATAGTAATATTAGGAAAAAAAAACCTTTAAAGATATTTTT
AGGTGTAACGACGTCACGAGTATAAAAAATGCATACTGAATCAGAAACGTATAAAAAA
ATAATGTATGTCGTTAAGTTTCAGATTCAATAAGTTAATCGACAAAGAAATAATAATTTT
AAAAAATTCGTTGAAACCTCACTACCATTATGTGTAAACTGGAATATGAAGAAATATAC
ACGTTTTATCATTTAACCAATCGATGAATACAGCTCCCTATACTACTCTCCTTATGGTCGT
ATCCAGAGTACCTCTCTTTGACGACACAAAATAAATCATACATGACCAAGCTAAGTTCG
CTTCGTTGTAATAACATAGGCCTGGGCATGGCCTCGTAATTAGGGTAGTAGTAAAAACC
TTTACCATAACACTGACTAATTAGCAAAGACAGTATATCACAATTGTTTATTTAAGTCAA
ACATGTAATTATGTAATATCAAATAAAAAAAAAACAATTGTGAAAAAGAGTGATCTTTT
TAATGACCAATTAAACTTCTTAATTGCCGGAATCTTGTTTTGATTTTATAGTCTTTGTCAT
TCCGACAAAAAATATCAGTGGCCAAGGCTATAAATACTCCCTCACACAATCCTTATAGTA
TCATACAACATGAATACCTTTCAAGAAGCAACCTACCGATACCTGTCCAGCGACACCA

AGAAGACCTGCATCCTCGGTCACAGCTTTATCAGACGCCTCATCGCCCGATGGAGAAA
AGAAGAAATACCACTACCAAATTGGTCATTCCATGCCTTCGGACGAGGTGGTTTGAAG
CTTCGACAGCTGAGAGACATCGTACTAGCCGAAGATTTTTCCATCTATCACACCGTCTT
TGTACAGATAGGCGAAAATGACATCGCTGACCAGTCAACGGCCCGTACAATTGGACTT
CTTCAAGAAATCCATCAACTGTTTATCGCCCAGGGTGTAGAAGTGGTGATGTTCGGCG
AGCTTTTTCCGAGACACGATCGCAAATTCAACAAGACCGCATCACTTATCAACAGAAT
GATGAAGAAACAACACCCTGACATCATTTGGCAACGCGACAGAGACTTGTTGATGGTA
CACACCTGAAACCGTCGGCGGAAGTTGCTTTCTCCAACAGCATTGACCGGGCCATCAG
ACACATGAGAGCTTGATCTTATAATCAGCCAATCCTTTATAAAAAAAAAGCCATTTTCAT
GGCTACCCAAATATCACAAAAAGACCCACGAACACTCGAAAATACGCATTTATATATTA
CGAATTATATATCTTTAGCATGAATAAAAACAACTGAAAATTGACACGTGTATACTATTT
ATAATTTTATTTTAAATAATTGACGAATACTTCTTAAAACTAAACATGTATTTAATCAGAA
ACATATATGTATAATTGTTTCTGACAATAGGAATTAAATTTTATCATATATCGTACTAATCG
TATTAGTTCACCAGTTAAGTTTTGGCCTCTATACTGGCAACAAAGTACATCAAATAATTA
CCAGAATATGATATCGGTCGTTTAATGTATATCAAATCAATTCAGCAAACCTGAAAAGCA
GATCATTATGTATAACACTTAATCAGCATTTTACAATTAAAGGCAACAGCAAAACGTCT
GTAAATAACAAATATTTATTCATTTTATGGAAAACGATCTTATTTTAATATCGTCAACAAT
TCAAATTTGTGAGAAGAATATACATACATGTAATTGCACATAATCAAACACATAATTTGT
TTGTCATAGTTAATCCCCGTGTACATTTGTTACCGCTCATATCGGAACTCTTCCGAAAAA
AGATGCAAAATGCATAGCTCAAATGACGAGACGCGGGAAAGAAAACAACAAAATATA
CATGTAGGAGAATAGGATAATTAGGGTATATGTATTTTTAAAGGGGAAAAGAGAATTGT
AAACATAATTATTTTACATGTACAATCGGCTGGCGGCTGTATGTACTGTAAGTCTCGTTT
CACAAGGTAAAGTTTACCCGTGTAAGCCGTGTGCTCGGCCGTCACGGCTCGGCGCGCC
CGCTCGATGTAAGCGACGAAATGTGTTAATATTAGCGCAGAAACATTGAATATAAACGC
AGAAACATTGAATATAAACGAAGAAATAAATAAATACTGGAATTACGTGCCACAAAGTA
CATCAAATATTACCAGAATATGATATCGGTCGTTTAATGTATATCAAATCAATTCAGCAAA
CCTGAAAAGCAGATCATTACATGTATGTATAACACTTAATCAGCATTTTACAATTAAAGG
CAACAGCAAAATATCTTTCAAAACGTCTGTAAATAACAAATATTTATTTATTTTATGGAA
AACGATCTTATTTTAATATCGTCAACAATTCAAATTTGTGAGAAGAATATACATACATGT
AATTGCACATAATCAAACACATAATTTGTTTGTCATAGTTAATCCCCGTGTATATACACAT
ATTTTACACATAACCTAGACAGTTATTCATTTGTTACCGCTCGTATCGGAACTCTTCCGA
AAAAAGGTGCAAAATGCATAGCTCAAATGACGAGACGCGGGAAAGTAAACAACAAAA
GATAGGAGAATAGGATAATGAGGATATATGTATTTTTAAAGGGGAAAAGAGAATTGTAA
ACATAATTATTTTACATGTACAATCGGCTGGCGGCTGTACGTACTGTAAGTCTAACTTTA
CAAGGTAAAGTTTACCTGTGTAAGCCGTGTGCTCGGCCGTCACGGCTCGGCGCGCCCG
CTCGATGTAAGCGACGAAATGTGTTAATATTAGCGCAGAAACATTGAATATAAACGCAG
AAACACTGAATATAAACGCAGAAACTCTTAATTCTATCGCCGAATCTGTTAATTTCACC
GTCGCATCGGAAAAGTTTGACGATTCCCAACCTCCGACAATGTAGCAATAAATAGCCGT
GCACGTAACGAAGTTGGGTAATTGTGACTGATACCCTCTGTTATTCGGCCGTCAGCCGT
CACGACCCACGGCGGACAGAAAAATACTTAATTTTACGGCAGAAATACTTAATTTTATT
AATCGAATTGGAATTCTGAGGAAAAGTCTTAATATAAAGGGTTAATACTGAATAAAAAA
GCACATATGCTAATCCTGCCGAAACTTGTTAAAAATTAAGTATTCTGCCGTTATTCTAAC
AAGTTTCGGCAAGATTAACACATTTTGGGTTACCAGTGAGGGTGGAGCTGCGGTCTGG
GGTAACAGGTTCAGCTGGCAGCACACTACTCTCTTCCTGTTAGCCAGAGATGCTCTGG

TCATGACACTAATTAATATTTATCTGGTGTAGGGCCAGAGCGCACTGCTATATCAAAAGG
TGGAACTTGCCAACACCGTTTGGTATCTCCCTGTTACATAACTGATAAGTACTTAAAAA
ATTGTACTTTCTAAAGCAGAAATATATGACAATTCAAATTATATAACTGGATGAAAAGAT
GAAATGATTGAATACATGTGTGTGGTTTTTTAGATCATATACCACTAGTATCATGTTTCTA
ACTAATGATAAAGATTTTCTTTCCTCTACAGATTTATGAAATTACATGATGCCCAAATCTA
AGGAATTCATTTCATCATCAGAGTCTGATGTGGACTCTGACGAGGTAATTACATTTCTTT
TCTTTTCCATGGACTCTGACGAGGTATACACAATTACATTTGTTTCCTTTTCCATGTCTA
ACGCTTCAGGTTATCTGTGCATGAACTTATTAAGCTTGAGTCCATTCCATTGATAACTAG
ATCAGATAATTAAGCCGTTCATAAAATAGTATAGTGTCTTATATATATATATTAATAAGTGT
CAAGGCTAGAGCATCTCTGGCTAACAGGAAGAGTACAGTATGCTGCCAGATAGTGTCT
GACAAAGTTTTAAAGACACTTAGCTCATCTAATCCTAGTACAGTATGCTGCCAGATAGT
GTCTGACAAAGTTTTAAAGACGCCTAGCTCATCTAATCCTAGTACAGTATGCTGCCAGA
TAGTGTCTGACAAAGTTTTAAAGACATCTAGCTCTTCTAATTCTAGTACAGTATGCTGCC
AGATAGTGTCTGACAAAGTTTTAAAGACACTTAGCTCATCTAATCCTAGTACAGTATGC
TGCCAGATAGTGTCTGACAAAGTTTTAAAGACGCCTAGCTCATCTAATCCTAGTACAGT
ATGCTGCCAGATAGTGTCTGACAAAGTTTTAAAGACACCTAGCTCTTCTAATCATAGTA
AATGCCTTAAAAAATTTTAAAATGCTTTAGAATAAGTTTTTGCTGTTGGGTCCATTTAAG
ATATTTCTCTGGAATTCATTTTTATCTCACCTGGCCCGAAGGGCCGGTGAGCTTATGTCA
TGGCGCGGCGTCCGTCCGTCCGTCCGTCAACATTTCCTTTAAATCGCTACTAGTCTGCA
TGGATTGTAACCAAATTTGGCCACAAACATCCTTGGGGGAAGGGGAACAGAACTTGTA
TAAATTTTGGCTCTGACCCCCGGGGGACAGGAGGGGCGGGGCCCAATAGGGGAAATA
GAGGTAAATTCTTTAAATCGCTACTAGTCATAGAGTTTTGAATGGAATGTAACCAAATTT
GGCCACAAACATTCTTGGGGGAAGCTTCTTATGCAACATTGGCTGTAGTTAGCAGGATA
AGGAAACACATACATATATACCCTGATAACCAATGATGATTTACCGGTTTCTATTACATAT
ATATTTTCTAGTCATTTAATTTCAAAATAAAAAGCCTATGCAAATCCTGCCATTGTCCAG
AAATGACAAAATAAATTATTTTTATAAATAAATATAATAAAGGTGATGATGGGGGGGGGG
GGGGGAGAAATGCTTATAACTTAATATAATAGAACTGAGGGAAAAATAAATATGTAAAA
TAAGTATGTAATAGAATACCCAAGCGGGTCAAAAAAGAGCAATATAAACAAAATAAAA
ATACAAGCAGGATAAATAGTATACAGTAATTAACAATAGTTCAAATAACAAATATGTAAT
AGAATACCTAAGCGGGTCACAGAAGAGTAATGTATATGAAAGAAAATACTAGTCATGTG
AGGAAGGGCTCATAGTATTTGAAATAAAACAATTGTTTATAATCCACTAAATTTTCATTG
CTTTATAACATACTTAACATAATAATTATGATAAATTCTCTATTAAGTAGATACTTTGATTT
CATTTACATAATTGATTTTCACTGACTAATTCCTGATTTTAATACCCAATAGAGAGATTAT
TTTATTCTTTAATATTTCAGCCAAAGCCCAAAAAGAAGAAAAGAGAACAAGTGCCTGA
AAGCAAGAAAGAAAAGCCTGAAGCTTCTCAAAGTAAAAAGCCAACAAAGGGAGCAA
ATGGAGAGCAAATGTTTCAGGTTCATTTAGTTTGACTTTTATAATAGATTTCTATTTTTTT
CGTCCTTTTGAAACGTTGATAGGAATATTTAACTTTGAAGTTAAACCTTATTTATCTTAA
CATTTTAGTGCTCTTATGTTTTAGAAATGACCCTTTTGAGTTTTGAAAGAAATGATGCCC
ACTTATAATTATCTGCATACATTATTTACAAGTTCTTCAATTATTTATTTGATTATTTCTTG
AATATTTACTACAAATCTGAGAGCTGTAACAATTCTTTGTTATACATACACTGTATATATA
TTCACAATAGTTTTCTTACAATGCAATATTTGATAGATCAATGTCGTATTGATAAGATACA
ACACTGTATTTTGTCAATAGCTTGAAATATGGTATTAAAGTATATTGGTTCTTGAAAAGA
ATGTCACAAAATCTTATTGGTATTGTGTCTTAAAAGAAAGTGCACAAACCAAAAAGAA
ATGGCAATTACAGCTTTATTCACCAACTTAAAATCCCATTTAATGGATCGAGTTGATGTT

TACAGGGAATATTAACTTGTACATTATGTGTTCCTTATCGAGTTGATGTTTACAGTGAATA
TTAACTTGAACATTATGTGTTCCTTATCAATGTTTACAGTGAATATTAACTTGTACATTAT
GTGTTCCTTATCGAGTTGATGTTTACAGGGAATATTAACTTGTACATTATGTCTTCCTTAT
CAATGTTTTCTGTGAATATTAACTTGTACATTATGTGTTCCTTATCAATGTTTACAGTGAA
TATAAACTTGTACATTATGTGTTCTGTATCGAGTTGATGTTTACAGGGAATATTAACTTGT
ACATTATGTGTTCCTTATCAATGTTTACTGTGAATATTAACTTGTACATTATGTGTTCCTTA
TCGAGTTGATGTTTACAGGGAATATTAACTTGTACATTATGTGTTCCTTATCAATGTTTAC
AGTGAGTATTAACTTGTACATTATGTGTTCCGTATCGAGTTGATGTTTACAGGGAATATT
AACTTGTACATTATGTGTTCCTTATCGAGTTGATGTTTACAGGGAATATTAACTTGTACAT
TATGTGTTCCTTATCATTGTTTACAGTGAGTATTAACTTGTACATTATGTGTTCCTTATCG
AGGTGATGTTTACAGGGAATATTAACTTGTACATTATGTCTTCCTTATCAATGTTTACAGT
GAATATTAACTTGTACATTTTGTGTTCCTTATCAATGTTTACTGTGAATATTAACTTGAAC
ATTATGTGTTCCTTATCAATGTTTACAGTGAATATTAACTTGTACATTATGTGTTCCTTATC
AATGTTTACAGTGAATATTAACTTATACATTATGTGTTCCTTATCAATGTTTACAGTGAAT
ATTAACTTGTACATTATGTGTTGCTTATCGAGTTGATGTTTACAGGGAATATTAACTTGTA
CATTATGTCTTCCTTATCAATGTTTACTGTGAATATTAACTTGTACATTATGTGTTCCTTAT
CGAGTTGATGTTTACAGTGAATATTAACTTGAACATTATGTGTTCCTTATCAATGTTTAC
AGTGAGTATTAACTTGTACATTATGTGTTCTGTATCAAGTTGATGTTTACAGGGAATATT
AACTTGTACATTATGTGTTCCTTATCGAGTTGATGTTTACAGTGAATATTAACTTGTACAT
TTTGTGTTCCTTATCAATGTTTACAGTGAGTATTAACTTGTACATTATGTGTTCCTTATCA
ATGTTTACAGTGAATATTAACTTGTACATTATGTGTTCCTTATCAATGTTTACAGTGAATA
TTAACTTGTACATTATGTGTTCCTTATCATTGTTTACAGTGAGTATTAACTTGTACATTAT
GTGTTCCTTATCGAGTTGATGTTTACAGGGAATATAAACTTGTACATTATGTGTTCCTTAT
CAAGTTGATGTTTACAGGGAATATTAACTTGTACATTATGTGTTCCTTATCAATGTTTACA
GTGAGTATTAACTTGTACATTATGTGTTCCGTATCGAGTTGATGTTTACAGGGAATATTA
ACTTGTACATTATGTGTTCCGTATCGAGTTGATGTTTACAGGGAATATTAACTTTTACATT
ATGTGTTCCTTATCAATGTTTACAGTGAATATTAACTTGATGTGTGTTTAGATGTAGATTC
ACAGACTAACTTCTGTAATGGATTATTTTACAGCTTTCCAAAATGCGGTTTGTCAGTGTC
AGTGAATTCCGAGGCAAAGTTTTGGTGGGAATAAGAGAGTACTATGAGGCTGATGGAG
ATCTCCGACCAGGCAAAAAAGGTTAGGGCTGTATAGGCTTTCTTAACTTGGTACATGTT
TAACCTGATGAGTGACCTCCGACCAGGCAAAAAAGGTTAGGGCTTTAAAGGCTTTCTT
AACTTGGTACATGTTTAACCCGCAGAACAACCTCCGACCAGGCAAAACAGGTTAGGGC
TTTATAGGCTTTGTTAACTTGGTACATGTTTAACCCGATAAGTGACCTCCGACCAGTCA
GAAAAGGTTAGGGCTTTATAGGCTTTCTTAACTTGGTACATGTTTAACCCAATAAGTGA
CCTCCGACCAGTCAGAAAAGGTTAGGGCTTTAAAGGCTTTCTTAACGTAGTACATGTTT
AACCCGATGAGCGACCTCCGACCAGTCAGAAAAGGTTAGGGCTTTATAGGCTTTCTTA
ACTTGGTACATGTTTAACCCAATAAGCGACCTCCGACCAGTCAGAAAAGGTTAGGACT
TTATAGGCTTTCTTAACTTGGTACATGTTTAACCCGATAAGCGATCTCCGACCAGGCAA
AAAAGGTTAGGGCTTTATAGGCTTTCTTAACTTAGTACATGTTTAACCCAATATGCAACC
TCCGACTAAGCAAAAAAGGTTAGGACTTTATAGGCTTTCTTAACTTGGTACATGTTTAA
CCCGATGAGCGACCTCCGACCAGTCAGGAAAGGTTAGGGCTTTATAGGCTTTCTTAAC
TTGGTACATGTTTAACCCAATAAGCGACCTCCGACCAGGCAAAGGGCTTTATAGGCTTT
CTTAACTTGGTACATGTTTAACCCAATAAGTGACCTCCGACCAGGAAAAAAAGTTTAA
GGCTTTAAAGGCTTTCTTAACTTGGTACATGTTTAACCCGATAAGCGACCTCCGACCAG

GCAAAAAAGGTTAGGGCTTTATAGGCTATCTTAACTTGGTACATGTTTATGTGATGAGTG
ACCTCCGACTAGGAAAAAAACGTAGTACATGTTTGATCCAATAAGCTTCGTGTATATTC
CCTCTGCTCTAAACCTCAACTTCAATTTCTCCCAAGCGTTTTCACTATATATTTTAACTCT
ATTAAATGACCATCTCTCTAATGCGGCCAGGGACCACTTAACTTCCTTCCTGTGGTTGCT
AAAAATCCCTTTACTGCGACCAGAAAAATGAATGTCTTTTCGATCTTTCCAGAATGCTT
GTCATCAGGTCGCCATTTTGACACGGCTTGGACAGAGCAATTAAGCTTATAGCTTGTTA
ATGTAAACAAAACAGTGTGAAGCAGTTAGCCTGTGATTGAAACCCTATATAAATAATGC
TACACTGGCCATGTGATAATTGTATCTGACTGGTCTTTTTACACCTTTAATTTGTTACCAT
CAGACCCCGGGGCCTTTAGACGGAATGGGTCAAGCTCAATGGCAGTCTCCATACATTTT
AAAGTATATTATGGTTAAATAATGAACTGTTTGATACAAAAATGATGAACATTGTTTAAA
AGAAACACTAATAGTGACTGACAATAGGAGATTTCAGAAAAGATGGCGATGACGTCAC
ACAAAGGTACATCCGGGCGCTCAAAGGTACAACTCTGTATAGCTGTACACATTAACATG
TGGGAATACCGCCGCTTGCGATGTTTGTTTACATTTTTAGCCCACCATCATCAGATGGTG
GGCTATTCAAATCGCCCTGCGTCCATGGTCCGTGGTCCGTGGTCCGTCGTCCGTCCGTA
AACAATGCTTGTTATCACTATTTCTTAAAAACTGCTGCAGGGATTTTGTTCAAACTTCAC
ATGGAGGTTACCCTTGGTCCCCAGTTGTGCCATACAGATTTTGAGGCTGATCGGAAAA
ACAAGATGGCCGCCAGGCAGCCATCTTTGATTTTGGCAGTTGAAGTTTGTTATCGATAT
TTCTTGAGAACAACAGAAGGGATTTTGTTCAAACTTCACATGGAGGTTACCCTTGGTC
CCTAGTTGTGCAAAACAAGATGGCCGCCAGGCAGCCATCTTTGATTTTAGCAGTTGAA
GTTTGTTATCAATATTTCTTGAGAACTACTGAAGGAATTTTGTTCAAACTTCACATGGAG
GTTACCCTTGGTCCCTAGTTGTGCCATACAGATTTTGAGGCTGATCGGAAAAACAAGAT
GGCCGCCAGGCAGCCATCTTTGATTTTGGCAGTTGAAGTTTGTTATCGATATTTCTTGA
GAACTACTTAAGGAATTTTGTTCAAACTTCACATGGAGGTTACCCTTGGTCCCTAGTTG
TGCCATACAGATTTTGAGGCTGATCGAAAAAATAAGATGGCCGCCAGGCAGCCATCTTT
GATTTTGGCAGTTGAAGTTTGTTATCGATATTTCTTGAGAACTACTGAAGGGATTTTGTT
CAAACTTCACATGGAGGTTACCCTTGGTCCCTAGTTGTGCCATACAGATTTTGAGGGTG
ATCGGAAAAACAAGATGGCCGCCAGGCAGCCATCTTTGATTTTGGCAGTTGAAGTTTG
TTATCGATATTTCTTGAGAACTACTGAAGGAATTTTGTTCAAACTTCACATGGAGGTTAC
CCTTGGTCCCTAGTTGTGCCATACAGATTTTGAGGCTGATCGGAAAAACAAGATAGCCG
CCAGGCAGCCATCTTTGATTTTGGCAGTTGAAGTTTGTTATCGATATTTCTTGAGAACTA
CTCAAGGAATTTTGTTCAAACTTCACATGGAGGGTACCCTTGGTCCCTAGTTGTGCCAT
ACAGATTTTTAGGCTGATAGGAAAAACAAGATGGCCACCAGGCAGCCATCTTTGATTTT
GGCAGTTGAAGTTTGTTATCTATATTTCTTGAGAACCATTGAAGGAATTTTGTTCAAACT
TCACATGGAGGTTACCCTTGGTCCCTATTTGTGCCATACAGATTTTTAGGCTGATAGGAA
AAACAAGATGGCCACCAGGCGGCCATCTTGGATTTTGATAGTTAAGGTTTGATATCCCC
ATTTCTCAACAAGAGGCCAATGGCCTCGTAGCACGATGGTCCAGTTAATTAATGTTCAA
TGATTGTTTTTGACTAATATTCTTGATTATCACTCATATGCACTGTTTAAACTAATAATTAA
TTAGGACAATTCAAATGACCACTGGTATTTAATTGTTATCAGTGTTGGTATTAAAACAAT
CATAATATTCTTTCAAGACAGATGTCATTCATCAAATGTTAAGTTATATATAGATCAACAG
TTTTATAAAAAAAAATTGATTTGTGCAGAACAGTTGTTTTTTTCTGAGCAGGATTTTAGT
GTGAGCGTATGCAGAAGCAGCATCTTCTAATTAAGAATATTGTTGGGACCAATTCAAAT
GGCATCAACTTACCTTTGATATAAGAAAATTTAATAAAATTCAAAATTTAACAATGCTGT
TTATCCAAATTTATCAGAATATTGTTATATGATAAAAATAAATGGGGAAACTTTTTCCTGT
ATGATTGCTTATCGGTATCATACATGTACAACTATGTATAATTAAAGTGTTAAGTTGACTA

GACTTTTAAAAGATACCTGCTGGAGGAGGGCTTAATTCATTATAGGTGACAAAAAAAA
AATCAAAGATGTTTGGCTGTCCTGGTTATAAGAGGTGAACTGAATTTGCTTCAATACCT
TGAAATTAATTACTTATCTTTTATCTCATTTTCTTCAACACAATATAAATTTCACAATTAA
GATAACAATGCACAAGTTGTATTGGCTGGGTCTCGGCACAACGGAATGTTACTACGGG
TCAACAAATTGACATTGGCAGCTACAGTCGAGCCTTTCCCAGGGCCTTGATAACAAAA
CACATTAATTAACTCTCAACCATTGTTTTTCCCTTTAACAGTAAATGGTATATGTAACACA
TTTACACACACATATAGGCCTATAAACAGCATCTAGATGAAACTAGGGCACCTGACTTG
TGACACTGTCCAGGGAATATAGATTATCCAACACAGATAATTATGATGTCATGACACTTG
AGTAACATATTTAAGAAAAGAAAAATAATGAATATTTCTTAATTGCCTTAATCTTTTCTTA
AACTATATTTCTCTTGTATGAAGTGGTACCGTCGGCCATATAATTTCCCATAATGCATTGT
GTTTCTCATTTAAATATTACTATAGACAAAGAAGGAATGAAATATATTTTAATTCCTCAA
AAATACAGTTTATGTCTGTTTGTCGCCAAGTTTTATCAGTTCCGTCCTTATCTATAACTAC
ATGTCTGTGCCAAATTTCATTGCTGTTATTGGTGTATAACTTGAGATATCCTGTTCACTAG
GTGATTTTCAAAACTAGGGGGTATGCTATAAGCTTCGATGGAGCTATGCTCCATCGTGCT
AAAAAGTGCTGAAGGGATCTTTCTCAAATTTAATATGTAGGTTCACCTAGGGACCTAGT
TGTGCATTTTTGGACCAATCGGTGAACAAGATGGCCGCCAGGCCGCCATCTTGGATTTC
GATAGTTAAAGTTTGTTATCGCTATTTCTCTGAAAGTACTGAAGGGATCTGTCTCAAAAT
TCATATGTATGTTCCCCTAGGGCCCTAGTTGTGCGTATTGTGATTTGGGACAGATTGATC
AACAAGATGGCCGCCAAGGAGTCATCTTGGATTTTGATAGTTGAAGTTTGTTACCGCTA
TTTCTCAAAAGGTACTGAAGCAATCTGTCTCAAATTTTATATGTAGTATGTTTGAAAAAG
TTTAAAAAGTAGAGAAAAGATCCATCTTTCCTTTGTCAGATATAGATCATTCTTTGGTGG
GCGCCAAGATCCCTTTGGGATCTCTTGTATTGTAATAAATTGTACGACAATCCAAGAAA
TGTTGCTTTATTTTCATTTCAAAAAGTGTAAATGAAAGTATGTAACAGTAATATATAGATA
TAAACATATGATATTCATACTTTGTTTAGACTCCATTTTCGACTGCCCGCGAGAAAAACA
TGGTCGCCATTACCGTTTAAACATTGACAGAATATTGCAAATATTGGCTTGAAATTACAG
ATTAAATTTCAAGTTCCCCAACATATCATACTGTCATTTTATAAAGCGATTACTGTGTTGT
AAGTCTTACATGGTAAAACACCAAATCACGATTTGTGATTATGACAAAATTAAAATTTG
TTTTCATAGATTACCCCAAGCGCACGGCAGCCATATTAAGTACTGCCGACATCCTGAATT
TCGACCTTTTTCAGAGTAACCAAATTAAGTATTCTTGATAACTTTTTCGTCAAAAATTTT
TGAATTGAGTTTATGATATGCAAATGAGTCAATTTATAGTTACTTTTTATGATATTTTTAA
ACAAAACTTTAAGTAATTTTAGGATTCTTGAAGCCTCGTGCAATGTGTCCTGGTAGGCA
TTTCAGTACTAAGATCTATATTTATAAATCTAAATGTAGTCAATCTAACAGGTATTCAAAA
TATTTTTAAGTAATATCACTTCAATTTAAGACTTCATATAGCAACCCATGGAATGAAAAC
GACTGAAAATTAAACAATAAACTCTACAGCATGAAAACTGAGTGATTTCACCATTTTAT
TTTTCGAGATATATTGGCAGCCATACGTGTACGTACACATGCATGTAGATCTGCATGTAC
GTGTAACACAGTGGTTTAAAGCATTCCTTTAAAACAAGATATGCATGGTAAAGAACGA
AAAACACAAGTACTTATTGAGATATGTGTTGGTAATTACATATGTATTAATATTATTAATT
TCCCCAAGTATAGGCTGTGGCCTGAGATGATCTACCAGCGAAGCCATGCACGAGCGGC
CGTGGTCTGGCCAGATATGGTCCACATTCAGTTATATTAAATAGTGTTGTGAGCAGCTTT
TCATGACAGAAACATGACACAAAAAAATCAATTATCTATTCATAACTTTTTCAAAACAT
GGCTTAAGATAAGGTCTACATGTGAACAAAAAACGGGTACATGTGACGGCCCGGCACC
GCTTCGCAAGCTGGCTTCAACACTAAATCTACAGAAATATATTTAGCAATAAATACTGTA
AATATAAAAATATAAATGTCCCTAACCTCTGAAATAATAAGAAACCATTTTAAAATTAGA
ATTTGTAAGTATGAAAGTGTATACTTTTGTCACAGTATCGATTGTGTAGCAGGGATATAC

ATATCTGTTATTGTTTTTATTAGTCGCCATACTGTTTGTACCTTTGAGAACCCAGATCTAA
ACTTTCTATGACGTCACGCCATCTTTTCTGAAATCTCCTATAATTTACTTACATGAAATTG
AAATATGAAAGCAGTCGAACAGACATGCAGCATTTTTTTAGGCTGCCATCTTAGATTT
GTAATAGTAATAGACAGAACAAGAAATAGGTATGAACAATCCGAATTTAATCGGATCTA
AATGAAAAAAATTGGAAAATAAATATATATAAGAAATCAAATAATGCCCAACCTGTGAT
CTTATTGCCACACAAATATATTGTTTGCAATAAAACTTGCTTAGAACAGTATTATTACATG
AATAAATTACTGAAATGATTTTTAGCACGATTAGCATACCCCCTAGTTTTGAAAATCACC
TTGTGAACAGGATATCTCAAGTTATACACCATTAACAGCAACGAAATTTGACACAGACA
TGTAGTTATAGATAAGGACGGAACTGATAAAACTTGGCGACAAACAGACATAAACTGT
ATTTTTGAGGAATTAAAATATATTTCATTCCTTCTTTGTCTATAGTAATATTTAAATGAGA
AACACAATGCATTATGGGAAATTATATGGCTGACGGTACCACTTCATACAAAAGAAATAT
AGTTTAAGAAAAGATTAAGGCAATTAAGAAATATTCATTATTTTTCTATTCTTAAATATGT
TACTCAAAGTGTCATGACATCATAATTACCTGTGTTGGATAATCTATATTCCCTGGACAG
TGTCACAAGTCAGGTGCCCTAGTTTCATCTAGATGCTGTTTATAGGCCTATATGTGTGTG
TAAATGTGTTACATATACCATGTACTGTTAAAGGGAAAAACAATGGTTGATATCAATTAA
AGTGAACAGTCGGGCAAGGATATAGCTAAAGATGGCTAAAGTCGGTAAAAATGGTGTG
AATGTATCCAGTATAATTTCTTATGAAATAGAAAAATAAAATCTCTCGTCAAAATCTAGC
TGTCTGCGTACGAAGAAATTAATTGAAAGTATGGGAATTCTAAAACATCCTCCCGGCTC
ACTATGTCCGTGGTTACATTTCCACGCAGCCTCACTTTCAATGCTTTCAACTTTTCTTTA
CTTTAATGGTCAGAACTGGGAAACTAAGCAGAACTTGACCGTTGTTATTCGCCGGCCAT
GCCGGCGAATCTATTTGTAATAAGTTTGAAAACGAACCATGGGGATGGGGATAAGTCTG
TTTGTTGAATGGTATTAAACGGATTTATTGTTAAACAAAAGTTACAACAAAAATACTTTT
CGTGTTTTCAATCGTAATTGCAGGATTATTATAAACATATTTAAAAAAAATTGGGGCGAT
TTGTTGTCTAAATATTCAAATATGTCCCTTCCTTAAATTAAGGAATGTACAATAAGTGCT
CCCCGGCTAGCTTACAATTTACAAAGGCTGCTATTATGTCCACCGCTGCAAATATAGGTC
TCACTGTGTGAGTCATACCAAAATTGCTGTGTGTCGTTCTAAAAATAGATCCTACCTGA
GCATGGCAGCGAAGCGTGTGTGCGGTGGAACCAAATAGCTACATGTAGCTATATGTCG
GCTAAAGGCAAGTCAGCAGATATTCGGAATGCGTTGGGATTAATATTTGCTTTGCATATA
TAAACCTGAAGCTGTTCCGAGGTTTCCCGTGGTTTTGTCAACTCAGACAAAGAAAACG
GTTGTTAGTATTTTACGGAATCGTAATTGAAAAAGATACAGAACGTTTGTATAATATTGT
GAAAATAATTAATTCTAACGACGTGATAAATATAAATTTAAATAATAGTTACAGTATTTAT
AAATTTGGATCATGATTTGTATAAAAATGTTATTTTTATGTTTATAAATTAGTTCAGTGCG
TTCCGTACAAACGAGTATTTTATATACAACCTTTTCCTGACTGACAGACCATGAGACGT
CGACCGAACTACGTAGAAATCTACTCTCAAGCTGATCAACACTTCAAAATGTTGAATAT
TGGTCACCAGCAATTAAACTAGTAATCATTACGTATAACCGAAAAACAGTAAAGTTTAT
ATATACATGTATATCTCATTGACCTATCCATTGCAAAATTCCTGCCAAAAATAAGTGCCA
AAATTTGCATATGTTTTAATTAAGGTACCTGAGGGATCAATTGTTAAATAAAAAAAAATT
AGGACAATTTTTCCAAAATGTTAATTTTAATTGTTTTGTTATGTATACGAAGTAACGTTTT
TTCCTTTGTAGGTCTATTTGGACTGAATTTTTTTAAAGTGCGTAATGTTTTACTTTATTGT
CCTCTGTGTATTTTGCCTTTGTACAATTTTGTATTGTGACCAGATGGTTCGTATATACAGT
AAAACCCCTTTATATCGCCACCTTCTGTTTTCCTAGATTTTGGCGATATATCGAGTTTGG
CGGTATATCGGATTTGTCGTTGAAATCTTAATTAGGCCTAAATATAGGTAGTCCCCTGTG
CCGTACTGAAACAGACTTTCTAGTCAGTCTGTGTAGTGTACGGCCACTTCGTATTTTGT
AATATGAACAGTTCCAAAATTAAAGTAATTTCGATAGTAGTCTTTCAATAAATATGTCAG

CTTTATGTGTGTAAAATACGAAAACCGAACACGTTTGTGCTACCACGTATCGTGCCGTA
GTTGCCTGCTAGTTGGGATTTTCTCGGTTACATGATATTTTTATTACGGGAAATCCCGCC
CGGAAACAAAGAATACAAGTTCTGAACAAGCGCAGTGCGGTCGTGACCTCACTTCCG
CGCACATGTCTGCATAAACGGCACTCAGACGACGATTTGATCTTACTAATAAACGGCAC
CTTTGTTATTGTTTTACAGGTAGGTATATATCGCAAGTTGTGGTGAATAAAACTCACTTC
TGTACATGTGTTTTACGTACTATCGTTACGTTAGTTATTTATTGACAAAAAGGAACCCAC
AAGTCTATTTAGAACTCTATATCACCGTTCGTTTATAGACCATAAAATGCAACTAGCAAT
TTCAAGAGCGATAGAAATATAACGTACGTGCAGAGGAATCCGTGTACCCATATTGTTAA
TTGATTTCACTGTGTACACACGGTCATACACGCGGCCATAGTGTGTGTAGCTGATTACC
TGTCAGTCGGCAGTTGAGCACCATACGAAAACATGTGTCCCTATCAACAGAATTTGGC
GATATTAACGAGGTCATTAATAGTGTTATTTTAATCATTTGTTCCGCAAATGTGATGGCG
GATGGCGGTAGACGGGTTTGGCGATATAACGGGTGTATTTGTATGGAGGGAAATCCGTT
CTGAATAAATTCATGGCGATAAGCGAGTTTGGCGGTAAAACGGGTGGCAATATAACGG
GGTTTTACTGTATAGATGAGTACATCAACATGAAATATATATATATATGCTACTACTGGCA
ATGGCATAATTACATTCCATATACTTTTACAATAAATATTGTTGATAATTAGAAATAATGCC
TCCACAGAAAAATTCAAATTCCAAGTAATAGTCATGTTTTCAGCATTAACTATACTTAAC
CAGATCCATGGCCATTAACTGATTCCATTTTAAATGTTACTGTTCTTAGATGTATATATTTT
TTTACTTGTTAAACATCAACAAACTTTGCAAAGTTCAACATGTTTTCTTTCATCTATTTG
TCATTCTGTACCATTTAACATGTGATCACATATTTGATTTCATTCTATGTGATCACATGTTT
CATTTCATTGTATGTGATCACATATTTGATTTCATTCTATAAAAAAAAGGAAAACAACAT
TGTATCAACCAGTCTCCACATATACAACAAGTATTTACCTGAAATCAATGTAATCATAAA
GCAGAAGTATCCATCACATAGAAGATTAGTCAAGTAACATGCCTTGACGAGTACAATAA
AGATCTATCGTAAAACATGTAGTATTTCTGTTTCAGTTAGCAGGTTAGCATAACAAAAG
AATAGTTTGCCGGCGAATATTGATGCGTAGCATCAAGGTTTTCTTGTATTAATATGTGAG
AACTTTTGGAAGGCCAATGAACGCATTTAGACTAGTTTTCTTTTCCCGACTATTCACTTT
AATGTATTTTGTTATCAAGGCCCTGGGAAAGGCTCGACTGTAGCTGCCAATGTCAATTT
GTTGACCCGTAGTACACATTCCATTGTGCCGATACCCAGCCAATACAACTTGTGCATTG
TTATCTTAATTGTGAAATTTATATTGTGTTGAAGAAAATGAGATAAAAGATAAGTAATTA
ATTTCAACGTATTGAAGCAAATTCAGTTTACCTCTTATCACCAGGACAGCCAAACATCT
TTGATTTTTTTTGGTCACCTATAATGAATTAAGCCCTCCTCCAGCAGGTATCTTTTAAAA
GTCTAGTCAACTTAACACTTTGATTATACATAGTTGTACATGTGTGATATTGATAAGCAAT
CATACAGGAAAAAATTTCCCCAATTATTTTTTATTATATAACAATATTCTAATAAATTTGA
ATAAAAAGCTTTGTAAAATTTTGAATTTTATTAAATTTTCTAATATTAAAGTTAAGTTGAT
GCCATTTGAATTGGTCCCAACAATATTCTTACTAAGAAGATGCTGCTTCTGCATATGCTC
ACACTAAAATCCTTCTCAGAGAAAAAAACTGTTCTGCACAAATCAATATTTTTATAAAA
CTGTTGATCTATATATAACTTAACATTTGATGAATGACATCTGTCTTGAAAGAATTTTATG
ATTGTTTTAATACCAACATTGATAACAATTAAATACCAGTGGTCATTTGAATTGTCCTAAT
TAATTATTAGTTTAAACAGTGCATATAAGTGATAATCAAGAATATTAGTCAAAACCAATC
AGTGAACATTAATTAACTGAACCATCGTGCTGCGAGGTCATTGGCCTCTTGTTTTTAAA
TGTTTTAAATATATATATATAAAAAAATTTAGGTCATCTGACCGAAGGTCAGGATGACCT
ATTGTCATCGTGTTTCGTCCGTCGTCGTGTGCCGTCCGCCATACTGCGTCGTGCGTAAA
ACTATTTATGCAAAAGCCTACTCCTGCTAAACCCTTGAGTGAATTACATCCATATTTGGT
GTGAAACATCCTTGGGGAAGGACAATCATATTTTATATAAATGAGATAGGTCCGACCCC
TAGGGGCAGAGGGACGAGGCCCCAAAAGGGCAAATTTTCTTAATTTAAGCTTTTAAAT

CCTACTCCTCCTTCATCCTTGGATGGATTTCATCCATATTTGGTGTGAAACATCATAGGG
GAAGGACAATCATAATTTGTATAAATGAGCCTGGTCCGACCCCTAGGGGCTGAGGGGT
GGGGCCCAAAAAGGGCAAATTTTCTTAATTTTAGCTTTAAAATCCTACTCCTCCTTTATC
CTTGGATGGATTTCATCCTTATTTGGTGTGAAACATCATTTGGGAAGGACAATCATATTT
TATATAAATGAGTCTGCTCTGACCCCTAGGGGCTGAGGGGTGGGGCCCCAAGAGGGCA
AATTTTCTTAATTTTAGCTGGCCCACTTCCTGTTTTAAGGTTTCAGTCTCCGATCTCAAT
GAAAATTGGTCTATAGGGGTTTTAATTGATGCCGAACAACATGAAAACATTTTCGTAAA
GATTTTGGTATTCCAAGATGGCCACTGGCCCACTTCCTGTTTTCAGGTTTCAGTCTCCG
ATCTCAATGAAAATTGGTCTATAGGGGTTTTAATTGATTCAGAAGCAGGAACTTTAGGT
GAGGACAATCATATTTTATAAAGGACCGAGCTAAGACCCATGGGGATAGAACGGTGGA
GGTCCAAAATGGGAGCTTTGATGAAATTTGGCTTTAAAATCTGACTCCTCCTCATATTAA
TCCTTGAATGGGTTACAACCATATATGGATGAAGGGCTACCAAGTTTGTTCAACAAATG
ACATTTACCTATTTCAGAAACTTACATATTCAACTAAGGAGTTCCTTGTATTGTTTATATT
AATCGTTAACCAATACTTTATACTGTGACTTTGTTGTTTTGTGCCATGAGTCAGATGACC
GTTAAGGCCCATGGGCCTCTTGTTTTACAATATGTCATAAATGTTTATAATACACTGAATT
ATGATCTATTCATGAACAGTGAATGACAACTCATATTTTAAATCATCAAACTAACTAAAT
CCTCCTTAACCTGAGCTACTCTATTAAGGGACCACTCTCTATTAAGAGACTACTCTCTAT
TAAGAGACCACTCTCTATCAATTAAGAGATCACTTTTCAATTTCCCTTGAGTGGTCGCTT
AAGATAGATTTGACTGTAATTTCAATAGTAAAGAAAATAACTGTTGCTAATTTGAGAGA
ACCTCTAATCTGTCACTTTGGTTTCTGTATGAAGGTATATCTCTGAATATGGAGCAGTGG
AACAACCTTAAGGAACAGATTGACGAGATAGACAAGGCCGTGAAAAGTGTGTGAGGG
CTGAAAAACTGCAAAGTGTGTGAGAGCAGAAAAACTGCAAAGCGTGTGAAAGCAGA
AAAACTGAAAAGTGTGTGAGCAGGAAAAAGCTGTGAAAAGTTTATGAGCAGCAGGTG
CAGTCATTTGAAAAACAGAGGAACACATCGTTGCTATCGAATGTTACATCTGAAGAGG
AAACAAAGTTTTTTGAATGGAACTGTTGACTGACAATGTGTCATAAAAAGCCTTTGAA
AATTGTCAGCCATAATCTAATTATAGTAGTATCATTCTTCACTTGTGCATACTGAGAACA
CACTAAGTTTTCACAGATGATAATATTTTTCTTTTCCCTTGCCAAAAGATAATGTTGAAT
AGAATCATGCACATTGCTCTATCTTTCAGAAATTAAAATAACAAGTAGTCATTGAACAG
GGAAATGAATTTAGCATGTAAATGGTACACACACAGATGGAATATCAAAGTGTATAGGT
TCTGTTTTCATTGATTGATCATTTAGATGATGTGAAATACTTCAGTCATGTAACGGTGAA
ACATTAACCATAATTGTGTTCATATCCGTAATTGCCATTGCTTTTTAGCCCATCATTAAAT
GGTGAATGTTATTCAAATCACCTTTCAACCATGGTCTGTGGTCCGTCCGTCCGTCCGTC
TGTCCGTCTGTCCATCAGTCCTTCCATCTGCTAACAATTCTTGTTACTGCTATTTCTCAA
ATTTCATATGAAGGTTCCCCTTGGATTTTGATAGTTATGTTTGTTACAGCTTTTTCTCTCA
GATAGTGCTGAAGGGATCATTCTCAAATTTCATATGTAGGTTTCCCTAGGACCCTAGTTG
TGCATATTGCATTTAGAGATCGATCGGGTCAACATGATGTCTGATAGATAGCCATCTTGG
TTTTTGATAGTTTGAAAAGATGAGAAAAATTCCCTCTTTCTATTGTCAGACATAGATCAT
TCTTTGGTGGGCGCCAAGATCCCTCTGGGATCTCTGGCATATCATGGCTTGTGTTTACTT
TACAAAGTGAGGCTGATTAGAATTGCGGATGAGTTAGGAACAAAAGATCTGGTATTTG
TTAACTACTTTTTAGCTCGCCTATTCGAAGAATAGAGGGAGCTAATTTTGTCACCCCGG
CATCGGCATCGGCGTCCCATTTCACGTTAAAGTTTTTGAGCAAGTTTCTATTTTGTCAAT
TGTTTAAGCTTAAGTCATTGTAAATGTTTATGATTTTATTGTCCTAATGTGTATGGATGCT
GAACGTGATAATACAACCAATTTGGGGCCCTTTAGTTTTTTTTGAGTATGTTAATTTGTC
TTATTTCCATGTTTAAATAGTAAATACTTGAACATCAACTTCTTCTGAATAGGCGAGCTT

TGCTGTTCTCCAACAGCTCTTGTTGTATACATGTACATTATCTCCCTTTGATTTAGAACTA
TATGTCAATATTCTAAGTGAAAAATTCCATAATTTCAATGTATAATTCAGAAATTGTAGTG
GCCATTAAGTATTTCTCATTACAGATGAAATGTCTCTCTTGAACTACAATTTGCTATTAG
AAGTTTTATGGGGGAAAAAAGATTTTGAAATGGAGAGGCATTAAAGACTCTGCTGATT
TTATAAAAATGTTATTTACTGATTTTGTAAACTATTATTATTAAGTGTACATGATCAGTTGT
GCATTCAAGTTATATAAGGAGTAATAAATACTGTATTTAGCGTAATTAGCACCCCTTCCC
TTTCTCCCCTCATCCCATGGATCATACCACGTTCTAAAACCTTCTAATGCATACAACAGA
AAAGTGTCCCAAATTAAATACATGAAATTCATTTTATGGCATGTATGTACCCAGGTAAAC
ATCTTTCGCAAGTCCTTCACATATGTGAATTGTGACATAATAATTTAATATGTCACAAA
AGAAAGAAGGCAAGCCAGTGCTTCACTGGGACAGATTAATGTGTTACGAGTTAATACT
AACGTTTTTGTCGTCCACAGATATATATATTTTGGTTCCCCAATAAGTGCCCCCTGTGTG
TAAAGTTTTGATTGCCCTGCAGGGGATAATTACATCAAATACAGTATGTTACAAATATGT
GATCGTTTTGATTGAAACAGTATTTTTCAACTTATTTCTTCTGTTGACCTGAACTGAAAT
TGTTATATTAATCTCTACCAGAGTTTTTCCCCTTTGATTTATAAATGTAACTTGCTTACTG
TCAGTAAAAGTACTATATGTTCAATAATATCTGTTAGAAGATACGTAAGTACCCATTCTTT
ATTCCAATTGCTGAAAAGTGCTATCATTTGTTCTTTATTGTTATTCAGTAATCATGCATCT
TAATTTTGTTGCATATACATATATATTTTTTTTTTATCAATTGCATTGTCCTTCATTTTTCTT
TTGAAGCTATTTTTCATTAAAAGTTGACAGACTGCTAC

Coding sequence:

ATGATGCCCAAATCTAAGGAATTCATTTCATCATCAGAGTCTGATGTGGACTCTGACGA
GCCAAAGCCCAAAAAGAAGAAAAGAGAACAAGTGCCTGAAAGCAAGAAAGAAAAG
CCTGAAGCTTCTCAAAGTAAAAAGCCAACAAAGGGAGCAAATGGAGAGCAAATGTTT
CAGCTTTCCAAAATGCGGTTTGTCAGTGTCAGTGAATTCCGAGGCAAAGTTTTGGTGG
GAATAAGAGAGTACTATGAGGCTGATGGAGATCTCCGACCAGGCAAAAAAGGTATATC
TCTGAATATGGAGCAGTGGAACAACCTTAAGGAACAGATTGACGAGATAGACAAGGCC
GTGAAAAGTGTGTGA

Amino acid sequence:

MMPKSKEFISSSESDVDSDEPKPKKKKREQVPESKKEKPEASQSKKPTKGANGEQMFQLS
KMRFVSVSEFRGKVLVGIREYYEADGDLRPGKKGISLNMEQWNNLKEQIDEIDKAVKSV

>Arg0193880.1

Gene sequence:

TTTTTTTCATTTTATTTTTATTTTCATTCATTCTTTTAATATAGTATAGTACGGTATGTAATA
CATGGACTTGGTACGAATGCCAATCAACAAAACACACACACAGCTTTGAAACGAATTT
TCAACCCACGGTTATATATATTAATAATTCGTTGTACACTTCCTTAGCTCGGGCTAACAG
CTACGTACACGCAGGCTACAGTTTCATGGCCGGGATGTGCAGGATCACATTTTTTTTTT
TTGAAATCAAATGAATAAAAACTTGAATTAACAAAATCAACGTCGATCTCTGCTGTATG
GCTTTTGCTGCATATACACAGTGCTCTATGAAAGGGTGGCCTTTGATTTCCACTTTCAA
GACCAGATGTCGACATCTTTTAGTTTAGATGTCGACATCAATAACTGACAAGTCGGTGT
CACACCCGAGCATAAACACATTGATCGCCGAGTTACCGACTGTCTACATAAATATCTTG
GATTCATTATGTGGGCATGTTCATGTGGTAAGTCGACATATTCTTCTGTTTATGTCGACAT

ATTCCGATATGATGTCGACTTAATGCCTTAACGATGTCGACATTATTAAGTCGTAAGTCG
GCAAATCGATGGCAGAAATATGCCACCATAAAATTGCAATATTTTCTAATTTTCGGATTA
ACGGGCCTTCGAACTATTAGGCCTTAGCGGTTCAAGTGACCTTAAAATTAACAGATGAC
GATTACTGCGTACTAATCAGACGGCGTGACCAGACCATTATTACACGATTATCATATAAT
AAACAAATTATTGATTGAAATTCATAGTTCAGAGAAAAATACTTTGACAACTCTCGGTA
AAGAGAAATTGCTATCAAATTTCTTCATCTAGGTACAAACACATGCACACAATTATTTTC
ATTAGCAATCCAATATGGCGAGTGTTATCCATTGTTTGCGCATGTGTAACAGGAAGGCG
ACAAGTCGACAACTCGACAACACGACAAGTCGACAACACGACAAGGCGACAACTCG
ACAACACGACAAGTCGACATTTACCGCGACAACACGAGATTCTGTACGCGCTAATTA
GCGTGTTTGAAATGTCGACTTGTCGTGTTGTCGACTTGTCGTCTTGTCGTGTTGTCGAC
TTGTCGCGGTTCGAAAACGACTAGTCGACATTTTAACTGTTAAATCTCGGGTTGTCGCA
GTTTGAGACCGCGACAACTCGACAAGGCGACAACACGACAAGGCGACAACACAACA
ACACGAGATGGCCGTAATCAGCCACCATAGAAAAGTGTTTATTTTTGAACTGTATACTT
TGGTCGTATATTCGCCTAGTCAAAACGTGCATGCATGCCAAATGTCAGGGGTTACTATTA
CAGACGTTATACCCACCATGGACTATCTCATAGTAAAACTGGATGCAATAGAACAGGTA
TTTTAGTCCTTTGGTATACATCAAAAGATTCGTATTACAAAACACTGTGGTTGACTGTGT
GGCTTTGAAGTTGGGCGTCGCTCTCTCTGTAGTCTTCAAAGGAATTTTTATTGGCCTGT
GATTGGTAATATTTTTTCTTCTGAACTGCCTTCAGTTTTACTATAGTCCAGGGGTGGACA
TTACGTCAGTGATAATAACCCCTGGAGTATCGAGGATGCCCCGCATCCGACATCACCGT
TTTATGCTGGTAAATGAGATATCAGATATCGACACAATACGTATCAAAGCTGTCAACAA
GGTCTTCGTTTACTTCTGGTCGAAGTTGCACGAGGTTTGCCCATTCTTCAGGTGAGAGA
CAGACAGGTAAGTAATACGGTGCTGACGCCTTCCCGTCCACGTAAGTAAAACTTTCATT
GTACCCTATCCCAACGTCAACTTGTTTGGTGTTATTTTCAAATGTGGTCTTCACGCATAC
AGGTCTTCTTTCGGACAAATACAGCACTACATCCACTGGAAGAGTATGTAAAAACCCAT
ATATAACGGAATTTTGAATACAATCGTCAATTAATTACCTTTGATTCTTCATTAAGGTAGT
ATAGGGAGGGGTTTCAGAATGAACAATCCAGACTGGCAGTGATAGTTATCTAATAAATG
AATGTGCTACTAAGTTTGACTCCTAAAGTTTGATGACTTTTTCAGAAATCTCTTTGATTC
AGATTGATTTCTCCATTTGTAATATTGCATTTTTTTTTTCGATTTTAACAATCATCCCAAA
ATTCGCGATAGATTTTTGAAAATATGGTGATCAAACCTAACTGCATGTACAAATTACCCA
AAATATAGCCATACATACCGGAATCACCAACACATGACGAATGCAACCCAGGTATGTCT
CTCCGCTCTTTCTGTGCTGATGCGTCATCATTATTTTCAGCAGTTCTAATGGCTTTGTCA
ATGACGGTTCGGCCCTTGTAAAGATCCTCCCATTCCCTAATGGACAAACGGACACCATC
TGAGGTAGGGAACCATGAATTATCCGAGTCTATATAATGATACTCGTTAAGAATTATAAA
AACGCCTTTCTTGTACTGTTTGATTTCCAAATATTTCATAGAGTCTTCATGAATACGGAA
CATGTGTTTTCCAGTTGGTCCTCCCTGTCGCTTCGCCTTCTGTTTTTGTGTCTTTGTCTC
CAGTCTACAAACCTTGGAAGAACCTGCAGCTTCACTTTTATTCGCTGCACTACGTTTTA
TCCCGCGCCGCGACGTCCCCGCGTCTTTTGACGAGCTTTCCGTTTGATACTTCTTATCT
GTCGAAACCATTTTCTGACTATCGTTCGATTTGTGTGACGTTTCGGATGAATTTTCCTTC
TGAAACGCCTTCAGTGCTGATGTTATTTTTCTCTTGTCATTTGATTTTAAATCCAAAAAT
CCTTCGAATGAGGGGAAATTGGCATCTTCATCTTTCAGAACATCCGCGAACTTTCGCTC
CTGCCAGAAACACTTAAAGTGATACCATTGTTTTTCCTCTTGATTCTCTTTTCCGTAATA
ACCAACCTTTAAAGAACCTTTGGTACCTATCTTGGCCCCACAGATAATGCATACATCGT
GTGAGCCTCCTGAATTCGCGTATTCAACACAGAAAATGTCTGCCATGGTAGAAGAACC
TCTAGTGGCCCTATAGGATTGTTTATATAATCCTCGTACAATGCAGAATTAGAACAAAAG

GAAGGCGACTTGTGAGAGCAACACTGATACTGGATACGATACACAATATTAACTCATCA
CTTCATTTCCGATATTTGTAGGGTCTTATCAAAGCAGGTTTGGACGTGTTCTTTACAAGT
TGTGATACTAACTTAAATGCTGATTGAGTGTTTATTGTTGATATCTATACCGTATACTATAT
CTGGATACCGAGTATTGATATCAGTGATAAAACGATAGTTCGTTGACTGAACTGAATAC
CTATACATGTCTAATACGAAATTATGATGCAGCTCTTTTGTAGATGCTTCTGATGTGTTCC
GCTTTTGTCTATTTTTGCAGTGCTGCTCTTTGTATACCGCTTAACCGGCAGTTTATATAAC
GGAATATGGCTTATTGGTAACTATGTACTACAAGCCTG

Coding sequence:

ATGGCAGACATTTTCTGTGTTGAATACGCGAATTCAGGAGGCTCACACGATGTATGCAT
TATCTGTGGGGCCAAGATAGGTACCAAAGGTTCTTTAAAGGTTGGTTATTACGGAAAAG
AGAATCAAGAGGAAAAACAATGGTATCACTTTAAGTGTTTCTGGCAGGAGCGAAAGTT
CGCGGATGTTCTGAAAGATGAAGATGCCAATTTCCCCTCATTCGAAGGATTTTTGGATT
TAAAATCAAATGACAAGAGAAAAATAACATCAGCACTGAAGGCGTTTCAGAAGGAAA
ATTCATCCGAAACGTCACACAAATCGAACGATAGTCAGAAAATGGTTTCGACAGATAA
GAAGTATCAAACGGAAAGCTCGTCAAAAGACGCGGGGACGTCGCGGCGCGGGATAAA
ACGTAGTGCAGCGAATAAAAGTGAAGCTGCAGGTTCTTCCAAGGTTTGTAGACTGGAG
ACAAAGACACAAAAACAGAAGGCGAAGCGACAGGGAGGACCAACTGGAAAACACAT
GTTCCGTATTCATGAAGACTCTATGAAATATTTGGAAATCAAACAGTACAAGAAAGGCG
TTTTTATAATTCTTAACGAGTATCATTATATAGACTCGGATAATTCATGGTTCCCTACCTC
AGATGGTGTCCGTTTGTCCATTAGGGAATGGGAGGATCTTTACAAGGGCCGAACCGTC
ATTGACAAAGCCATTAGAACTGCTGAAAATAATGATGACGCATCAGCACAGAAAGAGC
GGAGAGACATACCTGGGTTGCATTCGTCATGTGTTGGTGATTCCGTGGATGTAGTGCTG
TATTTGTCCGAAAGAAGACCTGTATGCGTGAAGACCACATTTGAAAATAACACCAAAC
AAGTTGACGTTGGGATAGGGTACAATGAAAGTTTTACTTACGTGGACGGGAAGGCGTC
AGCACCGTATTACTTACCTGTCTGTCTCTCACCTGAAGAATGGGCAAACCTCGTGCAAC
TTCGACCAGAAGTAAACGAAGACCTTGTTGACAGCTTTGATACGTATTGTGTCGATATC
TGA

Amino acid sequence:

MADIFCVEYANSGGSHDVCIICGAKIGTKGSLKVGYYGKENQEEKQWYHFKCFWQERKF
ADVLKDEDANFPSFEGFLDLKSNDKRKITSALKAFQKENSSETSHKSNDSQKMVSTDKKY
QTESSSKDAGTSRRGIKRSAANKSEAAGSSKVCRLETKTQKQKAKRQGGPTGKHMFRIH
EDSMKYLEIKQYKKGVFIILNEYHYIDSDNSWFPTSDGVRLSIREWEDLYKGRTVIDKAIRT
AENNDDASAQKERRDIPGLHSSCVGDSVDVVLYLSERRPVCVKTTFENNTKQVDVGIGY
NESFTYVDGKASAPYYLPVCLSPEEWANLVQLRPEVNEDLVDSFDTYCVDI

>Arg0230340.1

Gene sequence:

AAAAAATCGTATCCTAAAAATGATAGGGGTACATGCTAAATCGCTTCAAAAACTCGATA
TTATTTCATTTATTTATCGACACCAATAAAAATAAGAACAGTGAAAGCGTTAAATTTCAA
TTTGTTCCACCATTGTATATGAAAATGAGAATAAAGTTCCCTTTTTGTATTCATACATGTA
ATCTGGATCGCATTTCCGTACAAAACTGAGAAAAAATGTGTGAAAATATGAAAATATCG

GATGCATTTCATAGAGTTAAGATGTTGAATGAAGACTTGGATAAAACAAAAGTTCAAAT
TTATCTAAAATACATCCTTTGTGCCATAATCGAAGATGTTTAGTAAGCATGCTATGACTAT
AGTGTACTGGTTAAAAATTTGAATCATAAATAAAAACATTGGATAACTAAGTGACAGCT
ATATAATTTCGCGCAGTTAAATAAACACCAGTAAGAGCCGATACATAATTACCATCAAA
GAAAATATTTACATTTGTATACATATAAGTTTGTAACCCTGGTGTGTGCATAGTAGCTGTT
ATATAGCTTCGTCATATCAAACTTTATAACGGGGAAAGCCGGAAAAATTACTATCCGGAT
CCCGTTACGATATTGAAGTTATATTATGTTATAATCATTCTGATGATCATTCCAGCTATTAA
CCGGCCATTTTTAAATACCCCACTCTTCTGCTGGTGTATTGAACACAGACTAAGTACAC
GCATGTGTGTGTATGTGTGCTAACTATGTTACCATTAAGAAAACAATATGGCGGATAGTT
AATGTACATTTATAGGCCGTTGTTGACTATTACGAACATTCCCGATTATCTAGTGATTTGA
GTGGAGCATCATGTTAAACAGAACAAATGATCGGTAAGTGATATATAACAACGACATGT
CTACATTACAAACTTTAACAGAAAGGTTACCGCTAACCCGCCATTAGGTTGTCGATTTAT
ATACCCGATACATTGTATATACCCGATCTCCGTCTATATTAAGCAATGCTTCTCGACCTCC
TATAGTCGAGCTTATTGACTATATTGACCTTCTATTGCACGTTGCCGCGCTGAAATGTGG
TCACCAGGTTTATATTGTGAACCAGCATAGTATCCAGGTGACTTTAAGTCCGGGTCTTG
TGAAAAGTAGTTTTTTTAGCATTGATGCCACTATATTGTAACTTTATCGGTAACAATGTAT
GAAAGAATTTTTTGTCTCTAAACTGTGTAAATCCGCGTAGTACATTCTTACAATAATTTG
TATACGAAATTTATTTCATAATCCGATGCAAACTTATCAGAGCCTTGTTTGTATAGCAATG
TCAATTTTGATCTATCAGAGTTACTTCCCTTCTTGTCACTGCGTCAAATTCTTTTCCATGA
TGCTCCACCGCCGACAGAGCATGGGTCCATATAGAGGATTTTATATGAGTGTTTATCACA
AAACTAGACCGTCAAGCCATGTCATACGGCCATTTATCCTGCAACTGCAACACATACTG
ACGATTTAGTCTACCTCGTCATCTCGGCTTTCCTAAGTCTCGCACCAAAATAAACCTTGT
ATTGTAAGATACTTATCGTGTATTCTCTATGTGATAAATATCGTAAGACACGTGTGTAATT
AATATTACTTTTATGACGTCACAGGTAGTTATATGATGTCGCATGATTATCTCAGAAGAC
GGAAGCTTCTCATCCGAGCCGGATTTCAGCTGTTTTATATAGAGACGAAATCTTAAATTT
TAGCTATATTTCTATTTATAACTGCTATGTAATATATAGAATCTTACACTCGCTATGTGATAT
GATATTTATCAAACCTTTGAAATGATCTGATATGCTACTCGACTAAAGGCTCGTGGTATA
TTCAATTATTTAACTCGTTTGATGAATTTCATATCACATAGCCACTCATGTAAGATCCTGC
ATTTTTGTAACACAATATGAAATGTTCTTAAACGCGTGGAAATAGTATATCATGTTAACA
TTTAAATCTCAAGATATCAAATAATGTAAAGAGTTCAATTAATATCATACTACGTAACTAC
GTGCATAAGATCATATTTAGCACATAATTAAATCGAAATATCGGCAAACCATTCATTTTC
GGTTCTATATAAAACTGACGAAATTCGGCCCGTATTTGTTATTTCCGTAGTCTACCGAGA
AAATCGCATGATAATATATTTTGATCATGACGTCATTATCATTTATGACTTCACTATATGTC
TTGTGGATATGTAAGACATGGTTATATGTCATGGTTGAACGGTACAGTTATGTGTTAAAT
ATTGTTTTCGATAGCGCGAAACACAAATCACTATATGCAAACTACATCTCTCATCCGACT
TATCAGAAGTGTAGAAACCACTTAGTAATAGGCTAATTTTCATTTTTTAACAAAACACTA
ATTTTGTATTCAAGATTTAAAATTCTTTTGAAAATAACCAGAAAAGAATGTTTCTACTTT
TATAAGTTAAATATTTAATCATCGTTCCTTTTCAGCCAAACCTGTTTCTAAGAAATATATA
CACTTATGTTATGAAAGGTTTCGAGAAATACTAATAATATCAATACCGTGGAACTCGGTT
AATACGAACTCGAAGGGACCGAGATAAAACTTCGAGTTATCCGAGTGTTCGAATTAAG
CGAGTTCTCATGTTTACTGACTATCTCTTTACTTTGTATATATAGACTAGTTCCATGTCGT
AAAACGATATGAACAAGAGAGATCGCTGATGGAAGTTGCAAATTGATAACAAGAAAA
CCTAGATATATATTTTGAAATGCCGTTCTACGGCAGATTTATGAAAAAAGTTATCGGCAT
TTTTGGCGATCTCATTGTCCAAAACATCTCATTTCGAGTTATAAGAACATTTTATGTATGA

TTTTTGCCATTCGGACCCCCAAATTTACTTCGTATTATCCGAGTTCTTCGAGTTTTCCTAA
TTCGAATTATCCGAGTTCTCTAAACAAAGATAAAGAGGGAATTCTGCCTGGACCGTCA
AAGTACTTCGTATTAAGCAGTGTTGTCGAATTATCCGAGTTCCATTGTATTTTATTTTTGT
TTCAGATGAGGAAGCGAAAACATGGATTTAGATGAAGTAAGACTGAGATCTTAAAATG
GGATGAAAAACATGAAAATAAATGATGCTTCATGGTGAAGGAGAAAACATTTTTGTAC
ACAACTATTGAATATTAACCATTAATTTCCGATAGGATATGGCTGCAGAGAACACGGAG
TCCCCGAGCCATACCTGCACACTACATTTCCTGATGTCAAGTTTCAGAGAAATGCAACA
ATAGAGGGAAGGACAATAACTTTTTGCTGTCATTATATCAATTCCAAATAGAACTACTCC
ACCTACAAAAGGGGATAATTGAAGCAGGATGAAATAATTCAAATTACCAGTGTAGATTT
GTTAAAACTATGACATATATTTTCAATCGATCTCAATGATGCCAGTGAAATTCTGTGAAA
CTTGTCATCACGCAATGTGAATATAATATTATTATATATTCTGTGTGTCAATTGCATCACCT
TTATCGGAGATAAATATGAGTTTATCACAAGCATTTGTTTGTAATTACGTCCTAGACAAA
TGTATCTGCATAAACTGCCAAAGAAACATCGATAAAGGTTGTTTTCGTTTAGGCCTGGT
CAGAGGTCAAGGTTCAGAGGTCACTCGACATGACTGGTATCACCCGATATGCTTCTGG
GAAAAGTGTCCATACAAACAGCATGTTCGTGGGACGAACATTGACCGCCTGACATTCG
TAGAGCTGTTTCACGGGTTTGAAAGTATAGCAGCTTCAGATAGGATGAAATTGGAACA
AGAAGCTTACAAGCCTCCTGCTAAACTAAAACGAAATCATCGCCATTCCGGATTGGTTG
CCAATGGTAACGATGACGTCAGTCTTGATGGTGGTGACTATGAACTCGATGAGAATACG
TATGATTTGTCGAACTTGATTTGTATACATGTGTTCAGGTATAAACTTGATGTGTATGTAA
GCATCCGGGAGTATTTCAAAGCCGTGAATGCAACGATTGCCAAGGCTACTCAAGTCGG
CATCGCTTTGAAGTCAAGCCAATGGCATGCAGTTTGTCGGAAACGATTCGGAATAGAC
TGTGCTCTTAAAGAAATAGGTGACAGCAAGGCAAAAATAAAATCTAAAGGAAACGAC
AAAATCGAAAATGATAAAGATGAGGATGAAGAAGATGGTAAGAACGACTATTATTATCA
CATTACCGTAAATCAATTTAGTATAATAGCTTATGGCTTCTATTGTTCTACTTTACCTGTA
CTTGAAGCATTTTCTCGACTATTTCCTGTCTCTTAAAATATGTGAAACCTTATTGCAATC
GGATAAGTAGGCTAACAGTGTCTCTTGAAAATTGTCTCTCTTATGCCTTCTTTATCCCCT
TTCGGGAGACATCCTATATATATATTGTGTATACATTTAAAATTGCATATACGAAGCATTT
CGATATGTTTATCATACTCATAACAGTTCTAGAGATTTGCAATATAAATACAGTGCGTATT
GTTATTTAGTTAGTTTTTGGTATAACCGAATCGCAGGATATTTTCATGATAGAACACTATA
TATATTTCAATATGAAAATTTGCATGCATATGATTACTTCGATTTAGTTTTCTGAGAGTTA
GCATGAACACAATATCTAGGGTGATTTACCATAATCCTTTTTACAGAATGCCAAATAATT
CCACTGACTTGATTTGTAGGTCAAGTCGTGTTTTCGTTGGCTCGGAAACGAAGAATAA
GTATATTCCGAAAGCAAAAAGAGGCTGTTGTTGATATTCGGGACTTCTCTGAAGAAAG
GACTTTCAAACCAGGGAGTCGAGGAATAACCCTTTCTAGAATTCAGTGGAATAAACTT
AAAGCATTGATTGATTGCGTTAATTTTTCAGTGCTTTCACTGTCAAAAACTGAGTGAAT
TTTGAATAAAAAAGAACTACAAATTTACAAATTCAGATTCAGATTATGTTTTGGCAGTG
TTAATCAACAGAAAAAACAACATATATTTGTACATGTAAAAAAGCTGTCTTTACTATTTT
TTGTATAAGACGCTTACTTATTTCTAAACAAAACACAAAAACCAGAGACTAATCTCATC
AGAAAATTGTGTGAAAGAAGATGTTTCTGTTAAGTTATTTTCCATTACCAGATGTTTTAC
GTATCCTCTATTTTCATTGCACGTTTATGCTTATCTTATGTACATTATTTCGTATTGTTGAA
CAAGTTGTAGTTGAAGGCGATTTTAGTCCCAATTGCGAACATAAGAAGGCGGTATGTG
GTAAAGAAGCAGTAATATGATGCAATCTTAATGGGCTACATAACGTAAGCTTCATGTCC
CCGCTGTTACGATTTTACAATCGCTACCATGTATCAATCGACCAGTTTCAAAAACAGGG
AAGTAAGGAAAAACAAAGCGATTTTTGAAAGAAAGCAACTTGTTCCTTCTTACGTGTG

TGTACTGCACAATGTTATAATGACATCACAATATTTTATGTCCTATATTGCGGCACTTCA
ATCCTATATTGCGGCACTTCAATTCTATATTGTGGCACTTCGAAAGTGCCAATTTTTAGG
CATATAAAACATGTATGTGGCCAATCAGAAGCCGTTATTATAGATGTATTAAATTGTGTA
AACCACTTGATATTCGCAATTAAACTGTTTGCGCAATTTGGACAAACGGTGTGAACGC
GAATTTGAATATCATTATATATATAACTGTATATAATGTATTGTGTATTTGCATATTACCGAG
TTGCCTGCTCTTGCGGGTAGGTATTGATTATGACGTCATGTGCTTACGAGTGTAACGTCA
TATACATTTCGAAGAAATTGACAAACAATCACAAAATAATGACGTAACAATTTGTAATAT
GCAAAGAAGGAATACACTGTATATGGATATTTAGGATTTTCAGCAAGATATTTTGATCAG
GAATTCATGCATTTATCGATATGCTTCAACAAACAAAAAAGGGATATGCTGTAAACGCC
AAAATCAAATTAAAAAGAAGTTTACGGTATAGTACAATGTACACAATGAATATGACTAA
AAGTCCTTTAATATATTCTTTTTTGTAATTCCATAACCCATGTCACATTGATAGTTTTGGT
AAATACTATTTAACTTAATTTCTAAAACCATGTACTATAATTTCAGACATTTATTGAAATG
GTTAAAATCGATAGTATTTAATGCATAAAATGTATCATATTTAAGTGGCACTCAGCGTCTT
AGATCACCTTAAATCCAATAATAAGTCATTCAGAAGCGAAGTTGAAAAGTTTAATTTCC
GAGGGCAAAATCATGATACAGTTGTTTTAAAAACACATTGTCACTCCACAAATAGATAT
TAGCGTGCAATGTTCAACGAGGGTATATTTTTACAATGTTTTATATAGTTTTCTGGTAAC
AACTAGCTAAATGTATAATTGACCTTCGAAATGGCCACAGTGTAAAGAATGAGTTTAGG
ATAGAATTTTGACCCGGTCACTGATTGGCTTGCTAATTATTAACTTCAAAAGTAAAAATG
CTTTCTGACAATTACAAGAATTTAGAATTCCAGATTTAAATTTAGATTCAAAATAACAAT
TTTGACAATGAATAAGTAGAACATTGGAAACTCATGATTTTTCAATGCAAAATGCACCT
TATTTTAAAATCGCTACCCTTGTTGAGCTAAATAACCCAATCTTTTTTCTGATTAGTGTTA
CATGGGACTCTAGACTTTAAATATAGAACTCTATAGCCACTTAATATTTAGCTGTTTTAAT
TAAACATTACAAAACTTTAACAATGTGATCTGTGTTAAAGGATTTGATTGGTTCGTTTCT
TTGTGTGTCGTTGTCATATTGGTTTATCGATTTAAGTACAGCACAGGGAAGTGTCCGAC
ATTTCTCTCGGGTATCACCACGATTATTCCATTGAGCTTGAAATTCCTGTTGTGTTCCCA
AGGTACACATAGACGTTGTCCTTGGACGACTTGTTTTCGCAAGATTGATATTAAGCACA
TATACTGGATTGGAAAACTCTTCATTATGGTTTTTTATGATGACATCAAAGGAAACTTGA
TATGTACTGGATTCTGGTAAGTTGATTAAAACAATATATCACACAGTTATTTACATTGTA
TAGTTCTACCGAAATAAAATCTTGATGTTACTAAAATGCATATCAATACGGAATATCAAA
GGAATTGTCCTTTACAATGAATCTGATTCATGACCATTATTTAATCCTTTTATTCGGGAGA
AAATGCTGTTAGGTTGTGCATCTTGATTATTTTGAGGCATGAACTTTCTAATACCAGAAA
GATCCATCAGTTTGGAAACGAAGCCCATTCCTATAGGGTTGGTGTCATCATCCTCCTCAT
CTTCATCATCTTCATCATCTTCTTCGATATCCTCCTCCTCCTCAACAACCTTCGAATTGTT
TCGTTTCATTTCCTGGATGACAGAATGTTGGCCAATTGGCTGTGGGGGATGAAAATTTG
CTACATTTCCATGAACGGGAGGATGCGGAATACGGTACCTAGATGGCACTGCCCTTCGA
TTACGTGAACGATAGTAGGGCCGAGATGCCGGTGGCCCAGGATAACGGGGACGTACCC
GTTGATTATTGTAAGCCTGAGAGTATGGACCTGGTGTGGGTTTCGGGTTAGGACCTTTT
GTTGGGTACGGTCTTGATGTCGGGTACGGTCTTGATGTTGGGTACGGTTTGGGAGGAC
GTGGACCGGGACGTGGAGGCATAGGAGGCGCCGGAGGGGCAGGTGGCGCCGGAGGG
GCAGGTGGCGCAGGCGGCGCTGGGGGCATCGGGGGCGCAGGAATCGATGGTATTGAG
TCAGGATTAACAGGTCCTTTATAAGTTGTAGATGTTGAAGCAAACACTTGACTGGCAA
AAAACACAGTAGTAGTTGTCAGACCAGTGTTGTAGTCAGGGTCAGGATTTGGTTGATG
CGTTGTTATCGATGTCGGGTACTCTGTTGTTGATCCCGAAGAAGGGTCCGTCGTTATGA
CAGAACTTGAAGTGGTTTCACCGGTCGTCGAAGTTGGAATAATTGGAGTGATACTACTT

CCTTTTGGACCTGGTTGGGTTGTAGTTTTCGGAGGTGTACTTGTTGTGGTCGAAGAAAT
TGTTGAAGTTGTAGGAGATGTGGTTGTAGTTATTGGAGATGTGGTTGTAGTTATTGGGG
ATGTGGTTGTAGTTGTAGCAGATGTAGTTGTAGTTGCAGGTGATGTTGTAGTTGTAGGA
GATGTTGTAGTTGTAGGAGATGTTGTAGTTGTAGGAGATGTTGTAGTTGTAGGAGATGT
GGTTGTAGTTGTAGGAGATGTTGTAGTTGCTGGAGATGTTGTAGTTGTAGGAGATGTGG
TTGTAGTTGTTGGAGATGTTGTAGTTGTTGGAGATGTAGTTGTAGGAGATGTTGTAGTT
GTTGGAGATGTTGTAGTTGTAGGAGATGTGGTTGTAGTTGTAGGAGATGTTGTAGTTGT
AGGAGATGTTGTAGTTGTAGGAGATGTGGTTGTAGTTGTAGGAGATGTTGTAGTTGTAG
GAGATGTTGTAGCTGTAGGAGATGTTGTAGTTGTAGGAGATGTGGTTGTAGTTGTAGGA
GATGTTGTAGTTGTAGGAGATGTTGTAGTTGTATCAGATGTGGTAGTTGTAGGAGATGT
GGTTGTAGTTGTAGGAGATGTTGTAGTTGTAGGAGATGTTGTAGTTGTAGGAGATGTTG
TAGTTGTAGGAGATGTGGTTGTAGTTGTAGGAGATGTTGTAGTTGTAGGAGATGTTGTA
GTTGTATCAGATGTGGTAGTTGTAGGAGATGTGGTTGTAGTTGTAGGAGATGTTGTAGT
TGTAGGAGATGTTGTAGTTGTAGGAGATGTTGTAGTTGTAGGAGATGTTGTGGTTGTAG
GAGATGTGGTTGTAGTTGTAGGAGATGTTGTTGTAGTTGAAGGTGATGTCGTCGTTGTT
GCAGGTGATGTTGTAGTGGTAGGAGATGTGGTTGTAGTTGTAGGAGATGTGGTTGTAGT
GGTAGGAGATGTGGTTGTTGTTGTAGGAGATGTGGTTGTAGTTGTAGGAAATGTGGTTG
TAGTGGTAGGAGATGTGGTTGTAGTTATAGGAGATGTGGTTGTTGTTGTAGGAGATGTG
GTTGTAGTGGTAGGAGATGTGGTTGTAGTTGTCGGAAATGTGGTTGTAGTTGTTGGAGA
TGTGGTTGTAGTGGTAGGAGATGTGGTTGTAGTTGTCGGAGATGTGGTTGTAGTTGTAG
GAGATGTGGTTGTAGTGGTAGGAGATGTGGTTGTAGTTGTAGGAGACGGGGTTGTAGT
TGTTGGAGATGTGGTTGTAGTTGTTGGAGATGTGGTTGTAGTTGAAGGAGATGTAGTTG
TAGTTGTTGGAGATGTGGTTGTAGTTGTAGGTGATGTGGTTGTTGTTGTAGGAGATGTG
GTTGTAGTTGTAGGAAATGTAGTTGTAGTTGGAGTTGTTGTAGTTGGGGTGGTAGTTGT
CGGATTGGTAGTTGTTGGAGTAGTTGTAGTTGGAGTTGTAGTTGTTGGAGTAGTTGTAG
TTGGAGTGGTTGTTGTTGGAGTAGTTGTTGTTGGAGTGGTTGTAGTTGGAGTAGTTGTT
GTCGGAGTGGTAGCTGTTGGAGTAGTTGTGGTTGGAGTTGTAGTTGTGGGAGTAGTTG
TAGTTGGAGTTGTAGTTGTTGGAGTAGTTGTAGTGGTTGTTGTTGGAGTGGTAGTTGTT
GGAGTAGTTGTAGTTGGAATGGTTGTAGTTGGAGTGGTTGTAGTTGGAGTTGTAGTTGT
TGGAGTAGTTGTAGTTGGAGTTGTAGTTGTAGTTGGAATGGTTGTAGTTGGAGTTGTAG
TTGTTGGAGTTGTTGTAGTTGGAGTTGTAGTTGTTGGAGTTGTAGTTGTTGGAGTTGTA
GTTGTTGGAGTAGTTGTAGTTGGAGTGGTGGTAGTTGGAGTTGTTGTAGTTGGAGTTGT
AGTTGTTGGAGTGGTTGTAGTTGGAGTGGTGGTAGTTGGAGTTGTTGTAGTTGGAGTT
GTAGTTGTTGGAGTTGTAGTTGTTGGAGGAGTTGTTGTTGTCGTTTCCGGAGGAGATAA
TGTGGTTGGAATATTACATACCCCTTCC

Coding sequence:

ATGAGTTTATCACAAGCATTTGTTTGTAATTACGTCCTAGACAAATGTATCTGCATAAAC
TGCCAAAGAAACATCGATAAAGGTTGTTTTCGTTTAGGCCTGGTCAGAGGTCAAGGTT
CAGAGGTCACTCGACATGACTGGTATCACCCGATATGCTTCTGGGAAAAGTGTCCATAC
AAACAGCATGTTCGTGGGACGAACATTGACCGCCTGACATTCGTAGAGCTGTTTCACG
GGTTTGAAAGTATAGCAGCTTCAGATAGGATGAAATTGGAACAAGAAGCTTACAAGCC
TCCTGCTAAACTAAAACGAAATCATCGCCATTCCGGATTGGTTGCCAATGGTAACGATG
ACGTCAGTCTTGATGGTGGTGACTATGAACTCGATGAGAATACGTATGATTTGTCGAAC

TTGATTTGTATACATGTGTTCAGGTATAAACTTGATGTGTATGTAAGCATCCGGGAGTAT
TTCAAAGCCGTGAATGCAACGATTGCCAAGGCTACTCAAGTCGGCATCGCTTTGAAGT
CAAGCCAATGGCATGCAGTTTGTCGGAAACGATTCGGAATAGACTGTGCTCTTAAAGA
AATAGGTGACAGCAAGGCAAAAATAAAATCTAAAGGAAACGACAAAATCGAAAATGA
TAAAGATGAGGATGAAGAAGATGGTCAAGTCGTGTTTTCGTTGGCTCGGAAACGAAG
AATAAGTATATTCCGAAAGCAAAAAGAGGCTGTTGTTGATATTCGGGACTTCTCTGAAG
AAAGGACTTTCAAACCAGGGAGTCGAGGAATAACCCTTTCTAGAATTCAGTGGAATAA
ACTTAAAGCATTGATTGATTGCGTTAATTTTTCAGTGCTTTCACTGTCAAAAACTGAGT
GA

Amino acid sequence:

MSLSQAFVCNYVLDKCICINCQRNIDKGCFRLGLVRGQGSEVTRHDWYHPICFWEKCPY
KQHVRGTNIDRLTFVELFHGFESIAASDRMKLEQEAYKPPAKLKRNHRHSGLVANGNDDV
SLDGGDYELDENTYDLSNLICIHVFRYKLDVYVSIREYFKAVNATIAKATQVGIALKSSQW
HAVCRKRFGIDCALKEIGDSKAKIKSKGNDKIENDKDEDEEDGQVVFSLARKRRISIFRKQ
KEAVVDIRDFSEERTFKPGSRGITLSRIQWNKLKALIDCVNFSVLSLSKTE

>Arg0233380.1

Gene sequence:

TCAAGCAATGTTTTGATTGGGGTTGCAATCAAAGCATTCAATCATCCCCATCTGGTTCT
GATGAGCGTCTTTGCACAGTATCACATCATCAAGTTCGTCACCGATGAAATCTGGTAAC
AACAGCATACAGTCTTTCAGCTTGTCCCATTGTTCGAAGTTCAGGGCAATCCCTTGTCT
CGTGGGTACCAATCCTCCATCTTCATCCTTGGGCATATACCACCTCCTGATATTGACACA
GCCATATCCACTATTTATGCTCGCGTGGAGGTTTCCGCCCAAATGTAACTTCATGTCAAT
GTGTTCCCCTTTCTTGACTTTTTCTATTGTGTCGTCTATGGTACCGGAACACCTGTCCTC
GAGTCTCTTCCACCTCGTGAGATTGAGAGCAATGCCCTTCTTTGTCGGAAACGTTGTCC
CGTCCTCTCTCTTCCCGTATGTTCTGATGTGGATCAAAGTCTCTCCGCGAAACATATTAG
CCACCACATAAACTTCTTTGCCTATGTGTAACTTGCAAAAATCCCCCATCAACATTCTCT
TTATCGTGTTTCGCGGTATTGGAGTTGCAACGTCCTCGCTCCTTTGTCGTTTGGTTCCTC
TGCTTTCATGAGTTTCGTCTATCATCAGTTTTCCGGTGGCCAT

Coding sequence:

ATGGCCACCGGAAAACTGATGATAGACGAAACTCATGAAAGCAGAGGAACCAAACGA
CAAAGGAGCGAGGACGTTGCAACTCCAATACCGCGAAACACGATAAAGAGAATGTTG
ATGGGGGATTTTTGCAAGTTACACATAGGCAAAGAAGTTTATGTGGTGGCTAATATGTT
TCGCGGAGAGACTTTGATCCACATCAGAACATACGGGAAGAGAGAGGACGGGACAAC
GTTTCCGACAAAGAAGGGCATTGCTCTCAATCTCACGAGGTGGAAGAGACTCGAGGA
CAGGTGTTCCGGTACCATAGACGACACAATAGAAAAAGTCAAGAAAGGGGAACACAT
TGACATGAAGTTACATTTGGGCGGAAACCTCCACGCGAGCATAAATAGTGGATATGGCT
GTGTCAATATCAGGAGGTGGTATATGCCCAAGGATGAAGATGGAGGATTGGTACCCAC
GAGACAAGGGATTGCCCTGAACTTCGAACAATGGGACAAGCTGAAAGACTGTATGCT
GTTGTTACCAGATTTCATCGGTGACGAACTTGATGATGTGATACTGTGCAAAGACGCTC
ATCAGAACCAGATGGGGATGATTGAATGCTTTGATTGCAACCCCAATCAAAACATTGCT

TGA

Amino acid sequence:

MATGKLMIDETHESRGTKRQRSEDVATPIPRNTIKRMLMGDFCKLHIGKEVYVVANMFRG
ETLIHIRTYGKREDGTTFPTKKGIALNLTRWKRLEDRCSGTIDDTIEKVKKGEHIDMKLHL
GGNLHASINSGYGCVNIRRWYMPKDEDGGLVPTRQGIALNFEQWDKLKDCMLLLPDFIG
DELDDVILCKDAHQNQMGMIECFDCNPNQNIA

>Arg0244310.1

Gene sequence:

TCCATACTCTCTCGAGGTAATCTTTCTCCTCTTGTGACAACATCGTGAATGCGTGTTAAC
ATCATATTTGATCTTTTTTATATATACATAAAAACAATAAAAGACTATAATCAGGGTATATT
GATTTTTATTTACAACATTCTACAATATAAAATGCGATTTTCTTATACAGCATTATTGTTAT
GTTCAAACAATATCTTGGTTGGGGTTACAATCAAAACAATCAGTCATTCCCATCTGGTT
CTGATGGCTGTCTTCACACAGTCTCACGTCATCAAGTTCGTCACCGATGAATTCGGGTA
ACAGAAGCATTGAGTCTTTCAGCTTATCCCACTGTTCGAAATTGAGGGCAATCCCTTGC
CTCGTGGGTACCAAATCTCCATCTTCATCCTTGGGCATGTACCACCTCCTGATATTAACA
CAGGCATATCCACTATTTATGCTCGCGTAGATGTTACCACCTAAATGAAACTTCATGTGG
ACGTGTTCCCCTTTCTTGACTTTTTCTATGGCACCGTCGATGTCCTTCGAACACCTGTCC
TCGAGTCTCTTCCATCTCGTGATATTGAGAGCAATACCCTTTTTGGTAGGATACGTGCTT
CCGTCCTCTCTCTTCCCGTAAGTTCTGACGTGGATCAAAGTCTCTCCACGGAACATGTT
AGCCACCACGTAGACTTCTTTGCCTATGTGTATTTTGCAGTAGTCCCCCATCAACATCCT
CTTTATCATGTTTCTGGGTATCGGCGTTGTGGGGTCCTCGCTCCTTTGACGTTTGGTCCC
TCTACTTTCAAAAGTTTCGTCTATCATCAGTTTTCCGGTGGCCATGTTGATTGCAGTGAA
GAATTAGAGTGTGGACCGTTCTTATATACCTTTCACGACGTATTGCACAATCAAGATGA
AATCTGCGGATGTATTTCACAACAGTGTGTTGTTGTGTAATCCGCGAAGTATTGTATGAT
GTTG

Coding sequence:

ATGGCCACCGGAAAACTGATGATAGACGAAACTTTTGAAAGTAGAGGGACCAAACGT
CAAAGGAGCGAGGACCCCACAACGCCGATACCCAGAAACATGATAAAGAGGATGTTG
ATGGGGGACTACTGCAAAATACACATAGGCAAAGAAGTCTACGTGGTGGCTAACATGT
TCCGTGGAGAGACTTTGATCCACGTCAGAACTTACGGGAAGAGAGAGGACGGAAGCA
CGTATCCTACCAAAAAGGGTATTGCTCTCAATATCACGAGATGGAAGAGACTCGAGGA
CAGGTGTTCGAAGGACATCGACGGTGCCATAGAAAAAGTCAAGAAAGGGGAACACGT
CCACATGAAGTTTCATTTAGGTGGTAACATCTACGCGAGCATAAATAGTGGATATGCCT
GTGTTAATATCAGGAGGTGGTACATGCCCAAGGATGAAGATGGAGATTTGGTACCCAC
GAGGCAAGGGATTGCCCTCAATTTCGAACAGTGGGATAAGCTGAAAGACTCAATGCTT
CTGTTACCCGAATTCATCGGTGACGAACTTGATGACGTGAGACTGTGTGAAGACAGCC
ATCAGAACCAGATGGGAATGACTGATTGTTTTGATTGTAACCCCAACCAAGATATTGTT
TGA

Amino acid sequence:

MATGKLMIDETFESRGTKRQRSEDPTTPIPRNMIKRMLMGDYCKIHIGKEVYVVANMFRG
ETLIHVRTYGKREDGSTYPTKKGIALNITRWKRLEDRCSKDIDGAIEKVKKGEHVHMKFH
LGGNIYASINSGYACVNIRRWYMPKDEDGDLVPTRQGIALNFEQWDKLKDSMLLLPEFIG
DELDDVRLCEDSHQNQMGMTDCFDCNPNQDIV
