# Supplementary material for: Tissue-Specific and Time-Dependent Expressions of PC4s in Bay Scallop (Argopecten irradians irradians) Reveal Function Allocation in Thermal Response
Source: Genes (Basel). 2022 Jun 13;13(6):1057. doi: 10.3390/genes13061057 (PMC9223095; doi:10.3390/genes13061057)
Supplement: Supplementary file 1 [file genes-13-01057-s001.zip › Supplementary Table.doc.pdf]

Supplementary Table S1.

The species and accession number for homologous sequence alignment.

Phylum	Species	Protein	Accession number
Vertebrata	*Homo sapiens*	PC4	NP_006704.3
	Mus musculus	PC4	NP_035424.1
	Gallus gallus	PC4	NP_001008479.1
	Xenopus laevis	PC4	XP_018122022.1
	Oryzias latipes	PC4	NP_001098187.1
	Danio rerio	PC4	NP_001002505.1
Urochorda	*Ciona intestinalis*	PC4	XP_002128161.1
Hemichordata	*Saccoglossus kowalevskii*	PC4	XP_002733216.1
**Echinoderma**	*Strongylocentrotus purpuratus*	PC4	XP_001177085.1
	Apostichopus japonicus	PC4	PIK48924.1
**Arthropoda**	*Drosophila serrata*	PC4	XP_020808249.1
	Bombus impatiens	PC4	XP_003485628.1
**Mollusca**	*Pomacea canaliculata*	PC4	XP_025077372.1
	Crassostrea virginica	PC4	XP_022333594.1
Rotifera	*Brachionus plicatilis*	PC4	RNA25207.1
Nematoda	*Strongyloides ratti*	PC4	XP_024504874.1
**Platyhelminthes**	*Echinococcus granulosus*	PC4	XP_024353350.1

**Cnidaria**	***Stylophora pistillata***	PC4	XP_022799284.1
**Placozoa**	***Trichoplax sp. H2***	PC4	RDD43754.1
**Porifera**	***Amphimedon queenslandica***	PC4	XP_003389148.1
